# Correlated Molecular Orbital Theory Predictions of Hydrogen-Containing Halomethane Thermochemistry: Heats of Formation, C–H Bond Dissociation Energies, and p*K*_a_ Values

**DOI:** 10.1021/acs.jpca.5c08066

**Published:** 2026-02-20

**Authors:** Thomas Dalton Andress, Cole Seely, Margherita Miele, Laura Castoldi, Vittorio Pace, David A. Dixon

**Affiliations:** † Department of Chemistry and Biochemistry, Shelby Hall, 8063 The University of Alabama, Tuscaloosa, Alabama 35487, United States; ‡ 9314 University of Turin, Department of Chemistry, via Giuria 7, Turin 10125, Italy; § 9304 University of Milan, Department of Pharmaceutical Sciences, General and Organic Chemistry Section “A. Marchesini”, via Venezian 21, Milan 20133, Italy; ∥ University of Rome “La Sapienza”, Department of Chemistry, P.le A. Moro 5, Rome 00185, Italy; ⊥ University of Vienna, Department of Pharmaceutical Sciences, Division of Pharmaceutical Chemistry, Josef-Holaubek-Platz 2, Vienna 1090, Austria

## Abstract

Heats of formation, bond dissociation energies, proton affinities, gas phase acidities, and p*K*_a_ values in water, dimethyl sulfoxide, acetonitrile, and tetrahydrofuran were calculated for all hydrogen-containing halomethanes and methane using composite correlated molecular orbital theory at the G3­(MP2) and Feller-Peterson-Dixon (FPD) levels. Notably, the G3­(MP2) method was extended to include iodine-containing compounds. The calculated gas phase acidities generally agree with available experimental data within experimental error limits, often within ±4 kJ/mol; however, CH_2_F_2_ is a significant exception where theory and experiment differ by nearly 40 kJ/mol for the acidity Δ*G*. Aqueous p*K*_a_ values range from 53.6 for CH_3_F to 28.0 for CHF_2_I. The latter’s unexpectedly high acidity results from the CF_2_I^–^ anion resembling a CF_2_ carbene interacting with an iodide anion. These computed values rationalize literature base choices for anion generation: trihalomethanes (p*K*_a_ 28.0–34.2) are deprotonated by nonorganometallic bases (KOH, DBU, KOtBu), whereas less acidic dihalomethanes (p*K*_a_ ≳ 38), particularly fluorodihalomethanes (p*K*_a_ 42–49), require strong metal amides (e.g., LTMP, LDA), with LHMDS proving inadequate. An experimental CHBrCl_2_ case study corroborates these predictions, showing clean deprotonation with lithium amides compared to diminished efficiency with weaker bases due to competitive hydroxide addition. This work provides the most comprehensive high-accuracy thermochemical data set for the complete set of hydrogen-containing halomethanes.

## Graphical abstract

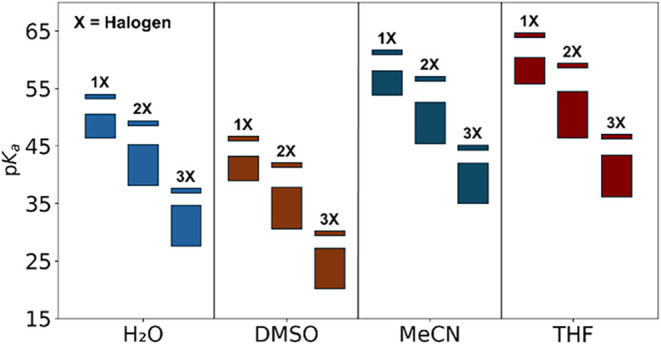

## Introduction

Estimates of the aqueous acidity constants (p*K*_a_s) of hydrohalomethanes^1–4^ are available for only a few of the 35 compounds even though this class of compounds is one of the most prevalent within organic chemistry with multiple examples likely present in every laboratory. Many of the gas phase C–H bond dissociation energies of the halomethanes are available from Luo’s compilation^5^ with some species having more reliable values available from the Active Thermochemical Tables (ATcT).^6–8^ Nearly half of the hydrogen-containing halomethanes have experimental values for their gas phase acidity^9–15^ as summarized in the NIST Webbook.^16^ The properties of all halomethane species need to be established as both the Toxic Substances Act and the American Innovation and Manufacturing Act look to limit their availability. Dichloromethane is already being phased out of use by the EPA due to its carcinogenic nature.^17,18^

The inherent acidity of trihalo- and dihalo-methanes makes them excellent precursors for often elusive trihalo- and dihalomethyl- anions.^19,20^ These species, referred to as carbenoids, constitute a *unicum* in chemistry, as a consequence of the intrinsic capability to shift from nucleophilic to electrophilic and *vice versa*.^21^ This characteristic renders carbenoids key players in C1-insertion processes, as homologations.^22^ Besides techniques based on halogen/metal exchange reactions,^23^ (poly)­halomethyl anions can be conveniently prepared through deprotonation of the corresponding haloforms or methylene halides.^21,24^ Obtaining approximate p*K*_a_ acidity for these species is critical for selecting the most adequate base enabling the genesis of the desired carbenoid. In fact, both the reactivity regime (nucleophilic or electrophilic) and the chemical integrity can be finely modulated by the countercation.^25,26^ For example, lithium carbenoids, generated in the presence of organolithiums, predominantly exhibit nucleophilic properties,^21^ whereas moving to less positive metals (e.g., Mg, Zn) moves them into the electrophilic regime.^27–29^

Despite their ubiquity, three major gaps undermine the current understanding of halomethane thermodynamics. First, no unified data set exists for all possible halogen combinations, particularly for iodine-containing species due to calculation limitations and the complexities of experimental evaluation such as reactant volatility.^30^ Second, the traditional G3­(MP2)^31,32^ approach does not incorporate iodine which limits the accessibility of low-cost, high-accuracy calculations to nearly half of the hydrohalomethanes. Third, solvent effects in combination with halogen substitution effects remain poorly quantified, as evidenced by the very limited reporting of both experimental and theoretical p*K*_a_ values of the hydrohalomethanes. This work seeks to address these gaps by advancing the G3­(MP2) model for use in iodomethanes while critically evaluating its accuracy through comparison to available high accuracy experimental data from ATcT as well as the other experimental data.

These knowledge gaps slow progress across multiple domains. Atmospheric modelers lack reliable data for iodine-mediated ozone depletion cycles, materials scientists face uncertainties in designing next-generation halocarbon refrigerants, and synthetic chemists lack information about what is the appropriate choice of a strong base for the generation of carbenoids. Even fundamental theoretical frameworks, such as linear free-energy relationships for halogen substituent effects, remain incomplete without comprehensive thermochemical data spanning the entire halogen series.

Thermochemical parameters such as aqueous p*K*_a_, gas-phase acidity, enthalpy of formation, and bond dissociation energy serve as foundational metrics for predicting chemical reactivity, environmental persistence, and industrial utility. For halomethanes, these properties dictate, in part, their efficacy as refrigerants, solvents, and fire suppressants while simultaneously determining their ozone-depletion potential and global warming contributions. The environmental significance of halomethanes further underscores the urgency of accurate thermochemical characterization. Chlorofluorocarbons (CFCs) and hydrochlorofluorocarbons (HCFCs), once widely used in refrigeration, exemplify how incomplete thermodynamic understanding can lead to unintended environmental consequences.^33^ Their atmospheric lifetimes and ozone-depleting potentials are direct functions of bond dissociation energies and reaction enthalpies.^34^ These parameters remain minimally studied for mixed-halogen species. Fourth generation refrigerants such as iodine-containing halomethanes present both opportunities as environmentally benign alternatives and challenges due to gaps in their thermochemical profiles.

Experimental determinations of gas-phase acidities have shown intricate substituent effects, where electronegative halogens stabilize conjugate bases through inductive withdrawal while polarizable atoms like iodine enhance acidity via charge delocalization.^16^ However, measurements become increasingly challenging for thermally labile or low-volatility species, necessitating reliance on computational predictions as evidenced by the lack of iodine data.

A recent study by Awad^35^ employed a combination of experimental data and computational quantum chemistry to evaluate halomethane thermodynamics. Ab initio methods, including Hartree–Fock and composite approaches together with density functional theory (DFT), provided high accuracy predictions of the enthalpies of formation and bond dissociation energies for fluorine-, chlorine-, and bromine-substituted methanes, achieving mean absolute deviations of ≤ 5 kJ/mol from experimental benchmarks although iodine-containing species were not included nor were any energies in solution reported.

## Computational and Experimental Methods

### Computational Details

All geometries were initially optimized at the density functional theory (DFT) level using the B3LYP^36,37^ functional. These optimized geometries were then used as inputs for optimizations at the MP2 level.^38,39^ The DFT and MP2 optimizations were both performed with the aug-cc-pVTZ (aT) basis set^40,41^ with pseudopotentials (-PP) for Br and I.^42–44^ Frequency calculations were performed at both the B3LYP/aT and MP2/aT levels to confirm minima and to obtain zero-point energies (ZPE) and thermal corrections. All optimization and frequency calculations were performed using Gaussian16.^45^ The composite correlated molecular orbital theory G3­(MP2)^31,32^ was then used to predict gas-phase thermochemical values for species not containing iodine as implemented in Gaussian16.

For species containing iodine, we use a modified G3­(MP2) approach where the HF optimization and frequency analysis and single point QCISD­(T,FC) calculation uses cc-pVDZ-PP basis set for I. The MP2­(Full) optimization calculation uses the weighted core basis set cc-pwCVDZ-PP for I, and the single point MP2­(FC) calculation uses the weighted core basis set with diffuse functions aug-cc-pwCVTZ-PP for I. All other aspects of the G3­(MP2) calculation were kept the same including the high level correction (HLC) values. To the best of our knowledge, this research represents the first instance of an extended G3­(MP2) approach with unmodified HLC values.

Using the optimized geometries from the MP2/aT­(-PP) level, energies were obtained at the coupled cluster singles and doubles with perturbative triples (CCSD­(T))^46^ level using the aug-cc-pVnZ (aN, N = T, Q, 5) basis sets.^40,41,47^ All CCSD­(T) calculations were performed using the MOLPRO software package.^48,49^ Open shell calculations were performed with UCCSD­(T). Extrapolations to the CBS limit were obtained using eq 1 for two-point extrapolations with *n* = TQ and Q5 and eq 2 for three-point extrapolations with *n* = TQ5.^50,51^

1 $$E_{n}=E_{\mathrm{CBS}}+A{\left(n+1/2\right)}^{-4}$$

2 $$E_{n}=E_{\mathrm{CBS}}+A\exp\left[-\left(n-1\right)\right]+B\exp\left[-{\left(n-1\right)}^{2}\right]$$

Both the scalar relativistic corrections (Δ*E*_SR_) and the core-valence corrections (Δ*E*_CV_) were obtained at CCSD­(T) level. The Δ*E*_SR_ were calculated at the third order Douglas-Kroll-Hess (DK)^52–55^ level with the all-electron basis set aug-cc-pwCVTZ-DK (aT-DK) using eq 3.

3 $$\Delta E_{\mathrm{SR}}=\Delta E_{\mathrm{awCT}-\mathrm{DK}}-\Delta E_{\mathrm{awCT}\left(-\mathrm{PP},\mathrm{no}\;c\mathrm{orrelation}\right)}$$

The Δ*E*_CV_ were calculated with aug-cc-pwCVTZ­(‑PP) (awCT­(‑PP)) using eq 4.

4 $$\Delta E_{\mathrm{CV}}=\Delta E_{\mathrm{awCT}\left(-\mathrm{PP}\right)}-\Delta E_{\mathrm{awCT}\left(-\mathrm{PP},\mathrm{no}\;\mathrm{correlation}\right)}$$

The keyword “CORE,SMALL” in MOLPRO was used when calculating the core-valence correction. C–H frequencies from MP2/aT were scaled by 0.95 to better account for anharmonicity. This scaling value was chosen because it gives the best agreement with spectroscopically derived ZPE values for these species.^56^ The scaled ZPE was applied to CCSD­(T) energies. The CCSD­(T) results were used in the Feller-Peterson-Dixon (FPD) approach^57–60^ to total dissociation energies, heats of formation, C–H BDE, gas phase proton affinities, and gas phase acidities. Total dissociation energies (*D*_0_) were calculated according to eq 5.

5 $$D_{0}=\sum E_{\mathrm{CBS}}\;(atoms)-E_{\mathrm{CBS}}\;\left(\mathrm{molecule}\right)+\Delta E_{\mathrm{ZPE}}+\Delta E_{\mathrm{SR}}+\Delta E_{\mathrm{CV}}+\Delta E_{\mathrm{SO}}$$

Heats of formation at 298.15 K were calculated by following the procedures outlined by Curtiss et al.^61^ using 4.23, 1.05, 4.39, 4.60, 12.26, and 6.61, kJ/mol thermal corrections and 0, −0.33, −1.63, −3.51, −16.69, and −30.33 kJ/mol spin orbit values for H, C, F, Cl, Br, and I, respectively.

Single point solvation calculations with B3LYP and MP2 optimized geometries were performed at the B3LYP/aT and MP2/aT levels using the self-consistent reaction field (SCRF) approach^62^ with the conductor like screening model (COSMO)^63,64^ and the solvation model based on density (SMD)^65^ which includes additional nonelectrostatic terms compared to COSMO.

There are two ways to represent acidity. The first is absolute where the proton contribution is put directly into the calculation (Reaction 6). The other is relative where an experimentally known acid is used in the reaction (Reaction 7).

R1 $${\mathrm{CHX}}_{3}\to {\mathrm{CX}}_{3}^{-}+H^{+}$$

R2 $${\mathrm{CHX}}_{3}+{\mathrm{Ac}}^{-}\to {\mathrm{CX}}_{3}^{-}+\mathrm{AcH}$$

For gas phase acidity, reaction 6 is used. Solution-phase p*K*_a_ values reported in the main text are absolute, obtained from Reaction 6 by combining gas-phase deprotonation free energies with solvation free. For comparison and validation, relative results based on Reaction 7 referenced to benzoic acid are provided in the Supporting Information. Benzoic acid was selected as the reference acid for this study due to the availability of reliable experimental p*K*_a_ values across all four solvent systems examined (water, DMSO, MeCN, and THF). Furthermore, the delocalized charge distribution of the benzoate anion is more accurately described by continuum solvation models than that of small, hard oxyanions, minimizing systematic errors associated with the implicit solvation of localized charges. We use p*K*_a_ values of 4.25 in water,^66^ 21.5 in MeCN,^67^ 25.11 in THF,^66^ and 11.1 in DMSO^68^ for benzoic acid. eq 8 is used to account for the standard state correction which represents the energy change associated with compressing 1 mol of an ideal gas at a presume of 1 atm to a concentration of 1 M.

6 $${\Delta G}^{{}^{\circ}\to *}=R\times T\times \ln\left(24.46\right)$$

In eq 8, *R* is the gas constant, *T* is temperature (298.15 K), and the value 24.46 represents the volume (in liters) occupied by 1 mol of an ideal gas at 298.15 K and 1 atm.

The free energy of solvation is calculated as the difference between the total energy (EE) in solution and the total energy in the gas phase plus the standard state correction as shown in eq 9.

7 $${\Delta G^{*}}_{solv}={{\mathrm{EE}}^{*}}_{\mathrm{soln}}-{{\mathrm{EE}}^{{}^{\circ}}}_{\mathrm{gas}}+{\Delta G}^{o\to *}$$

The absolute solvation free energies of the proton, ΔG*_solv_(H^+^), were selected from high-level studies utilizing cluster-continuum methodologies to account for explicit solvent–solute interactions. All values reported herein correspond to a standard state of 1 M in the gas phase transferring to 1 M in solution; therefore, these values already include the state change energy calculated using eq 8.

For water we use an aqueous ΔG*_solv_ value of – 1097.9 kJ/mol. This is a benchmark value from Zhan and Dixon, derived via a hybrid supermolecule-continuum approach at the CCSD­(T)/CBS level which explicitly treats first-shell nonelectrostatic interactions and which has provided good results for previous p*K*_a_ studies.^69–71^ For DMSO (−1143.5 kJ/mol), the value is taken from Kelly and Truhlar, utilizing cluster-pair approximations validated against independent high-level theoretical estimates.^72^ Values for acetonitrile (−1059.4 kJ/mol) and THF (−1075.3 kJ/mol) were taken from recent work utilizing cluster-continuum calculations that account for Boltzmann weighting and entropy of mixing; these values were validated by reproducing the experimental standard hydrogen electrode potentials in their respective solvents.^73^

The solvation contribution (ΔΔG*_solv_) to the free energy in solution is calculated using eq 10.

8 $${\Delta \Delta G^{*}}_{\mathrm{solv}}={\Delta G^{*}}_{\mathrm{solv}}\left[X_{\mathrm{prod}.}\right]-{\Delta G^{*}}_{\mathrm{solv}}\left[X_{\mathrm{react}.}\right]$$

The aqueous free energy in solution (ΔG*_aq_) is calculated using eq 11.

9 $${\Delta G^{*}}_{\mathrm{aq}}={\Delta \Delta G^{*}}_{\mathrm{solv}}+\Delta {G^{{}^{\circ}}}_{\mathrm{gas}}$$

Where Δ*G°*_gas_ is the gas phase free energy of the reaction at 298.15 K obtained with B3LYP/aT, MP2/aT, G3­(MP2), FPD. ΔΔ*G*_solv_ contributions for Δ*G*_aq_(G3­(MP2)) and Δ*G*_aq_(FPD) were calculated using MP2/aT/SCRF. Aqueous p*K*_a_ is calculated with ΔG*_aq_ using eq 12.

10 $$pK_{a}={\Delta G^{*}}_{\mathrm{aq}}/\left(R\times T\times \ln\left(10\right)\right)$$

where *R* is the gas constant and *T* is temperature. For relative acidities, the experimentally known p*K*_a_ is subtracted from the resulting p*K*_a_ from eq 12.

A Natural Population Analysis (NPA) based on the Natural Bond Orbital (NBO) method^74,75^ using NBO 7 ^76^ was performed on the anions at the MP2/aT level.

### Experimental Details

Melting points were determined on a Reichert-Kofler hot-stage microscope and are uncorrected. Mass spectra were obtained on a Shimadzu QP 1000 instrument (EI, 70 eV) and on a Bruker maXis 4G instrument (ESI-TOF, HRMS). ^1^H, ^13^C and ^19^ F NMR spectra were recorded on a Bruker Avance III 400 spectrometer (400 MHz for ^1^H, 100 MHz for ^13^C, 40 MHz for ^15^N, 376 MHz for ^19^F) at 297 K using a, directly detecting broadband observe (BBFO) probe. The center of the solvent signal was used as an internal standard which was related to TMS with δ 7.26 ppm (^1^H in CDCl_3_), δ 77.00 ppm (^13^C in CDCl_3_). ^15^N spectra (gsHMBC) were referenced against neat, external nitromethane, ^19^F NMR spectra by absolute referencing via Ξ ratio. Spin–spin coupling constants (*J*) are given in Hz. In nearly all cases, full and unambiguous assignment of all resonances was performed by combined application of standard NMR techniques, such as APT, HSQC, HMBC, COSY and NOESY experiments.

All of the reactions were carried out under an inert atmosphere of argon. THF was distilled over Na/benzophenone. Chemicals were purchased from Sigma-Aldrich, Acros, Alfa Aesar and TCI Europe. Solutions were evaporated under reduced pressure with a rotary evaporator. TLC was carried out on aluminum sheets precoated with silica gel 60F254 (Merchery-Nagel, Merk); the spots were visualized under UV light (λ = 254 nm).

Specific reaction details and the associated spectra are given in the Supporting Information (SI).

## Computational Results and Discussion

### Gas Phase Δ*H*_f_^0^ and BDE

The calculated FPD heats of formation (Table 1) are for in excellent agreement with the experimental values from ATcT^6^ with the largest deviation being 6.5 kJ/mol for CHBr_2_F; note that the experimental value has a larger error bar as well. The largest deviations from experiment at the G3­(MP2) level are for the trihalogens CHBr_3_, CHI_3_, CHCl_2_Br, and CHBr_2_Cl which differ by −9 to −11 kJ/mol. Good agreement is found between the G3­(MP2) and FPD values with a median absolute difference of 4 kJ/mol highlighting the quality of our modified G3­(MP2) approach. CHI_3_ is subject to second order spin orbit effects which can be present in closed shell molecules with heavy atoms. For CHI_3_, we applied a previously calculated spin orbit correction of −6.3 kJ/mol obtained at the CASPT2/RASSI-SO.^77^ An attempt to calculate the spin–orbit correction for CHI_3_ using the ADF software^78,79^ led to an overestimate of the SO effect. The B3LYP/aT heats of formation are not reliable, and the error tends to grow with increasing number of halogen atoms with a largest error of 77 kJ/mol.

**Table 1:** Gas Phase Heat of Formation at 298 K in kJ/mol

species	B3LYP/aT	G3(MP2)	FPD/Q5	expt. ATcT^6^	Δ*E*_expt_ (G3(MP2))	Δ*E*_expt_ (FPD)
CH_4_	–75.0	–74.4	–74.1	–74.51 ± 0.043	0.1	0.5
CH_3_F	–232.3	–236.0	–234.7	–235.47 ± 0.24	–0.5	0.8
CH_2_F_2_	–438.8	–451.3	–449.7	–450.93 ± 0.35	–0.4	1.3
CHF_3_	–671.4	–696.6	–695.3	–696.23 ± 0.4	–0.4	1.0
CH_3_Cl	–66.2	–81.9	–83.5	–82.58 ± 0.17	0.7	–0.9
CH_2_Cl_2_	–57.1	–95.3	–96.4	–93.7 ± 0.33	–1.6	–2.7
CHCl_3_	–37.8	–105.8	–103.8	–99.42 ± 0.39	–6.4	–4.4
CH_3_Br	–18.7	–37.0	–35.2	–35.85 ± 0.19	–1.2	0.7
CH_2_Br_2_	43.0	0.6	5.8	5.61 ± 0.59	–5.0	0.2
CHBr_3_	116.7	43.7	53.9	54.83 ± 0.7	–11.2	–0.9
CH_3_I	35.6	16.2	17.6	14.99 ± 0.16	1.2	2.6
CH_2_I_2_	157.3	109.0	114.9	113.52 ± 0.78	–4.5	1.4
CHI_3_	286.0	198.4	212.3	209.3 ± 1.6	–10.9	3.0
CH_2_FCl	–242.5	–264.4	–264.8	–263.63 ± 0.85	–0.8	–1.1
CH_2_FBr	–189.6	–212.3	–209.5	–211.9 ± 4.9	–0.4	2.4
CH_2_FI	–127.8	–150.8	–147.4			
CH_2_ClBr	–6.9	–47.1	–45.0	–42.3 ± 1.3	–4.8	–2.7
CH_2_ClI	51.2	8.3	11.4			
CH_2_BrI	100.6	52.8	61.2			
CHF_2_Cl	–450.9	–483.7	–483.1	–481.76 ± 0.99	–1.9	–1.3
CHF_2_Br	–394.4	–426.7	–422.9	–424.59 ± 0.47	–2.1	1.7
CHF_2_I	–328.5	–360.3	–355.3			
CHCl_2_F	–239.5	–286.8	–285.8	–283.07 ± 0.9	–3.8	–2.7
CHCl_2_Br	13.8	–55.7	–50.8	–46.5 ± 1.2	–9.2	–4.3
CHCl_2_I	73.5	–0.2	7.0			
CHBr_2_F	–131.3	–179.7	–172.8	–179.3 ± 4.9	–0.4	6.5
CHBr_2_Cl	65.3	–5.9	1.8	3.2 ± 3.5	–9.1	–1.4
CHBr_2_I	175.7	93.1	110.0			
CHI_2_F	–6.7	–59.5	–50.2			
CHI_2_Cl	183.6	104.0	114.6			
CHI_2_Br	234.3	149.6	164.9			
CHBrClF	–185.4	–233.2	–229.2	–230.9 ± 4.9	–2.3	1.7
CHIClF	–122.5	–172.2	–166.4			
CHIBrF	–68.8	–121.7	–110.9			
CHIBrCl	124.7	46.6	58.8			

The C–H BDEs are not as well established as the heats of formation with substantially larger error bars. The FPD calculated C–H bond dissociation energies (BDE) are within 4 kJ/mol of the high accuracy experimental ATcT^6^ values (Table 2) as are the G3­(MP2) values. For the experimental BDEs from Luo,^5^ the calculated FPD values are within or just outside the error bars for many of the species. Notable exceptions are CH_2_I_2_ and CHI_3_, which differ from reported values by 26 and 40.5 kJ/mol, respectively. However, we suspect these deviations stem from issues within the experimental compilation rather than computational inaccuracy. The experimental BDE and associated error limits for CH_2_I_2_ (431.0 ± 8.4 kJ/mol) are numerically identical to those reported for CH_3_I in the same reference, strongly suggesting a transcription error in the literature. The experimental value for CHI_3_ carries an exceptionally large uncertainty (±29 kJ/mol). In contrast, the modified G3­(MP2) method shows excellent internal consistency with our high-level FPD/Q5 benchmarks for these species, deviating by only 1.1 kJ/mol for CH_2_I_2_ and 1.5 kJ/mol for CHI_3_. This suggests the computational results provide a necessary correction to the available experimental data. Another example of an ∼20 kJ/mol difference is CHBr_2_Cl but the error bar here is ± 21 kJ/mol. The differences between the calculated values and experiment for CH_2_Br_2_, CHBr_3_, and CH_3_I are on the order of ±10 kJ/mol, consistent with the larger error bars on the order of ±8 kJ/mol. B3LYP predicts BDEs within about 10 kJ/mol of the FPD values showing a cancellation of error between the parent and the radical.

**Table 2:** Gas Phase C–H Bond Dissociation Energy (Δ*H*) at 298 K in kJ/mol

species	B3LYP/aT	G3(MP2)	FPD/Q5	expt. Luo^5^	Δ*E*_expt_ (G3(MP2))	Δ*E*_expt_ (FPD)
CH_4_	431.7	435.7	436.5	438.99 ± 0.065^a^	–3.3	–2.5
CH_3_F	413.1	422.6	421.8	423.8 ± 4.2	–1.2	–2.0
CH_2_F_2_	413.0	424.6	424.1	431.8 ± 4.2	–7.2	–7.7
CHF_3_	432.7	446.2	445.2	446.26 ± 0.6^a^	–0.1	–1.0
CH_3_Cl	413.5	415.7	413.6	419.0 ± 2.3	–3.3	–5.4
CH_2_Cl_2_	391.4	402.2	400.0	402.7 ± 0.94^a^	–0.5	–2.7
CHCl_3_	384.9	391.7	389.2	392.5 ± 2.5	–0.8	–3.3
CH_3_Br	418.4	420.8	420.5	427.2 ± 2.4	–6.4	–6.7
CH_2_Br_2_	402.2	406.5	407.4	417.1 ± 7.5	–10.6	–9.7
CHBr_3_	384.1	391.0	392.4	401.7 ± 6.7	–10.7	–9.3
CH_3_I	420.5	421.9	421.6	431.0 ± 8.4	–9.1	–9.4
CH_2_I_2_	400.5	403.7	404.8	431.0 ± 8.4	–27.3	–26.2
CHI_3_	376.9	382.5	384.0	423 ± 29	–40.5	–39.0
CH_2_FCl	403.1	414.5	413.0	421.7 ± 10.0	–7.2	–8.7
CH_2_FBr	411.3	416.3	416.1			
CH_2_FI	410.0	414.5	414.5			
CH_2_ClBr	400.1	404.4	403.8	406.0 ± 2.4	–1.6	–2.2
CH_2_ClI	399.3	402.2	402.5			
CH_2_BrI	401.4	404.6	406.1			
CHF_2_Cl	418.7	426.0	424.7	421.3 ± 8.4	4.7	3.4
CHF_2_Br	413.8	421.9	421.5	415.5 ± 12.6	6.4	6.0
CHF_2_I	404.9	413.3	413.1			
CHCl_2_F	401.5	408.7	406.8	410.9 ± 8.4	–2.2	–4.1
CHCl_2_Br	384.6	391.4	390.2	387 ± 21	4.4	3.2
CHCl_2_I	381.1	386.5	386.3			
CHBr_2_F	397.4	405.4	405.7			
CHBr_2_Cl	384.3	391.1	391.2	371 ± 21	20.1	20.2
CHBr_2_I	381.2	387.2	389.2			
CHI_2_F	388.2	396.4	396.9			
CHI_2_Cl	378.4	384.0	385.0			
CHI_2_Br	378.8	384.7	386.9			
CHBrClF	399.4	406.9	406.1	413 ± 21	–6.1	–6.9
CHIClF	393.8	400.9	400.7			
CHIBrF	392.4	400.2	400.7			
CHIBrCl	381.1	386.8	387.6			

a Experimental values from ATcT.^6^

All of the C–H BDEs except for CHF_3_ are less than that for CH_4_. The presence of the halogen tends to stabilize the corresponding methyl radical. An increase in the number of halogen substitutions for Cl, Br, and I led to a decrease in the C–H bond energies. This decrease also occurs with increasing halogen size. There is an increase in the C–H bond energy from CH_3_F to CHF_3_. We examined different correlations of the BDEs with other electronic properties. The C–H BDE is plotted against the isotropic polarizability of the corresponding methyl radical in Figure 1. While the global data set suggests a general trend (*R* ^2^ = 0.736), plotting the data by substitution level reveals distinct electronic behaviors. The monohalomethanes show essentially no correlation (*R* ^2^ = 0.019), indicating that polarizability is not a primary determinant of bond strength for these species. However, a correlation emerges for the dihalo species (*R* ^2^ = 0.461) and strengthens significantly for the trihalo species (*R* ^2^ = 0.738). This progression indicates that the stabilizing effect of radical polarizability is not universal. Instead, it becomes increasingly dominant with higher degrees of halogen substitution.C–H Plots of the BDE vs the spin density on the carbon of the radical product (*R* ^2^ = 0.11) or the dipole moment of the radical (*R* ^2^ = 0.10) did not yield meaningful correlations.

**Figure 1:**
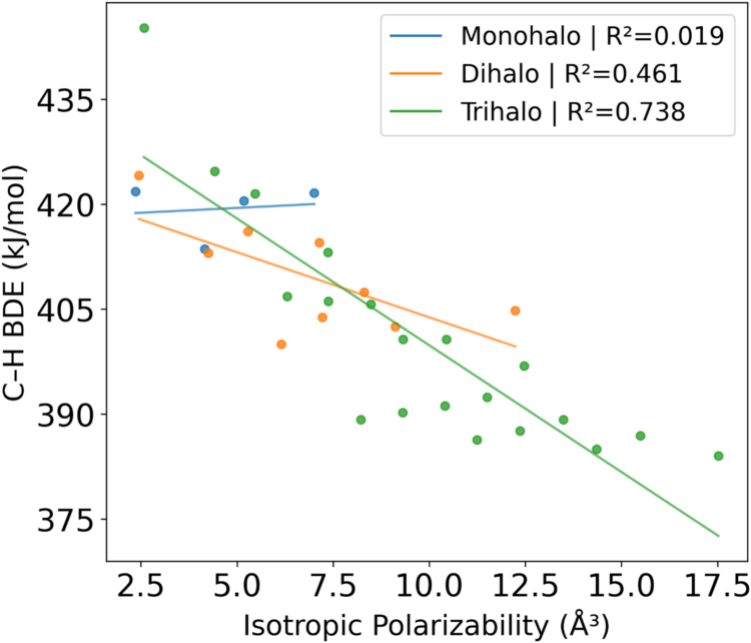
BDE versus isotropic polarizability of the radical resulting from breaking a C–H bond. BDE values from FPD with polarizability from MP2/aT.

### Gas Phase Electron Affinity of CXYZ^•^

As the energies for both radical and anion CXYZ species are available, one can readily calculate the electron affinity of the radical (Table 3). The electron affinities are the adiabatic values including ZPE corrections. There is excellent agreement of the FPD values with the experimental values from the NIST Webbook^16^ within 0.1 eV for all species except for CHF_2_^•^ which, again, is a species for which the experimental data should be revisited. For CH_2_Br^•^, there are two experimental values^11,80^ and there is better agreement between theory and experiment for the larger value.^11^ Good agreement, usually within 0.1 eV, is also found between the FPD and G3­(MP2) values. A similar trend is found for the electron affinity as with the previous properties where there is an increase in the EA with increasing halogen substitution and an increasing halogen size.

**Table 3:** Electron Affinity (Δ*H*) at 0 K in eV

species	B3LYP/aT	G3(MP2)	FPD/Q5	expt. NIST^16^	Δ*E*_expt_ (G3(MP2))	Δ*E*_expt_ (FPD)
CH_3_^•^	0.11	0.01	0.07	0.080 ± 0.030	–0.07	–0.01
CH_2_F^•^	0.26	0.20	0.19	0.25 ± 0.18	–0.05	–0.06
CHF_2_^•^	0.77	0.73	0.70	1.21 ± 0.16	–0.48	–0.51
CF_3_^•^	1.83	1.77	1.76	1.820 ± 0.050	–0.05	–0.06
CH_2_Cl^•^	0.73	0.75	0.66	0.74 ± 0.16	0.01	–0.08
CHCl_2_^•^	1.48	1.50	1.40	1.472 ± 0.043	0.03	–0.08
CCl_3_^•^	2.15	2.18	2.06	2.160 ± 0.096	0.02	–0.10
CH_2_Br^•^	0.94	0.98	0.91	0.79 ± 0.14	0.19 0.01	0.12 −0.06
				0.97 ± 0.16		
CHBr_2_^•^	1.78	1.82	1.76			
CBr_3_^•^	2.43	2.48	2.41	2.57 ± 0.12	–0.09	–0.16
CH_2_I^•^	1.16	1.18	1.14			
CHI_2_^•^	2.01	2.04	2.01			
CI_3_^•^	2.58	2.64	2.60			
CHFCl^•^	1.24	1.22	1.13			
CHFBr^•^	1.46	1.43	1.38			
CHFI^•^	1.65	1.58	1.58			
CHClBr^•^	1.64	1.66	1.58			
CHClI^•^	1.77	1.78	1.73			
CHBrI^•^	1.90	1.93	1.89			
CF_2_Cl^•^	2.17	2.06	2.02			
CF_2_Br^•^	2.38	2.30	2.28			
CF_2_I^•^	2.55	2.48	2.51			
CCl_2_F^•^	2.21	2.17	2.07			
CCl_2_Br^•^	2.26	2.29	2.18			
CCl_2_I^•^	2.34	2.35	2.26			
CBr_2_F^•^	2.47	2.44	2.38			
CBr_2_Cl^•^	2.35	2.39	2.30			
CBr_2_I^•^	2.49	2.54	2.47			
CI_2_F^•^	2.64	2.59	2.55			
CI_2_Cl^•^	2.47	2.50	2.44			
CI_2_Br^•^	2.54	2.59	2.54			
CBrClF^•^	2.35	2.30	2.23			
CIClF^•^	2.46	2.37	2.34			
CIBrF^•^	2.56	2.51	2.48			
CIBrCl^•^	2.42	2.45	2.37			

### Gas Phase Acidity

The FPD calculated gas phase acidities are in excellent agreement with experimental values^9–16^ (Table 4) with most values within 2 kJ/mol of experiment. Species which differ by more are still within the error of the experiment except for CH_2_F_2_ (Δ*E*_expt_ = 44.1 kJ/mol) and CHF_2_Cl (Δ*E*_expt_ = −39.6 kJ/mol). However, there are large error bars for these species of ± 15 kJ/mol for CH_2_F_2_ and ± 30 kJ/mol for CHF_2_Cl and we suggest that these experimental values should be revisited. There is good agreement between the G3­(MP2) and FPD acidities with a maximum difference of 8.4 kJ/mol between methods and a median absolute difference of 5.7 kJ/mol. B3LYP is able to predict the acidity across the series reasonably well. The gas phase acidity is predicted to decrease with increasing halogen substitution and halogen size.

**Table 4:** Gas Phase Acidity (Δ*G*) at 298 K in kJ/mol

species	B3LYP/aT	G3(MP2)	FPD/Q5	expt.	Δ*E*_expt_ (G3(MP2))	Δ*E*_expt_ (FPD)
CH_4_	1704.4	1716.8	1710.0	1715 ± 15 ^9^	1.8	–5.0
CH_3_F	1671.5	1685.6	1683.2	1676 ± 17 ^9^	9.6	7.2
CH_2_F_2_	1624.5	1639.5	1639.1	1595 ± 15 ^9^	44.5	44.1
CHF_3_	1542.5	1559.8	1557.3	1549 ± 6.3 ^10^	10.8	8.3
CH_3_Cl	1619.0	1624.5	1629.0	1628 ± 13 ^11^	–3.5	1.0
CH_2_Cl_2_	1533.9	1541.3	1547.0	1540 ± 8.4 ^12^	1.3	7.0
CHCl_3_	1458.2	1465.0	1472.1	1464 ± 8.4 ^13^	1.0	8.1
CH_3_Br	1602.7	1607.0	1611.3	1614 ± 13 ^11^	–7.0	–2.7
CH_2_Br_2_	1507.1	1512.6	1519.6	1512 ± 13 ^12^	0.6	7.6
CHBr_3_	1427.2	1437.3	1441.1	1431 ± 8.4 ^12^	6.3	10.1
CH_3_I	1584.2	1588.7	1590.3	1587 ± 20 ^11^	1.7	3.3
CH_2_I_2_	1483.3	1487.8	1492.4			
CHI_3_	1405.2	1409.9	1411.7			
CH_2_FCl	1569.1	1577.5	1585.6	1576.1 ± 1.3 ^14^	1.4	9.5
CH_2_FBr	1549.3	1557.3	1565.1			
CH_2_FI	1528.7	1540.9	1543.6			
CH_2_ClBr	1520.6	1527.0	1532.8	1528 ± 13 ^12^	–1.0	4.8
CH_2_ClI	1506.5	1512.4	1517.4			
CH_2_BrI	1496.2	1501.1	1505.4			
CHF_2_Cl	1486.4	1506.4	1510.4	1550 ± 30 ^15^	–43.6	–39.6
CHF_2_Br	1460.5	1477.2	1482.2			
CHF_2_I	1434.2	1450.9	1449.7			
CHCl_2_F	1466.3	1481.2	1488.7	1475 ± 8.4 ^14^	6.2	13.7
CHCl_2_Br	1444.7	1453.9	1460.9			
CHCl_2_I	1433.5	1442.0	1449.6			
CHBr_2_F	1435.8	1449.5	1457.0			
CHBr_2_Cl	1435.3	1443.8	1450.6			
CHBr_2_I	1418.8	1425.2	1431.6			
CHI_2_F	1410.4	1426.4	1431.7			
CHI_2_Cl	1417.3	1424.6	1430.6			
CHI_2_Br	1411.6	1417.1	1422.8			
CHBrClF	1449.9	1463.7	1472.1			
CHIClF	1433.4	1450.0	1455.9			
CHIBrF	1421.9	1437.0	1442.6			
CHIBrCl	1425.6	1433.4	1440.2			

Figure 2 plots gas phase acidity versus the Mulliken charge on the carbon of the anion with groupings based on the number of halogen substituents and the amount of fluorination. Most *R* ^2^ values for these groups are well above 0.9. The CHXF_2_ trihalo group has a negative slope as a result of the charge now being positive on the carbon. Fluorine substitution is seen to dominate the charge on the carbon as evidenced by the relative slopes of the groups. For example, for the dihalo fluorine group and the dihalo group without F, the slope is significantly steeper for fluorine containing species (*m* = 658 vs 162). As a specific example, the difference between the charge on the carbon for CH_2_F_2_ and CH_2_FI is Δ = 0.13 versus the difference of Δ = 0.30 for CH_2_Cl_2_ and CH_2_I_2_.

**Figure 2:**
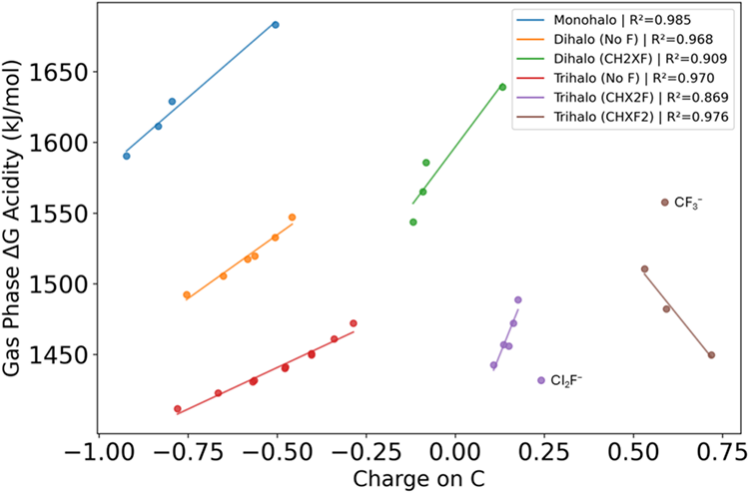
Gas phase acidity versus the NPA charge on the carbon of the resulting anion (MP2/aT) grouped by halogen substitution and fluorination. Methane not shown. CI_2_F^–^ and CF_3_^–^ are not included in their respective groups for the fit and *R* ^2^ calculation.

Although the correlation for each group is high, the slope of all data points together has an opposite sign as compared to that of the individual groups (except for the CHXF_2_ trihalo group). This is known as Simpson’s reversal^81^ and usually signals that there is another variable which may be more appropriate to correlate with/plot against. Figure 3 shows the plot of the gas phase acidity vs the isotropic polarizability of the anion. There is a moderate correlation when the species are grouped by the number of halogen substituents. The points on the left-most of each line are mono-, di-, and trifluoromethanes. Based on these trends, there is a corresponding decrease in the gas phase acidity with increasing polarizability of the anion. This decrease is more pronounced in less substituted halomethanes as evidenced by the change in slope from the mono- to the trihalomethanes. Note that a decrease in the gas phase acidity means a more acidic species.

**Figure 3:**
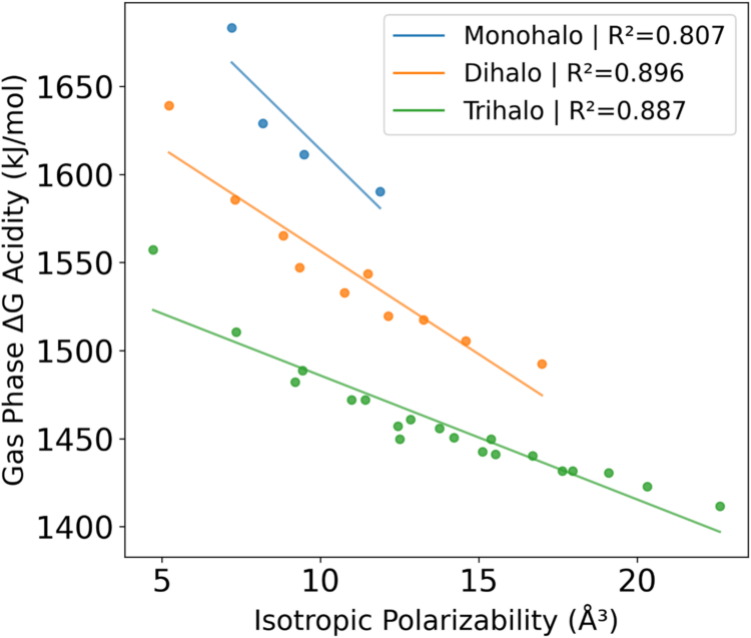
Gas phase acidity versus isotropic polarizability of the resulting anion grouped by the number of halogen substituents. Methane is not shown. Gas phase acidity from FPD with polarizability from MP2/aT.

The gas phase acidity (GA) is composed of the components given in eq 13.

11 $$\mathrm{GA}=\mathrm{BDE}\;\left(C-H\right)-\mathrm{EA}\;\left({\mathrm{CXYZ}}^{•}\right)+\mathrm{IE}\;\left(H\right)$$

where EA is the electron affinity of the halomethane radical and IE is the ionization energy of H. Figure 4 shows the correlation between C–H BDE and gas phase acidity grouped by halogen substitution. Similar to the trends observed with polarizability, the correlation between BDE and gas phase acidity is dependent on the degree of substitution. It is nonexistent for monohalomethanes (*R* ^2^ = 0.003) but becomes chemically significant for the di- and trihalo series. As halogen substitution increases, so does the sensitivity of the C–H BDE to changes in gas phase acidity as seen in the slopes of each line, a trend similar in magnitude, but opposite in direction, to that seen with gas phase acidity versus polarizability.C–H

**Figure 4:**
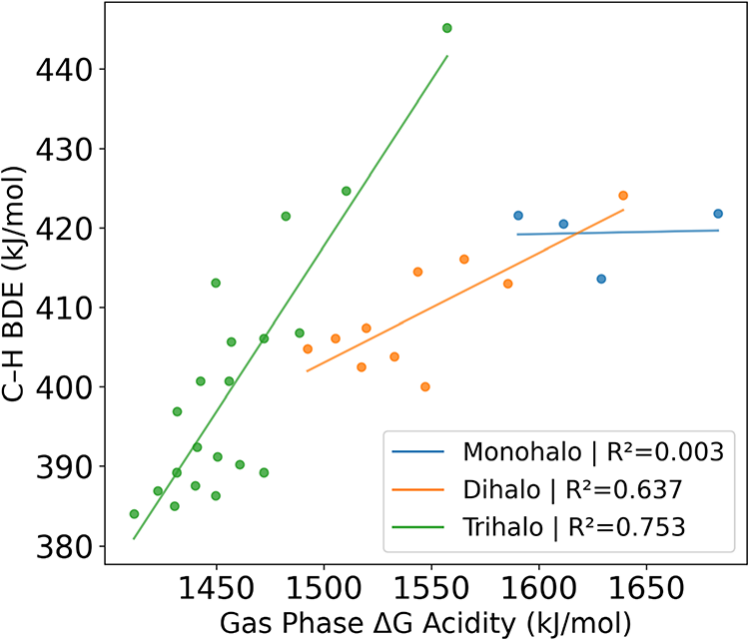
C–H BDE versus gas phase Δ*G* acidity grouped by the number of halogen substituents. Methane is not shown.

Due to the dependence of gas phase acidity on electron affinity shown in eq 12, we have plotted their relationship in Figure 5. Significant correlation is seen with an *R* ^2^ of 0.98. The data shows no evidence of grouping relative to the number of halogen substitutions.

**Figure 5:**
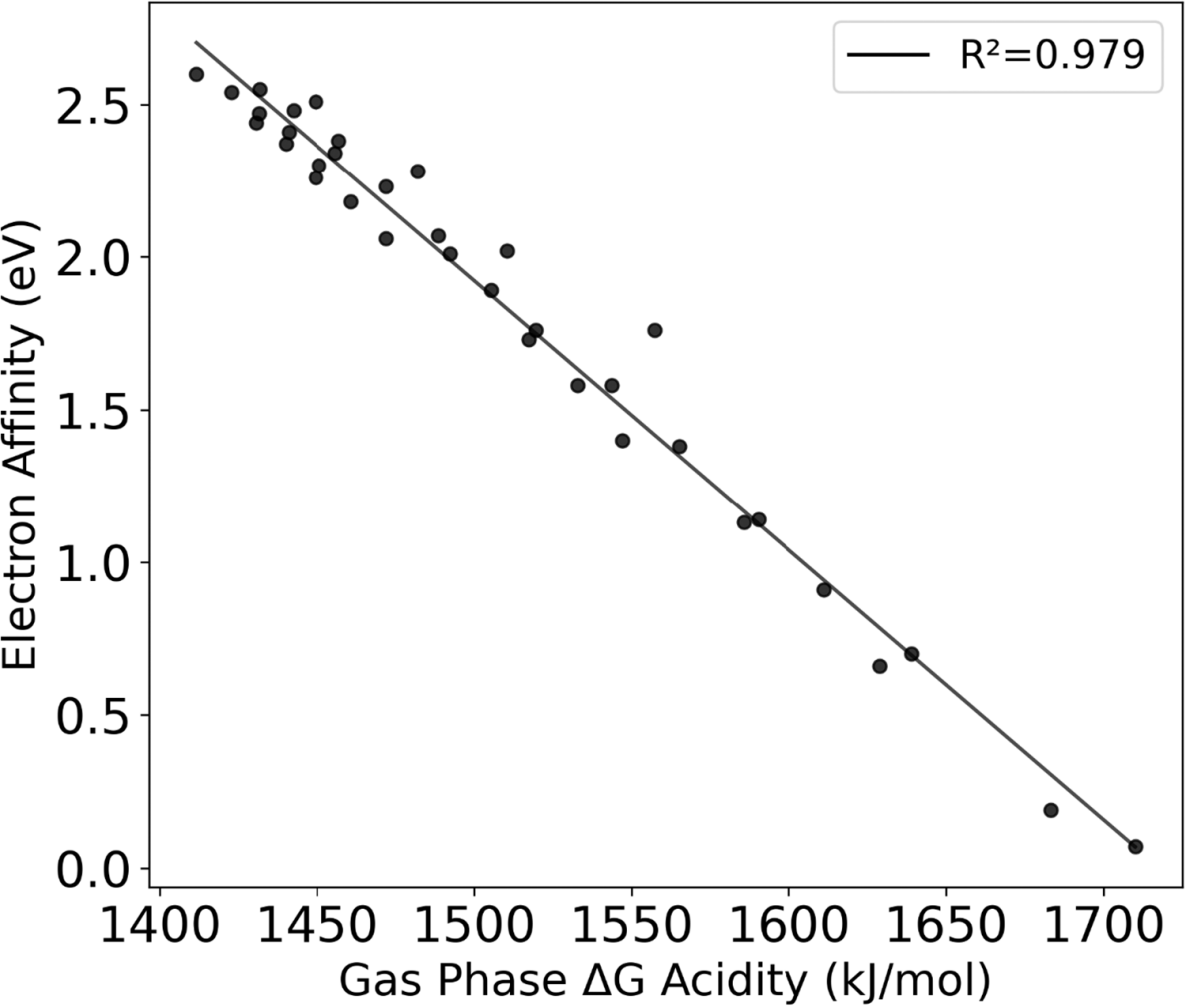
Electron affinity versus gas phase Δ*G* acidity.

### Aqueous Acidity and p*K*_a_

The predicted aqueous acidities and p*K*_a_ values calculated using eq 1 are given in Table 5. We predict a general decrease in aqueous acidity (less positive and lower p*K*_a_) with increasing halogen substitution and halogen size. Slight deviations in this trend are observed when using the SMD solvation model. Note that the SMD model includes additional nonelectrostatic contributions which are not present in COSMO. FPD/SMD predicts the highest aqueous acidity values for all except the smallest species.

**Table 5:** Aqueous Acidity (Δ*G*_aq_) in kJ/mol and p*K*_a_ Using Gas Phase FPD Values

species	Δ*G*_aq_	Δ*G*_aq_	p*K*_a_	p*K*_a_	p*K*_a_
	COSMO	SMD	COSMO	SMD	expt
CH_4_	319.1	316.1	55.9	55.4	48 ^1^
CH_3_F	305.8	306.2	53.6	53.6	
CH_2_F_2_	275.8	279.9	48.3	49.0	
CHF_3_	206.7	212.9	36.2	37.3	32 ^2^
CH_3_Cl	274.9	282.2	48.2	49.4	
CH_2_Cl_2_	222.8	235.0	39.0	41.2	
CHCl_3_	165.7	177.1	29.0	31.0	13.6 ^3^
CH_3_Br	266.4	286.6	46.7	50.2	
CH_2_Br_2_	208.9	236.4	36.6	41.4	
CHBr_3_	150.2	175.3	26.3	30.7	11.8 ^4^
CH_3_I	259.2	266.5	45.4	46.7	
CH_2_I_2_	200.8	219.7	35.2	38.5	
CHI_3_	142.8	172.4	25.0	30.2	
CH_2_FCl	245.7	255.7	43.1	44.8	
CH_2_FBr	235.0	255.2	41.2	44.7	
CH_2_FI	227.8	240.5	39.9	42.1	
CH_2_ClBr	215.6	235.5	37.8	41.3	
CH_2_ClI	211.2	225.9	37.0	39.6	
CH_2_BrI	204.8	225.2	35.9	39.5	
CHF_2_Cl	182.4	195.3	32.0	34.2	
CHF_2_Br	163.6	190.1	28.7	33.3	
CHF_2_I	141.2	160.0	24.7	28.0	
CHCl_2_F	174.2	186.2	30.5	32.6	
CHCl_2_Br	160.3	176.4	28.1	30.9	12.9 ^4^
CHCl_2_I	158.6	175.1	27.8	30.7	
CHBr_2_F	156.7	181.0	27.5	31.7	
CHBr_2_Cl	155.2	176.1	27.2	30.8	12.3 ^4^
CHBr_2_I	149.1	171.9	26.1	30.1	
CHI_2_F	151.5	176.8	26.5	31.0	
CHI_2_Cl	151.8	174.6	26.6	30.6	
CHI_2_Br	147.4	172.0	25.8	30.1	
CHBrClF	165.1	183.5	28.9	32.1	
CHIClF	160.8	178.7	28.2	31.3	
CHIBrF	153.0	174.8	26.8	30.6	
CHIBrCl	153.5	173.2	26.9	30.3	

CHF_2_I has a predicted aqueous acidity which is lower than would be expected from simply following the trend of halogen substitution and size with one of the most favorable aqueous acidities across all computational models. This suggests that there are additional factors other than polarizability from the halogen substituents size affecting the aqueous acidity. This point will be elaborated on further in the discussion of our predicted p*K*_a_ values. Solvent effects for FPD were obtained by applying the Δ*G* solvation from MP2/aT geometries as discussed in the computational methods section.

There is very little experimental data on the aqueous p*K*_a_s of the hydrogen-containing halomethanes for us to compare our calculated values with (Table 5). The experimental p*K*_a_s available from kinetic data shows significant deviation from our calculated values. The Bunnett-Olsen method^82^ relies on a linear correlation between the activity coefficient term for the acid of hydrogen halomethane and that of the indicator acid used for comparison. Scharlin^3^ notes that the measurements with trichloromethane show large deviations from the ideals of the acidity function concept and notes that the activity coefficient had to be assumed. These same assumptions were made for the determination of the p*K*_a_ values of CHBr_3_, CHCl_2_Br, and CHBr_2_Cl; thus these p*K*_a_ values may not be correct. Additional experimental problems that could lead to inaccurate determination of p*K*_a_ are: (1) Nucleophilic attack of hydroxide which is commonly used. This is particularly true with molecules containing good leaving groups like iodine and bromine. (2) A low degree of ionization due to poor solvation leads to a difficult measurement. (3) There is a modified solvation shell due to the high concentration of base relative to the halomethane.

We predict a general decrease in p*K*_a_ (more acidic) with increasing halogen substitution and halogen size. Methane is predicted to have the highest p*K*_a_ across all methods. CH_3_F is predicted to have the highest p*K*_a_ of hydrogen-containing halomethanes across all methods. The third highest p*K*_a_s (CH_3_X species where X = Cl, Br, I) are ∼5 p*K*_a_ units lower. This highlights CH_3_F’s very low reactivity even among low acidity species.

A moderate correlation is observed between the aqueous p*K*_a_ and the solvation contribution of the anion. A stepwise lowering of p*K*_a_ is observed with increasing halogen substitution (Figure 6). This is due to the increase in electronegativity and polarizability compared to substituted hydrogen. These increases effectively stabilize the ion in the gas phase resulting in a less negative solvation energy.

**Figure 6:**
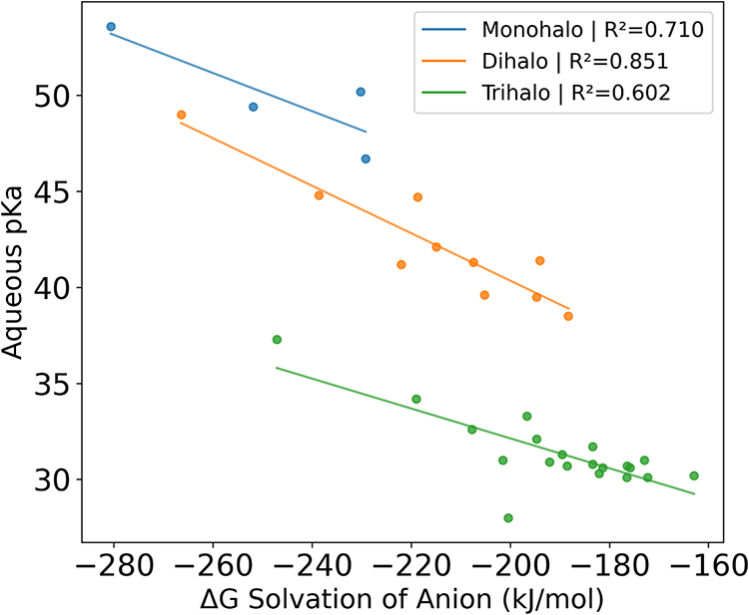
Aqueous p*K*_a_ versus MP2/aT/SMD solvation energy of the anion in water. Methane is not shown.

The correlation of the aqueous p*K*_a_ with the gas phase acidity is given in Figure 7. The good correlation is due to the fact that when the gas phase Δ*G* acidity is higher, i.e., a less stabilized anion in the gas phase, the corresponding Δ*G*_solv_ of the respective anion will be more negative (more stabilized). Due to the correlation between the p*K*_a_ and gas phase acidity, the correlations shown and discussed for gas phase acidity above are applicable to the aqueous p*K*_a_ as well.

**Figure 7:**
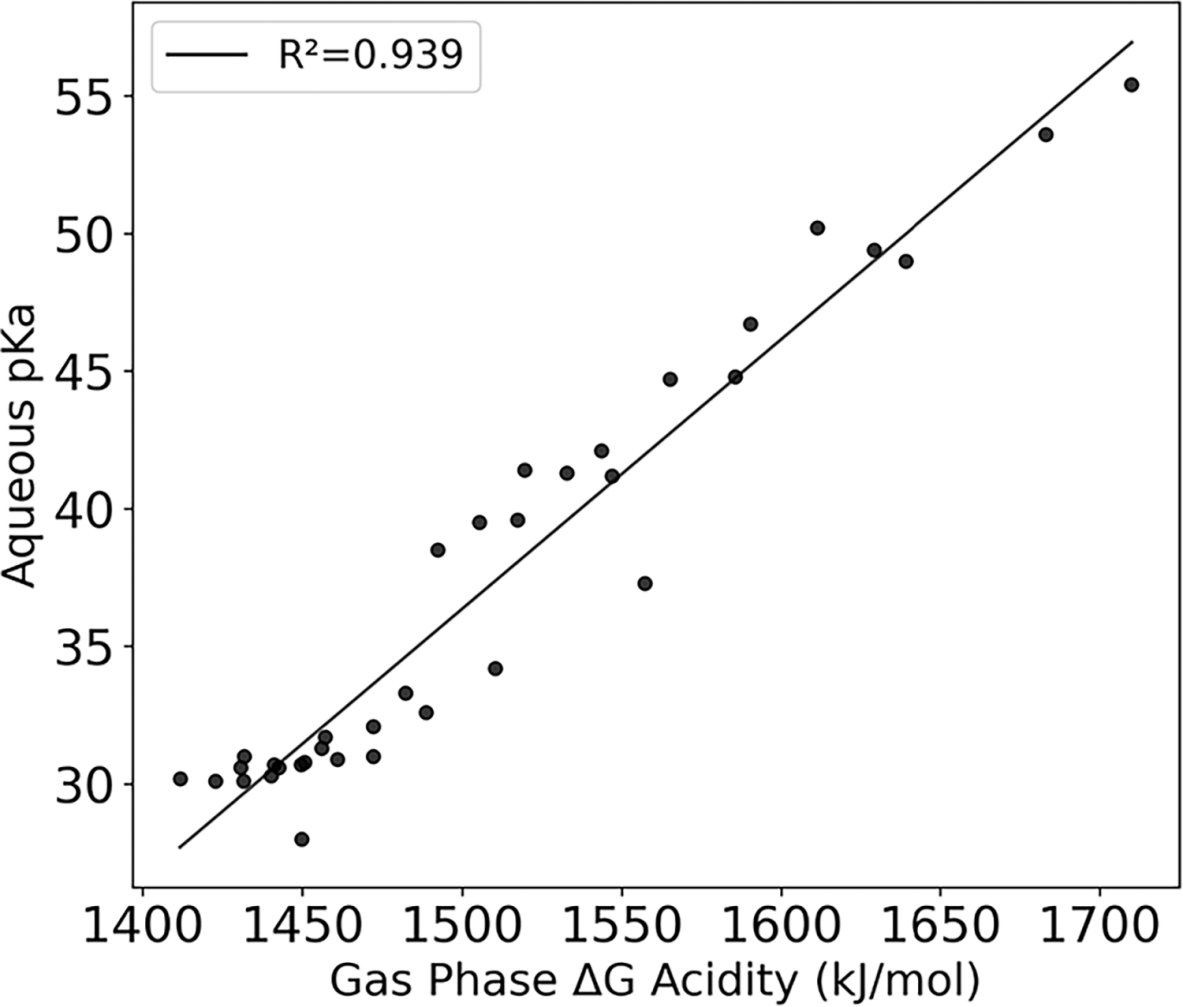
Aqueous p*K*_a_ versus gas phase Δ*G* acidity.

The most acidic species is predicted to be CHF_2_I with a p*K*_a_ of 28.0 using FPD/SMD. As noted above, this does not follow the stated trend of a decrease in p*K*_a_ with increasing halogen size and substitution. For example, CHI_3_, which is expected to have the lowest p*K*_a_ based on the trend, is predicted to have a p*K*_a_ of 30.2 at the same level. When analyzing the structure of CF_2_I^–^, it was noticed that the C–I bond length was particularly long. Natural population analysis (NPA) calculations using Gaussian16 were performed on all four CF_2_X^–^ species using MP2/aT with MP2/aT geometries to investigate further. Table 6 summarizes these calculations as well as bond distances associated with these anions. The iodine atom is seen to have a significant portion of the negative charge in CF_2_I^–^. Table 6 also shows the bond distance change when comparing equivalent radical and anion species. A 0.64 Å increase in C–I bond distance was calculated. These effects are not seen for species like CF_3_^–^, CF_2_Cl^–^, or CF_2_Br^–^. This unique elongation of the C–I bond length in CF_2_I^–^ resembles a CF_2_ carbene interacting with an iodide anion and results in the lower-than-expected p*K*_a_.

**Table 6:** C–F Bond Lengths (*r* in Å) and NPA Charges (*q* in electrons) for CF_2_X^–^ Anions in the Gas Phase

species (CF_2_X^–^)	*q* (C)	*q* (F)	*q* (X)	*r* (C–F)	*r* (C–X)	*r* (C–X) radical	Δ*r* (anion-neutral)
CF_3_^–^	0.59	–0.53	–0.53	1.427	1.427	1.317	0.11
CF_2_Cl^–^	0.53	–0.50	–0.53	1.388	2.057	1.723	0.33
CF_2_Br^–^	0.59	–0.49	–0.61	1.374	2.297	1.881	0.43
CF_2_I^–^	0.72	–0.47	–0.78	1.346	2.738	2.101	0.64

### Nonaqueous p*K*_a_

In addition to aqueous p*K*_a_ values, we calculated the p*K*_a_ in dimethyl sulfoxide (DMSO), acetonitrile (MeCN), and tetrahydrofuran (THF) (Table 7). A comparison of these values reveals that the solution-phase acidity trends do not follow the solvent dielectric constant (Figure 8).

**Figure 8:**
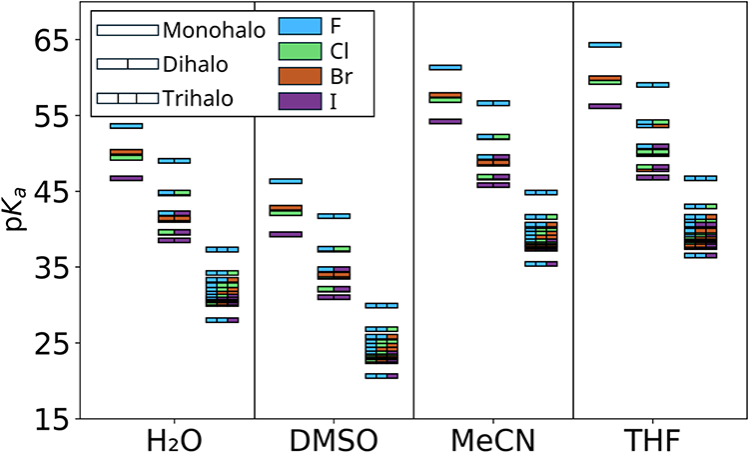
Comparison of p*K*_a_ trends between different solvents.

**Table 7:** p*K*_a_ Values in Different Solvents Calculated Using FPD/SMD

species	water	DMSO	MeCN	THF
CH_4_	55.4	48.1	63.1	66.7
CH_3_F	53.6	46.3	61.3	64.3
CH_2_F_2_	49.0	41.7	56.6	59.0
CHF_3_	37.3	29.9	44.8	46.7
CH_3_Cl	49.4	42.1	57.0	59.4
CH_2_Cl_2_	41.2	33.7	48.6	50.2
CHCl_3_	31.0	23.6	38.4	39.6
CH_3_Br	50.2	42.8	57.7	59.9
CH_2_Br_2_	41.4	34.0	48.8	49.9
CHBr_3_	30.7	23.2	38.0	38.7
CH_3_I	46.7	39.3	54.2	56.2
CH_2_I_2_	38.5	31.0	45.8	46.8
CHI_3_	30.2	22.6	37.4	37.6
CH_2_FCl	44.8	37.4	52.2	54.1
CH_2_FBr	44.7	37.3	52.1	53.7
CH_2_FI	42.1	34.7	49.5	50.9
CH_2_ClBr	41.3	33.8	48.6	50.0
CH_2_ClI	39.6	32.1	46.9	48.2
CH_2_BrI	39.5	32.0	46.8	47.9
CHF_2_Cl	34.2	26.8	41.6	43.0
CHF_2_Br	33.3	25.8	40.6	41.6
CHF_2_I	28.0	20.6	35.4	36.5
CHCl_2_F	32.6	25.2	40.0	41.2
CHCl_2_Br	30.9	23.4	38.2	39.3
CHCl_2_I	30.7	23.2	38.0	38.8
CHBr_2_F	31.7	24.2	39.0	39.8
CHBr_2_Cl	30.8	23.4	38.2	39.0
CHBr_2_I	30.1	22.6	37.4	37.9
CHI_2_F	31.0	25.7	40.4	40.6
CHI_2_Cl	30.6	23.1	37.8	38.3
CHI_2_Br	30.1	22.6	37.4	37.7
CHBrClF	32.1	24.7	39.5	40.5
CHIClF	31.3	23.8	38.6	39.3
CHIBrF	30.6	23.1	37.9	38.5
CHIBrCl	30.3	22.8	37.6	38.3

Instead, the acidity is governed primarily by the solvent’s proton affinity (basicity). We observe that the halomethanes are consistently more acidic in DMSO than in water (by approximately 6 p*K*_a_ units). This increased acidity arises because DMSO stabilizes the proton more effectively than water (Δ*G*_solv_ (H^+^) in DMSO is more negative by ∼45 kJ/mol), while the solvation free energies of the large, charge-delocalized halomethyl anions remain comparable (average absolute difference ≈ 5.3 kJ/mol) between the two solvents.

In contrast, the p*K*_a_ values in MeCN and THF are significantly higher. This is attributed to the weaker proton stabilization in MeCN and the reduced anion solvation in the low-dielectric THF medium (average anion solvation ≈ 22 kJ/mol higher [less negative] in THF compared to other solvents). These results highlight that for carbon acids of this type, specific solvent–proton interactions dominate the thermodynamic profile.

## Experimental Results and Discussion

### Generation of (Mixed) Trihalomethyl Anions

There is a good (qualitative) correlation between our calculated p*K*_a_ values and the strength of bases employed in literature. The acidifying effect of halogens within the series of haloforms is illustrative. For example, trihalomethyl anions could be successfully generated by the treatment with nonorganometallic bases such as KOH^83^ or DBU^84^ (CHCl_3_), KOtBu^85^ (CHBr_3_), and KOH^86^ (CHI_3_). As a consequence, the requirement for stronger bases (e.g., LiHMDS)^87^ could be limited to instances in which ensuring proper nucleophilicity of the anion is critical for the transformation or if the recipient electrophile poses chemoselectivity issues.^88^ On the other hand, little information (with MeLi, p*K*_a_ = 48)^89^ is available on the deprotonation of CHF_3_,^90^ in part due to technical difficulties in the manipulation of this gaseous species and to the extremely high lability of the putative trifluoromethyl anion.^91,92^

Although the series of mixed halo-*difluoro*methanes (CHF_2_Cl, CHF_2_Br and CHF_2_I) have not been used in proton transfer processes, other mixed trihalomethanes have been. For example, Schlosser prepared LiCCl_2_F^93^ starting from CHCl_2_F (p*K*_a_ = 32.6) and *n*-BuLi (p*K*_a_ = 50).^88^ However, the need for such a hard base was not found to be mandatory as documented by the successful use of NaOH with this compound^94^ or analogues such as CHBr_2_F^95,96^ (p*K*_a_ = 31.7), CHBr_2_Cl^84^ (p*K*_a_ = 30.8), CHBr_2_I^97^ (p*K*_a_ = 30.1), CHI_2_Br^96^ (p*K*_a_ = 30.1), CHI_2_F^95^ (p*K*_a_ = 31.0) and CHI_2_Cl^98^ (p*K*_a_ = 30.6).

Because of the lack of precedents on the application of CHBrCl_2_ as an anion precursor, we evaluated different bases for generating the corresponding CBrCl_2_^–^ nucleophile. To this end, the facile amidation of (sufficiently electrophilic) isocyanates^99^ was selected as the model reaction as shown in Scheme 1. As expected, not only were lithium amides (LDA and LTMP) able to perform the transformation (under Barbier-type conditions),^100^ but the weaker DBU (p*K*_a_ = 24 in MeCN)^101^ and KOH also performed the transformation. Although the efficiency of the process with the latter two was not optimal, presumably also due to the competitive attack of the hydroxide to the isocyanate, their capability to realize the deprotonation was confirmed. We can therefore conclude that (mixed) trihalomethanes featuring a calculated p*K*_a_ in the range of 28 to 34 undergo productive deprotonation even with nonorganometallic bases.

**Scheme 1:**
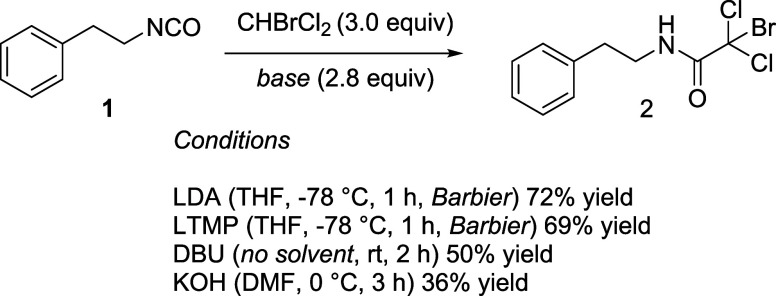
Generation of CBrCl_2_^–^ Anion with Different Bases

### Generation of (Mixed) Dihalomethyl Anions

The significantly lower acidity (higher p*K*_a_) of the dihalomethanes is reflected on the nature of the bases amenable for the effective proton removal. In this sense, metal amides appear to be the reagents of choice regardless of the intercepting electrophile for forming dihalomethyl anions.^102^

(Mixed) dihalomethanes with values of p*K*_a_ in the range 38 to 41 (CH_2_I_2_, CH_2_BrI, CH_2_ClI, CH_2_Cl_2_, CH_2_Br_2_, CH_2_ClBr) can be deprotonated not only with harsh basic lithium amides e.g., LTMP,^101,103^ LDA,^102^ LHMDS^104,105^ with p*K*_a_s between 30 to 37,^88^ but also with less harsh analogues such as NaHMDS^103,104^ (CH_2_I_2_, CH_2_ClI, CH_2_Br_2_ with p*K*_a_ = 29.5) or, as shown more recently, with the Knochel-Hauser magnesium amide TMPMgCl-LiCl^106^ whose p*K*_a_ is unknown.^107^

An interesting effect is observed in the case of two commercially available fluorohalomethyl anion precursors CH_2_FI and CH_2_FBr.^108^ The increased respective p*K*_a_ values of 42.1 and 44.7 are diagnostic of requiring lithium amides of compatible strength as only LTMP,^109^ LDA^110,111^ and LiN­(i-Pr)­Cy^108^ have so far been used successfully. It is worth noting that LHDMS (p*K*_a_ = 30)^88^ could not be employed for deprotonating these mixed fluorohalomethanes. No data is available for CH_2_FCl and CH_2_F_2_ likely due to their gaseous physical state which hampers extensive synthetic applications. In conclusion, as one could expect, the abstraction of protons from dihalomethanes is a more difficult operation requiring strong metal amides bases.

## Conclusions

In this work we have calculated thermochemical data associated with the exhaustive list of hydrogen-containing halomethanes as well as solvent effects on these species. We have shown excellent agreement to the available high accuracy experimental data (within 5 kJ/mol for nearly all gas phase values) with FPD and our modified G3­(MP2) which incorporates iodine and extends the ability of low-cost, high-accuracy calculations to the entire set of halomethanes without the need to reparametrize the HLC corrections. For species without experimental data, we report a median absolute difference of < 6 kJ/mol between our FPD and modified G3­(MP2) results. Our predicted p*K*_a_ values show that the reported experimental values in the literature systematically overestimate the aqueous acidity by ∼ 15 p*K*_a_ units for most of these species likely due to assumptions made in their kinetic acidity functions. Proton affinity, electron affinity, and aqueous acidity scale consistently with halogen substitution and size, which are associated with polarizability. However, the trend for C–H bond energy is distinct. The correlation with polarizability is absent in monohalo species but becomes prominent in di- and trihalo species, suggesting that polarizability-driven stabilization requires a threshold of substitution. This indicates that while the thermochemical properties of these species generally correlate with component polarizability, the sensitivity of the C–H bond to these effects is dependent on the degree of substitution.C–H CH_2_F_2_ differs from our calculated value by nearly 40 kJ/mol for Δ*G* acidity and a revision of the experimental value is needed. We predict that CH_3_F has the least acidic p*K*_a_ of the hydrogen-containing halomethanes with a value of 53.6 and CHF_2_I has the most acidic p*K*_a_ with a value of 28.0 at FPD/SMD primarily due to its anion resembling a CF_2_ carbene and an iodine anion. Across water, DMSO, MeCN, and THF, we predict that acidity trends are driven by the solvent’s proton affinity (basicity) rather than the dielectric constant, resulting in higher acidities in DMSO and water compared to MeCN and THF. Consistent with the halogen substitution trends, literature base choices for anion generation can now be rationalized. (Mixed) trihalomethanes with calculated p*K*_a_ values between 28 and 34 are deprotonated by nonorganometallic bases (e.g., KOH, DBU, KOtBu), whereas the less acidic dihalomethanes (p*K*_a_ ≳ 38) generally require strong metal amides. Fluorodihalomethanes, at the upper end of the p*K*_a_ range (p*K*_a_ of 42 to 49), require the most basic lithium amides (e.g., LTMP, LDA, LiN­(i-Pr)­Cy) where LHMDS is inadequate. The CHBrCl_2_ case study (Scheme 1) further validates these predictions. Lithium amides allow clean deprotonation and interception, whereas weaker bases (DBU, KOH) still generate CBrCl_2_^–^, albeit with reduced efficiency, due to competitive hydroxide addition. The calculated p*K*_a_ values thus provide practical guidance for base selection and chemoselectivity for future syntheses.

## Supplementary Material



## Notes

Total dissociation energies, thermochemical components for FPD, proton affinities, solvation energies, absolute p*K*_a_s, total energies in a.u., and optimized coordinates in Å; additional experimental details (PDF)

## Notes

This work is dedicated to the memory of Professor David A. Dixon, whose untimely passing during the completion of this publication represents an immeasurable loss to the field of computational chemistry and to all who had the privilege of knowing him. Professor Dixon was a pioneering scientist whose contributions to theoretical and computational chemistry have shaped our understanding of chemical systems. Though he is no longer here to see its completion, his influence remains in every page of this publication. It is our hope that this work reflects the standards he set and honors the legacy of a remarkable scientist, mentor, and teacher. D.A.D. (1949–2026)
