# Supplementary material for: Correlated Molecular Orbital Theory Predictions of Hydrogen-Containing Halomethane Thermochemistry: Heats of Formation, C–H Bond Dissociation Energies, and pK a Values
Source: J Phys Chem A. 2026 Feb 20;130(9):1790–803. doi: 10.1021/acs.jpca.5c08066 (PMC12969366; doi:10.1021/acs.jpca.5c08066)
Supplement: Supplementary file 1 [file jp5c08066_si_001.pdf]

Correlated Molecular Orbital Theory Predictions of Hydrogen-Containing Halomethane Thermochemistry: Heats of Formation, C–H Bond Dissociation Energies, and pK_a Values.

Thomas Dalton Andress,[a] Cole Seely,[a] Margherita Miele,[b] Laura Castoldi,[c] Vittorio Pace[d,e,*] and David A. Dixon[a,*]

[a] Department of Chemistry and Biochemistry, The University of Alabama, Tuscaloosa, Alabama 35487, United States

[b] University of Turin, Department of Chemistry, Via Giuria 7, Turin, 10125, Italy

[c] University of Milan, Department of Pharmaceutical Sciences, General and Organic Chemistry Section "A. Marchesini", Via Venezian 21, Milan, 20133 Italy

[d] University of Rome "La Sapienza", Department of Chemistry, P.le A. Moro 5, Rome, 00185, Italy

[e] University of Vienna, Department of Pharmaceutical Sciences, Division of Pharmaceutical Chemistry, Josef-Holaubek-Platz 2, Vienna, 1090, Austria

* **Email**: D. A. Dixon, dadixon@ua.edu, Vittorio Pace, vittorio.pace@uniroma1.it

Table S1. Total Dissociation Energies of Neutral Species at 0 K in kJ/mol.

Species	B3LYP/aT	MP2/aT	G3MP2	TQ FPD	Q5 FPD	TQ5 FPD	Expt. ATcT
CH_4	1642.6	1598.2	1642.0	1642.5	1641.6	1640.7	1642.1 ± 0.06
CH_3F	1661.0	1645.6	1664.8	1665.2	1663.4	1662.4	1664.2 ± 0.24
CH_2F_2	1729.2	1748.5	1741.7	1742.8	1740.0	1738.8	1741.3 ± 0.35
CHF_3	1823.8	1882.1	1849.0	1851.6	1847.5	1846.1	1848.5 ± 0.4
CH_3Cl	1537.4	1522.0	1553.1	1553.6	1554.6	1553.1	1553.8 ± 0.17
CH_2Cl_2	1433.0	1452.3	1471.2	1468.7	1472.1	1470.0	1469.5 ± 0.33
$CHCl_3$	1319.3	1379.9	1387.2	1378.9	1385.1	1382.2	1380.8 ± 0.39
CH_3Br	1480.8	1474.4	1499.1	1497.5	1497.1	1496.0	1497.9 ± 0.20
CH_2Br_2	1315.0	1354.6	1357.4	1351.5	1352.0	1350.5	1352.3 ± 0.6
$CHBr_3$	1138.5	1233.1	1211.5	1199.6	1201.0	1199.2	1200.2 ± 0.7
CH_3I	1421.5	1417.8	1440.8	1439.8	1439.4	1438.4	1442.1 ± 0.16
CH_2I_2	1191.1	1238.3	1239.0	1233.0	1233.2	1232.0	1234.8 ± 0.8
CHI_3	948.8	1057.9	1035.8	1027.5	1028.5	1026.9	1031.6 ± 1.6
CH_2FCl	1575.7	1591.4	1597.6	1597.6	1597.8	1596.1	1596.8 ± 0.9
CH_2FBr	1513.8	1537.3	1536.5	1534.6	1533.5	1532.1	1536.0 ± 5
CH_2FI	1447.1	1470.9	1469.9	1467.8	1466.6	1465.4	
CH_2ClBr	1373.9	1402.9	1414.1	1409.8	1411.8	1410.0	1409.2 ± 1.3
CH_2ClI	1310.9	1342.7	1353.4	1348.5	1350.5	1348.8	
CH_2BrI	1252.6	1295.6	1300.0	1291.4	1291.8	1290.4	
CHF_2Cl	1646.3	1698.2	1679.0	1679.1	1678.3	1676.5	1677.1 ± 1.0
CHF_2Br	1581.0	1639.8	1613.2	1611.5	1609.2	1607.8	1611.3 ± 0.5
CHF_2I	1510.3	1567.5	1541.7	1539.2	1537.0	1535.6	
$CHCl_2F$	1477.9	1530.7	1525.2	1521.4	1524.0	1521.7	1521.5 ± 0.9
$CHCl_2Br$	1258.9	1329.9	1328.3	1318.6	1323.2	1320.7	1319.4 ± 1.2
$CHCl_2I$	1194.5	1268.4	1267.5	1256.0	1260.7	1258.3	
$CHBr_2F$	1352.2	1423.1	1400.5	1393.7	1393.4	1391.8	1400.2 ± 5
$CHBr_2Cl$	1198.7	1281.0	1269.8	1258.8	1261.9	1259.7	1260.9 ± 3.5
$CHBr_2I$	1074.8	1173.2	1156.8	1138.9	1140.2	1138.5	
CHI_2F	1218.1	1292.3	1270.4	1261.9	1261.3	1259.9	
CHI_2Cl	1070.9	1161.0	1149.9	1136.6	1139.6	1137.6	
CHI_2Br	1011.6	1114.8	1095.6	1079.4	1080.5	1078.9	
CHBrClF	1415.0	1476.4	1462.7	1457.3	1458.5	1456.6	
CHIClF	1347.4	1409.5	1396.5	1389.9	1391.0	1389.2	
CHIBrF	1284.9	1357.0	1337.3	1327.2	1326.8	1325.3	
CHIBrCl	1134.6	1220.3	1212.0	1197.1	1200.1	1198.1	

Table S2. Total Dissociation Energies of Radical Species at 0 K in kJ/mol.

Species	B3LYP/aT	MP2/aT	G3MP2	TQ FPD	Q5 FPD	TQ5 FPD	Expt. ATcT
$CH_3^•$	1217.6	1180.3	1213.7	1210.8	1209.9	1209.2	1209.6 ± 0.06
$CH_2F^•$	1254.6	1240.5	1248.5	1247.8	1246.1	1245.1	
$CHF_2^•$	1322.4	1339.5	1323.2	1322.7	1320.1	1318.9	
$CF_3^•$	1397.2	1451.5	1409.0	1410.0	1406.4	1405.1	1408.3 ± 0.4
$CH_2Cl^•$	1137.8	1122.7	1144.2	1144.2	1146.4	1144.8	1144.6 ± 0.9
$CHCl_2^•$	1048.1	1061.7	1075.4	1071.4	1076.4	1074.1	1073.8 ± 0.9
$CCl_3^•$	947.0	997.3	1001.8	992.2	1000.0	996.9	996.5 ± 0.8
$CH_2Br^•$	1076.6	1068.5	1085.1	1081.9	1081.8	1080.7	
$CHBr_2^•$	925.6	957.3	957.4	948.3	948.9	947.5	
$CBr_3^•$	767.0	847.2	827.0	811.4	812.8	811.0	
$CH_2I^•$	1014.9	1010.2	1025.7	1023.8	1023.4	1022.5	
$CHI_2^•$	803.6	842.5	842.2	832.7	833.0	831.8	
$CI_3^•$	584.7	679.2	660.6	641.5	642.6	641.0	
$CHFCl^•$	1178.7	1190.6	1189.2	1187.8	1189.0	1187.2	
$CHFBr^•$	1115.1	1133.6	1126.4	1122.5	1121.5	1120.2	
$CHFI^•$	1049.7	1068.4	1061.6	1057.4	1056.3	1055.2	
$CHClBr^•$	986.6	1008.9	1016.1	1009.5	1012.4	1010.5	
$CHClI^•$	924.6	949.7	957.7	949.6	952.4	950.7	
$CHBrI^•$	864.1	899.1	902.0	889.7	890.1	888.8	
$CF_2Cl^•$	1240.1	1285.8	1259.2	1257.8	1257.8	1255.9	
$CF_2Br^•$	1179.7	1230.3	1197.5	1193.9	1191.9	1190.5	
$CF_2I^•$	1118.0	1166.2	1134.7	1129.9	1128.0	1126.7	
$CCl_2F^•$	1088.9	1133.2	1122.7	1117.5	1121.3	1118.8	
$CCl_2Br^•$	887.0	946.5	943.3	931.5	937.3	934.6	
$CCl_2I^•$	826.1	888.6	887.5	872.7	878.6	876.0	
$CBr_2F^•$	967.3	1026.3	1001.3	992.1	991.9	990.3	
$CBr_2Cl^•$	827.0	896.4	885.1	871.3	874.9	872.6	
$CBr_2I^•$	706.2	790.3	776.3	753.9	755.2	753.5	
$CI_2F^•$	842.4	903.3	880.3	868.9	868.5	867.2	
$CI_2Cl^•$	705.2	782.4	772.8	755.2	758.9	756.8	
$CI_2Br^•$	645.4	734.3	718.0	696.8	698.0	696.3	
$CBrClF^•$	1028.1	1079.5	1062.0	1054.6	1056.5	1054.5	
$CIClF^•$	966.1	1017.8	1001.9	992.7	994.5	992.6	
$CIBrF^•$	905.1	964.7	943.4	930.4	930.2	928.7	
$CIBrCl^•$	766.1	839.1	831.9	813.1	816.7	814.6	

Table S3. Total Dissociation Energies of Anion Species at 0 K in kJ/mol.

Species	B3LYP/aT	MP2/aT	G3MP2	TQ FPD	Q5 FPD	TQ5 FPD	Expt. ATcT
CH_3^-	1228.6	1183.4	1214.3	1216.4	1216.2	1215.2	1218.3 ± 0.24
CH_2F^-	1279.6	1253.4	1268.0	1266.0	1264.5	1263.5	
CHF_2^-	1396.9	1400.3	1393.3	1389.5	1387.4	1386.1	
CF_3^-	1573.3	1614.5	1580.2	1579.1	1576.5	1575.0	1580.9 ± 1.8
CH_2Cl^-	1208.1	1182.0	1216.2	1209.0	1209.9	1208.3	1206.8 ± 2.0
$CHCl_2^-$	1190.6	1192.2	1219.9	1208.2	1211.2	1208.9	1207.4 ± 2.0
CCl_3^-	1154.7	1192.7	1212.2	1193.1	1198.6	1195.7	1193.4 ± 2.0
CH_2Br^-	1167.7	1148.0	1179.2	1170.6	1170.0	1168.7	
$CHBr_2^-$	1097.4	1114.6	1132.5	1118.4	1118.4	1116.7	
CBr_3^-	1001.7	1067.3	1066.3	1044.4	1045.3	1043.1	
CH_2I^-	1127.0	1112.0	1139.2	1134.0	1133.4	1132.2	
CHI_2^-	997.3	1026.0	1038.9	1027.2	1027.0	1025.5	
CI_3^-	834.1	921.8	915.4	892.5	893.1	891.2	
$CHFCl^-$	1298.0	1294.3	1307.3	1298.3	1298.3	1296.6	
$CHFBr^-$	1255.6	1255.1	1264.7	1255.6	1254.3	1252.9	
$CHFI^-$	1209.3	1208.3	1214.5	1210.2	1208.9	1207.5	
$CHClBr^-$	1144.6	1153.3	1176.4	1163.5	1165.0	1163.1	
$CHClI^-$	1095.7	1108.0	1129.9	1117.6	1119.2	1117.3	
$CHBrI^-$	1047.6	1069.7	1088.2	1072.5	1072.5	1070.8	
CF_2Cl^-	1449.9	1473.2	1457.8	1454.1	1453.1	1451.3	
CF_2Br^-	1409.8	1433.3	1419.4	1413.6	1411.5	1409.8	
CF_2I^-	1364.5	1384.7	1374.3	1372.1	1369.7	1368.0	
CCl_2F^-	1301.9	1327.0	1332.1	1318.8	1320.7	1318.4	
CCl_2Br^-	1104.9	1150.6	1164.0	1143.8	1148.0	1145.2	
CCl_2I^-	1051.4	1099.7	1114.2	1092.4	1096.6	1093.9	
CBr_2F^-	1206.1	1241.0	1236.6	1222.3	1221.5	1219.7	
CBr_2Cl^-	1053.8	1108.8	1115.4	1094.2	1096.8	1094.4	
CBr_2I^-	946.3	1017.5	1021.0	993.1	993.9	991.8	
CI_2F^-	1097.1	1135.7	1130.1	1115.7	1114.6	1112.9	
CI_2Cl^-	943.9	1009.2	1014.4	991.9	994.5	992.1	
CI_2Br^-	890.3	969.0	967.9	942.4	943.1	941.1	
$CBrClF^-$	1255.0	1284.0	1283.7	1271.1	1271.7	1269.6	
$CIClF^-$	1203.6	1231.2	1230.9	1219.7	1220.3	1218.3	
$CIBrF^-$	1152.6	1188.1	1185.5	1170.0	1169.2	1167.4	
$CIBrCl^-$	999.3	1058.4	1067.9	1042.8	1045.4	1043.0	

ΔZPE, ΔE_{SO}, ΔE_{SR}, ΔE_{CV}, $\Delta H_{0\text{ to }298}$, $\Delta E_{H0\text{ to }G298}$ in Tables S4, S5, and S6 were calculated using MP2/aug-cc-p(wC)VTZ(-PP,-DK). Zero-point energies were scaled by 0.95 relative to the calculated value.

Table S4. FPD Thermochemical Components for Neutral Species in kJ/mol.

Species	ΔE_{CBS}(Q5)	ΔE_{CBS}(TQ5)	ΔZPE	ΔE_{SO}	ΔE_{SR}	ΔE_{CV}	$\Delta H_{0\ to\ 298}$	$\Delta E_{H0\ to\ G298}$
CH_4	1753.5	1752.5	-115.4	-0.3	-0.8	4.7	10.0	-45.4
CH_3F	1764.5	1763.4	-102.0	-2.0	-1.6	4.5	10.1	-56.2
CH_2F_2	1827.1	1825.9	-85.6	-3.6	-2.2	4.3	10.7	-64.5
CHF_3	1917.7	1916.3	-66.4	-5.2	-2.9	4.3	11.5	-65.8
CH_3Cl	1653.1	1651.6	-97.9	-3.8	-1.7	5.1	10.4	-59.3
CH_2Cl_2	1552.9	1550.7	-76.7	-7.4	-2.3	5.6	11.7	-70.3
$CHCl_3$	1444.8	1441.9	-52.4	-10.9	-2.7	6.3	14.0	-73.8
CH_3Br	1602.1	1601.0	-96.2	-15.0	-0.6	6.9	10.5	-62.5
CH_2Br_2	1446.0	1444.5	-73.3	-29.7	-0.6	9.5	12.5	-76.4
$CHBr_3$	1280.8	1279.0	-47.5	-44.4	-0.5	12.5	15.7	-82.4
CH_3I	1555.7	1554.6	-94.4	-30.7	-0.5	9.3	10.7	-64.7
CH_2I_2	1350.1	1348.9	-70.2	-61.0	-0.2	14.5	13.1	-80.7
CHI_3	1137.0	1135.5	-43.8	-85.1	0.2	20.1	16.9	-88.8
CH_2FCl	1681.6	1679.9	-80.9	-5.5	-2.3	4.9	11.2	-67.5
CH_2FBr	1623.7	1622.4	-79.2	-16.7	-1.4	7.0	11.5	-70.6
CH_2FI	1568.0	1566.9	-77.5	-32.3	-1.2	9.6	11.8	-72.8
CH_2ClBr	1499.1	1497.3	-74.9	-18.5	-1.5	7.6	12.1	-73.3
CH_2ClI	1449.1	1447.5	-73.4	-34.2	-1.3	10.2	12.4	-75.6
CH_2BrI	1397.2	1395.8	-71.7	-45.4	-0.4	12.1	12.8	-78.5
CHF_2Cl	1744.7	1742.8	-61.4	-7.1	-2.8	4.9	12.3	-71.3
CHF_2Br	1682.0	1680.5	-59.6	-18.3	-2.0	7.1	12.8	-74.4
CHF_2I	1621.1	1619.8	-58.1	-33.9	-1.9	9.7	13.2	-76.6
$CHCl_2F$	1586.9	1584.6	-56.7	-9.0	-2.7	5.5	13.2	-74.0
$CHCl_2Br$	1389.6	1387.1	-50.7	-22.0	-2.0	8.4	14.6	-79.4
$CHCl_2I$	1338.6	1336.2	-49.5	-37.7	-1.8	11.1	14.9	-81.6
$CHBr_2F$	1469.3	1467.7	-53.3	-31.3	-1.2	9.9	14.2	-79.9
$CHBr_2Cl$	1335.0	1332.8	-49.1	-33.2	-1.3	10.5	15.1	-82.3
$CHBr_2I$	1231.6	1229.9	-46.2	-60.0	-0.3	15.1	16.1	-87.3
CHI_2F	1360.2	1358.8	-50.5	-62.6	-0.8	15.1	15.0	-84.3
CHI_2Cl	1235.8	1233.9	-46.6	-64.5	-0.8	15.7	15.9	-86.6
CHI_2Br	1183.6	1182.0	-45.0	-75.7	-0.1	17.6	16.5	-89.4
CHBrClF	1527.9	1525.9	-55.0	-20.2	-2.0	7.7	13.7	-76.9
CHIClF	1471.9	1470.0	-53.6	-35.8	-1.8	10.4	14.1	-79.2
CHIBrF	1414.2	1412.7	-51.9	-47.0	-1.0	12.5	14.6	-82.1
CHIBrCl	1284.8	1282.7	-47.8	-48.9	-1.1	13.1	15.5	-84.5

Table S5. FPD Thermochemical Components for Radical Species in kJ/mol.

Species	ΔE_{CBS}(Q5)	ΔE_{CBS}(TQ5)	ΔZPE	ΔE_{SO}	ΔE_{SR}	ΔE_{CV}	$\Delta H_{0\text{ to }298}$	$\Delta E_{H0\text{ to }G298}$
$CH_3^\bullet$	1283.4	1282.6	-76.4	-0.3	-0.7	4.0	10.6	-49.2
$CH_2F^\bullet$	1309.7	1308.8	-64.4	-2.0	-1.5	4.1	10.4	-59.7
$CHF_2^\bullet$	1371.9	1370.8	-50.2	-3.6	-2.1	4.0	10.6	-65.7
$CF_3^\bullet$	1442.7	1441.3	-32.2	-5.2	-2.7	3.9	11.5	-67.3
$CH_2Cl^\bullet$	1206.0	1204.5	-58.9	-3.8	-1.8	4.8	11.5	-63.6
$CHCl_2^\bullet$	1122.2	1119.8	-41.5	-7.4	-2.4	5.5	11.9	-71.5
$CCl_3^\bullet$	1027.2	1024.1	-19.6	-10.9	-2.8	6.1	14.0	-75.4
$CH_2Br^\bullet$	1149.1	1148.1	-57.9	-15.0	-0.7	6.2	11.4	-66.5
$CHBr_2^\bullet$	1009.3	1007.9	-38.7	-29.7	-0.6	8.6	12.6	-77.6
$CBr_3^\bullet$	861.9	860.1	-15.4	-44.4	-0.6	11.2	15.7	-84.0
$CH_2I^\bullet$	1101.9	1100.9	-55.8	-30.7	-0.3	8.3	12.1	-69.7
$CHI_2^\bullet$	916.8	915.7	-36.1	-61.0	0.1	13.2	13.4	-82.0
$CI_3^\bullet$	727.8	726.3	-12.8	-91.3	0.5	18.4	17.1	-93.4
$CHFCl^\bullet$	1237.7	1236.0	-45.7	-5.5	-2.2	4.7	11.1	-68.6
$CHFBr^\bullet$	1177.4	1176.2	-44.3	-16.7	-1.3	6.3	11.5	-71.7
$CHFI^\bullet$	1123.9	1122.8	-42.9	-32.3	-1.0	8.5	11.8	-74.0
$CHClBr^\bullet$	1065.4	1063.5	-40.1	-18.5	-1.5	7.1	12.2	-74.5
$CHClI^\bullet$	1017.2	1015.4	-38.8	-34.2	-1.2	9.4	12.6	-76.8
$CHBrI^\bullet$	962.2	960.9	-37.4	-45.4	-0.3	10.9	13.0	-79.8
$CF_2Cl^\bullet$	1290.7	1288.8	-27.6	-7.1	-2.6	4.5	12.2	-72.8
$CF_2Br^\bullet$	1232.0	1230.5	-26.1	-18.3	-1.9	6.2	12.7	-75.9
$CF_2I^\bullet$	1180.1	1178.8	-24.9	-33.9	-1.6	8.3	13.2	-78.2
$CCl_2F^\bullet$	1151.2	1148.7	-23.4	-9.0	-2.7	5.2	13.1	-75.5
$CCl_2Br^\bullet$	971.8	969.1	-18.2	-22.0	-2.1	7.8	14.5	-81.0
$CCl_2I^\bullet$	925.1	922.5	-17.3	-37.7	-1.8	10.2	14.9	-83.2
$CBr_2F^\bullet$	1036.2	1034.6	-20.4	-31.3	-1.2	8.7	14.1	-81.4
$CBr_2Cl^\bullet$	916.7	914.5	-16.8	-33.2	-1.3	9.5	15.1	-83.9
$CBr_2I^\bullet$	816.4	814.7	-14.5	-60.0	-0.2	13.6	16.2	-88.9
$CI_2F^\bullet$	937.0	935.7	-18.3	-62.6	-0.7	13.2	15.0	-85.9
$CI_2Cl^\bullet$	825.1	823.0	-15.0	-64.5	-1.0	14.4	16.0	-88.4
$CI_2Br^\bullet$	771.6	770.0	-13.6	-75.7	-0.3	16.0	16.6	-91.2
$CBrClF^\bullet$	1093.6	1091.6	-21.9	-20.2	-2.0	7.0	13.6	-78.5
$CIClF^\bullet$	1043.6	1041.7	-20.8	-35.8	-1.6	9.2	14.0	-80.7
$CIBrF^\bullet$	986.5	985.0	-19.4	-47.0	-0.9	10.9	14.6	-83.7
$CIBrCl^\bullet$	870.6	868.4	-15.9	-48.9	-1.0	11.9	15.5	-86.1

Table S6. FPD Thermochemical Components for Anionic Species in kJ/mol.

Species	ΔE_{CBS}(Q5)	ΔE_{CBS}(TQ5)	ΔZPE	ΔE_{SO}	ΔE_{SR}	ΔE_{CV}	$\Delta H_{0\text{ to }298}$	$\Delta E_{H0\text{ to }G298}$
CH_3^-	1287.1	1286.1	-73.3	-0.3	-0.8	3.6	10.2	-47.5
CH_2F^-	1325.5	1324.5	-60.6	-2.0	-1.5	3.1	10.3	-58.5
CHF_2^-	1434.9	1433.7	-44.5	-3.6	-2.1	2.6	11.1	-64.7
CF_3^-	1606.5	1605.0	-24.5	-5.2	-2.5	2.3	12.2	-66.1
CH_2Cl^-	1270.1	1268.5	-57.8	-3.8	-1.8	3.3	10.6	-61.7
$CHCl_2^-$	1256.2	1254.0	-38.5	-7.4	-2.4	3.2	12.3	-70.8
CCl_3^-	1223.5	1220.6	-14.8	-10.9	-2.4	3.2	15.1	-74.6
CH_2Br^-	1237.8	1236.6	-56.8	-15.0	-0.4	4.5	10.8	-64.9
$CHBr_2^-$	1178.4	1176.8	-36.4	-29.7	-0.2	6.2	13.0	-76.9
CBr_3^-	1093.3	1091.2	-11.9	-44.4	-0.2	8.4	16.7	-83.6
CH_2I^-	1213.8	1212.6	-56.2	-30.7	-0.2	6.6	10.9	-67.1
CHI_2^-	1111.6	1110.0	-34.9	-61.0	0.4	10.9	13.3	-81.1
CI_3^-	978.3	976.3	-10.3	-91.3	1.0	15.5	17.6	-92.8
$CHFCl^-$	1344.9	1343.3	-41.7	-5.5	-2.2	2.8	11.8	-68.0
$CHFBr^-$	1308.5	1307.1	-40.7	-16.7	-1.1	4.2	12.2	-71.2
$CHFI^-$	1275.2	1273.9	-39.9	-32.3	-0.7	6.5	12.4	-73.5
$CHClBr^-$	1217.6	1215.6	-37.5	-18.5	-1.3	4.7	12.6	-73.9
$CHClI^-$	1184.0	1182.0	-36.7	-34.2	-1.0	7.1	12.8	-76.0
$CHBrI^-$	1144.8	1143.2	-35.6	-45.4	0.1	8.6	13.2	-79.0
CF_2Cl^-	1481.1	1479.3	-20.6	-7.1	-2.4	2.2	13.7	-72.7
CF_2Br^-	1447.4	1445.8	-19.8	-18.3	-1.5	3.7	14.3	-76.5
CF_2I^-	1418.9	1417.2	-19.3	-33.9	-1.1	5.1	15.1	-80.8
CCl_2F^-	1347.0	1344.7	-17.5	-9.0	-2.3	2.6	14.4	-75.1
CCl_2Br^-	1180.6	1177.8	-13.9	-22.0	-1.7	5.0	15.6	-80.4
CCl_2I^-	1141.5	1138.8	-13.3	-37.7	-1.3	7.4	15.9	-82.7
CBr_2F^-	1263.3	1261.5	-15.8	-31.3	-0.8	6.1	15.4	-81.4
CBr_2Cl^-	1137.1	1134.7	-12.9	-33.2	-0.9	6.7	16.1	-83.4
CBr_2I^-	1054.3	1052.3	-11.3	-60.0	0.1	10.8	17.0	-88.5
CI_2F^-	1183.2	1181.5	-14.7	-62.6	-0.1	8.8	16.0	-85.9
CI_2Cl^-	1059.4	1057.1	-11.8	-64.5	-0.2	11.5	16.7	-87.8
CI_2Br^-	1015.9	1013.9	-10.8	-75.7	0.5	13.2	17.3	-90.7
$CBrClF^-$	1305.8	1303.7	-16.6	-20.2	-1.5	4.3	14.9	-78.3
$CIClF^-$	1266.7	1264.7	-16.1	-35.8	-1.2	6.6	15.2	-80.7
$CIBrF^-$	1223.4	1221.6	-15.2	-47.0	-0.4	8.4	15.7	-83.7
$CIBrCl^-$	1098.1	1095.7	-12.3	-48.9	-0.6	9.1	16.4	-85.6

Table S7. Gas Phase C–H Bond Dissociation Energies (Δ*H*) at 298 K in kJ/mol.

Reaction: $CHX_3 \longrightarrow CX_3^{\bullet} + H^{\bullet}$

Species	B3LYP/aT	MP2/aT	G3MP2	FPD(TQ)	FPD(Q5)	FPD(TQ5)
CH_4	431.7	424.6	435.7	436.5	436.5	436.3
CH_3F	413.1	411.6	422.6	421.8	421.8	421.7
CH_2F_2	413.0	415.1	424.6	424.3	424.1	424.0
CHF_3	432.7	436.6	446.2	445.7	445.2	445.2
CH_3Cl	413.5	406.7	415.7	414.8	413.6	413.7
CH_2Cl_2	391.4	396.9	402.2	401.7	400.0	400.2
$CHCl_3$	384.9	388.8	391.7	390.9	389.2	389.6
CH_3Br	418.4	413.0	420.8	420.7	420.5	420.4
CH_2Br_2	402.2	403.7	406.5	407.5	407.4	407.3
$CHBr_3$	384.1	392.1	391.0	392.3	392.4	392.4
CH_3I	420.5	415.2	421.9	421.8	421.6	421.5
CH_2I_2	400.5	402.4	403.7	404.9	404.8	404.7
CHI_3	376.9	385.0	382.5	384.1	384.0	384.0
CH_2FCl	403.1	406.9	414.5	413.9	413.0	413.1
CH_2FBr	411.3	409.8	416.3	416.3	416.1	416.1
CH_2FI	410.0	408.7	414.5	414.7	414.5	414.4
CH_2ClBr	400.1	400.3	404.4	404.6	403.8	403.9
CH_2ClI	399.3	399.4	402.2	403.4	402.5	402.6
CH_2BrI	401.4	402.9	404.6	406.2	406.1	406.0
CHF_2Cl	418.7	418.5	426.0	425.4	424.7	424.7
CHF_2Br	413.8	415.7	421.9	421.7	421.5	421.4
CHF_2I	404.9	407.4	413.3	413.4	413.1	413.1
$CHCl_2F$	401.5	403.5	408.7	408.1	406.8	407.0
$CHCl_2Br$	384.6	389.6	391.4	391.3	390.2	390.4
$CHCl_2I$	381.1	386.0	386.5	387.5	386.3	386.5
$CHBr_2F$	397.4	402.9	405.4	405.8	405.7	405.6
$CHBr_2Cl$	384.3	390.8	391.1	391.7	391.2	391.3
$CHBr_2I$	381.2	389.1	387.2	389.2	389.2	389.2
CHI_2F	388.2	395.0	396.4	397.1	396.9	396.9
CHI_2Cl	378.4	384.9	384.0	385.7	385.0	385.1
CHI_2Br	378.8	386.8	384.7	386.9	386.9	386.9
CHBrClF	399.4	403.0	406.9	406.8	406.1	406.2
CHIClF	393.8	397.8	400.9	401.4	400.7	400.7
CHIBrF	392.4	398.4	400.2	400.9	400.7	400.7
CHIBrCl	381.1	387.4	386.8	388.2	387.6	387.7

Table S8. Gas Phase Acidity (Δ*H*) at 298 K in kJ/mol.

Reaction: $CHX_3 \longrightarrow CX_3^- + H^+$

Species	B3LYP/aT	MP2/aT	G3MP2	FPD(TQ)	FPD(Q5)	FPD(TQ5)
CH_3^-	1739.1	1733.4	1751.5	1745.3	1744.7	1744.7
CH_2F^-	1706.5	1710.8	1720.7	1718.4	1718.1	1718.1
CHF_2^-	1657.7	1667.1	1672.6	1672.8	1672.1	1672.1
CF_3^-	1576.1	1586.7	1593.2	1592.1	1590.7	1590.8
CH_2Cl^-	1654.6	1658.8	1661.7	1663.9	1664.1	1664.1
$CHCl_2^-$	1568.2	1579.2	1576.4	1580.2	1580.6	1580.6
CCl_3^-	1491.0	1506.7	1500.2	1506.0	1506.6	1506.7
CH_2Br^-	1638.4	1645.1	1644.9	1646.1	1646.5	1646.5
$CHBr_2^-$	1543.3	1559.0	1549.9	1552.6	1553.2	1553.3
CBr_3^-	1463.0	1485.2	1470.3	1475.2	1475.7	1476.0
CH_2I^-	1619.8	1624.5	1626.5	1625.0	1625.3	1625.4
CHI_2^-	1519.2	1531.1	1524.9	1525.2	1525.5	1525.8
CI_3^-	1440.6	1455.2	1445.4	1448.4	1448.8	1449.2
$CHFCl^-$	1603.5	1616.3	1616.3	1618.9	1619.2	1619.2
$CHFBr^-$	1584.0	1601.3	1598.2	1598.7	1598.8	1598.9
$CHFI^-$	1563.7	1581.6	1581.7	1577.3	1577.4	1577.5
$CHClBr^-$	1555.0	1568.6	1562.8	1565.9	1566.3	1566.5
$CHClI^-$	1540.9	1553.7	1548.7	1550.4	1550.8	1550.9
$CHBrI^-$	1530.6	1544.7	1536.8	1538.3	1538.8	1539.0
CF_2Cl^-	1523.1	1544.8	1548.2	1545.4	1545.7	1545.6
CF_2Br^-	1497.9	1526.5	1520.7	1518.4	1518.3	1518.4
CF_2I^-	1472.5	1503.2	1494.3	1488.1	1488.2	1488.6
CCl_2F^-	1502.6	1523.4	1519.4	1522.9	1523.6	1523.6
CCl_2Br^-	1480.4	1498.9	1489.6	1494.8	1495.4	1495.6
CCl_2I^-	1469.5	1488.1	1478.7	1483.6	1484.1	1484.4
CBr_2F^-	1472.6	1501.8	1490.3	1491.7	1492.1	1492.3
CBr_2Cl^-	1471.1	1491.7	1479.6	1484.6	1485.1	1485.4
CBr_2I^-	1454.6	1475.1	1461.0	1465.7	1466.2	1466.5
CI_2F^-	1447.3	1475.9	1466.4	1466.2	1466.7	1467.0
CI_2Cl^-	1453.1	1471.2	1460.6	1464.6	1465.0	1465.3
CI_2Br^-	1447.2	1465.0	1452.8	1456.8	1457.2	1457.6
$CBrClF^-$	1486.5	1512.1	1505.6	1506.5	1507.1	1507.2
$CIClF^-$	1470.3	1497.8	1492.2	1490.4	1491.0	1491.1
$CIBrF^-$	1458.7	1488.4	1478.0	1477.3	1477.7	1478.0
$CIBrCl^-$	1461.5	1481.3	1469.4	1474.2	1474.7	1475.0

Table S9. Gas Phase Acidity (Δ*G*) at 298 K in kJ/mol.

Reaction: $CHX_3 \longrightarrow CX_3^- + H^+$

Species	B3LYP/aT	MP2/aT	G3MP2	FPD(TQ)	FPD(Q5)	FPD(TQ5)
CH_3^-	1704.4	1698.7	1716.8	1710.6	1710.0	1710.0
CH_2F^-	1671.5	1675.9	1685.6	1683.5	1683.2	1683.2
CHF_2^-	1624.5	1634.0	1639.5	1639.7	1639.1	1639.0
CF_3^-	1542.5	1553.2	1559.8	1558.7	1557.3	1557.3
CH_2Cl^-	1619.0	1623.7	1624.5	1628.8	1629.0	1629.0
$CHCl_2^-$	1533.9	1545.7	1541.3	1546.6	1547.0	1547.1
CCl_3^-	1458.2	1472.3	1465.0	1471.5	1472.1	1472.2
CH_2Br^-	1602.7	1610.0	1607.0	1611.0	1611.3	1611.4
$CHBr_2^-$	1507.1	1523.7	1512.6	1519.1	1519.6	1519.8
CBr_3^-	1427.2	1450.6	1437.3	1440.6	1441.1	1441.4
CH_2I^-	1584.2	1589.5	1588.7	1590.1	1590.3	1590.4
CHI_2^-	1483.3	1496.2	1487.8	1492.0	1492.4	1492.7
CI_3^-	1405.2	1418.2	1409.9	1411.4	1411.7	1412.1
$CHFCl^-$	1569.1	1582.6	1577.5	1585.3	1585.6	1585.5
$CHFBr^-$	1549.3	1567.5	1557.3	1564.9	1565.1	1565.2
$CHFI^-$	1528.7	1547.9	1540.9	1543.6	1543.6	1543.8
$CHClBr^-$	1520.6	1535.1	1527.0	1532.3	1532.8	1532.9
$CHClI^-$	1506.5	1520.3	1512.4	1517.0	1517.4	1517.6
$CHBrI^-$	1496.2	1511.3	1501.1	1504.9	1505.4	1505.6
CF_2Cl^-	1486.4	1509.5	1506.4	1510.1	1510.4	1510.3
CF_2Br^-	1460.5	1490.4	1477.2	1482.3	1482.2	1482.3
CF_2I^-	1434.2	1464.6	1450.9	1449.5	1449.7	1450.0
CCl_2F^-	1466.3	1488.6	1481.2	1488.0	1488.7	1488.7
CCl_2Br^-	1444.7	1464.4	1453.9	1460.3	1460.9	1461.1
CCl_2I^-	1433.5	1453.7	1442.0	1449.1	1449.6	1449.9
CBr_2F^-	1435.8	1466.6	1449.5	1456.5	1457.0	1457.2
CBr_2Cl^-	1435.3	1457.1	1443.8	1450.0	1450.6	1450.8
CBr_2I^-	1418.8	1440.5	1425.2	1431.2	1431.6	1432.0
CI_2F^-	1410.4	1440.9	1426.4	1431.2	1431.7	1432.0
CI_2Cl^-	1417.3	1436.7	1424.6	1430.1	1430.6	1430.9
CI_2Br^-	1411.6	1430.6	1417.1	1422.3	1422.8	1423.1
$CBrClF^-$	1449.9	1477.0	1463.7	1471.5	1472.1	1472.1
$CIClF^-$	1433.4	1462.8	1450.0	1455.3	1455.9	1456.0
$CIBrF^-$	1421.9	1453.3	1437.0	1442.2	1442.6	1442.9
$CIBrCl^-$	1425.6	1446.8	1433.4	1439.7	1440.2	1440.4

Table S10. Gas Phase Electron Affinity (Δ*H*) at 0 K in eV.

Reaction: $CX_3^{\bullet} + e^- \longrightarrow CX_3^-$

Species	B3LYP/aT	MP2/aT	G3MP2	FPD(TQ)	FPD(Q5)	FPD(TQ5)
$CH_3^{\bullet}$	0.11	0.03	0.01	0.06	0.07	0.06
$CH_2F^{\bullet}$	0.26	0.13	0.20	0.19	0.19	0.19
$CHF_2^{\bullet}$	0.77	0.63	0.73	0.69	0.70	0.70
$CF_3^{\bullet}$	1.83	1.69	1.77	1.75	1.76	1.76
$CH_2Cl^{\bullet}$	0.73	0.61	0.75	0.67	0.66	0.66
$CHCl_2^{\bullet}$	1.48	1.35	1.50	1.42	1.40	1.40
$CCl_3^{\bullet}$	2.15	2.03	2.18	2.08	2.06	2.06
$CH_2Br^{\bullet}$	0.94	0.82	0.98	0.92	0.91	0.91
$CHBr_2^{\bullet}$	1.78	1.63	1.82	1.76	1.76	1.75
$CBr_3^{\bullet}$	2.43	2.28	2.48	2.41	2.41	2.41
$CH_2I^{\bullet}$	1.16	1.05	1.18	1.14	1.14	1.14
$CHI_2^{\bullet}$	2.01	1.90	2.04	2.02	2.01	2.01
$CI_3^{\bullet}$	2.58	2.51	2.64	2.60	2.60	2.59
$CHFCl^{\bullet}$	1.24	1.07	1.22	1.14	1.13	1.13
$CHFBr^{\bullet}$	1.46	1.26	1.43	1.38	1.38	1.37
$CHFI^{\bullet}$	1.65	1.45	1.58	1.58	1.58	1.58
$CHClBr^{\bullet}$	1.64	1.50	1.66	1.60	1.58	1.58
$CHClI^{\bullet}$	1.77	1.64	1.78	1.74	1.73	1.73
$CHBrI^{\bullet}$	1.90	1.77	1.93	1.90	1.89	1.89
$CF_2Cl^{\bullet}$	2.17	1.94	2.06	2.03	2.02	2.03
$CF_2Br^{\bullet}$	2.38	2.10	2.30	2.28	2.28	2.27
$CF_2I^{\bullet}$	2.55	2.26	2.48	2.51	2.51	2.50
$CCl_2F^{\bullet}$	2.21	2.01	2.17	2.09	2.07	2.07
$CCl_2Br^{\bullet}$	2.26	2.12	2.29	2.20	2.18	2.18
$CCl_2I^{\bullet}$	2.34	2.19	2.35	2.28	2.26	2.26
$CBr_2F^{\bullet}$	2.47	2.22	2.44	2.39	2.38	2.38
$CBr_2Cl^{\bullet}$	2.35	2.20	2.39	2.31	2.30	2.30
$CBr_2I^{\bullet}$	2.49	2.35	2.54	2.48	2.47	2.47
$CI_2F^{\bullet}$	2.64	2.41	2.59	2.56	2.55	2.55
$CI_2Cl^{\bullet}$	2.47	2.35	2.50	2.45	2.44	2.44
$CI_2Br^{\bullet}$	2.54	2.43	2.59	2.55	2.54	2.54
$CBrClF^{\bullet}$	2.35	2.12	2.30	2.24	2.23	2.23
$CIClF^{\bullet}$	2.46	2.21	2.37	2.35	2.34	2.34
$CIBrF^{\bullet}$	2.56	2.32	2.51	2.48	2.48	2.47
$CIBrCl^{\bullet}$	2.42	2.27	2.45	2.38	2.37	2.37

Table S11. ΔG_{solv} for Neutral Species in kJ/mol in Water.

Species	B3LYP/aT/SMD	B3LYP/aT/COSMO	MP2/aT/SMD	MP2/aT/COSMO
Benzoic Acid	-14.7	-23.8	-12.6	-22.4
CH_4	17.1	6.3	16.9	6.2
CH_3F	6.5	-4.3	6.4	-4.4
CH_2F_2	2.9	-8.1	2.8	-8.2
CHF_3	7.1	-5.2	7.3	-5.1
CH_3Cl	4.3	-3.9	4.8	-3.4
CH_2Cl_2	-0.5	-6.8	0.0	-6.2
$CHCl_3$	3.2	-3.8	3.5	-3.3
CH_3Br	4.2	-3.2	4.5	-2.7
CH_2Br_2	-1.2	-6.2	-0.8	-5.7
$CHBr_3$	-0.7	-4.5	-0.6	-3.9
CH_3I	4.2	-2.4	4.7	-2.0
CH_2I_2	-6.5	-6.0	-5.6	-5.5
CHI_3	-15.2	-6.1	-13.5	-5.4
CH_2FCl	0.9	-7.6	1.2	-7.2
CH_2FBr	1.0	-7.4	1.2	-7.0
CH_2FI	-2.1	-7.2	-1.8	-6.8
CH_2ClBr	-0.5	-6.5	-0.1	-5.9
CH_2ClI	-4.3	-6.3	-3.6	-5.8
CH_2BrI	-5.0	-6.1	-4.5	-5.6
CHF_2Cl	5.8	-4.7	6.1	-4.4
CHF_2Br	5.1	-5.0	5.5	-4.6
CHF_2I	-1.1	-5.5	-0.7	-5.0
$CHCl_2F$	4.5	-4.3	4.8	-3.9
$CHCl_2Br$	2.2	-4.1	2.4	-3.6
$CHCl_2I$	-4.6	-4.7	-4.0	-4.1
$CHBr_2F$	2.4	-4.8	2.7	-4.3
$CHBr_2Cl$	0.8	-4.4	1.1	-3.9
$CHBr_2I$	-7.2	-5.2	-6.7	-4.6
CHI_2F	-8.8	-5.7	-8.0	-5.2
CHI_2Cl	-10.9	-5.5	-9.8	-4.9
CHI_2Br	-12.4	-5.7	-11.5	-5.0
CHBrClF	3.7	-4.5	3.9	-4.1
CHIClF	-2.9	-5.0	-2.4	-4.5
CHIBrF	-3.9	-5.2	-3.5	-4.7
CHIBrCl	-5.5	-4.9	-5.0	-4.3

Table S12. ΔG_{solv} for Neutral Species in kJ/mol in DMSO.

Species	B3LYP/aT/SMD	B3LYP/aT/COSMO	MP2/aT/SMD	MP2/aT/COSMO
Benzoic Acid	-21.1	-23.4	-20.9	-22.1
CH_4	12.8	6.3	12.6	6.2
CH_3F	1.8	-4.1	1.7	-4.3
CH_2F_2	-2.4	-7.9	-2.4	-8.0
CHF_3	1.3	-5.1	1.6	-4.9
CH_3Cl	-2.5	-3.8	-2.0	-3.2
CH_2Cl_2	-9.8	-6.6	-9.2	-6.1
$CHCl_3$	-8.4	-3.7	-8.1	-3.2
CH_3Br	-2.7	-3.1	-2.3	-2.6
CH_2Br_2	-10.6	-6.1	-10.2	-5.5
$CHBr_3$	-12.5	-4.4	-12.2	-3.8
CH_3I	6.2	-2.3	6.7	-1.9
CH_2I_2	1.5	-5.8	2.3	-5.3
CHI_3	-1.8	-5.9	-0.3	-5.2
CH_2FCl	-6.4	-7.4	-6.0	-7.1
CH_2FBr	-6.4	-7.2	-6.1	-6.8
CH_2FI	-0.6	-7.0	-0.3	-6.7
CH_2ClBr	-9.9	-6.3	-9.4	-5.8
CH_2ClI	-4.9	-6.2	-4.3	-5.6
CH_2BrI	-5.7	-5.9	-5.1	-5.4
CHF_2Cl	-2.0	-4.6	-1.7	-4.3
CHF_2Br	-2.8	-4.8	-2.4	-4.4
CHF_2I	-0.2	-5.3	0.2	-4.9
$CHCl_2F$	-5.3	-4.1	-4.9	-3.7
$CHCl_2Br$	-9.5	-4.0	-9.2	-3.4
$CHCl_2I$	-7.8	-4.6	-7.1	-4.0
$CHBr_2F$	-7.4	-4.6	-7.1	-4.2
$CHBr_2Cl$	-10.9	-4.3	-10.5	-3.8
$CHBr_2I$	-10.4	-5.1	-10.0	-4.4
CHI_2F	-1.4	-5.6	-0.7	-5.1
CHI_2Cl	-5.7	-5.4	-4.7	-4.7
CHI_2Br	-7.2	-5.6	-6.4	-4.9
CHBrClF	-6.1	-4.4	-5.8	-4.0
CHIClF	-4.1	-4.9	-3.5	-4.4
CHIBrF	-5.1	-5.1	-4.6	-4.6
CHIBrCl	-8.7	-4.8	-8.2	-4.2

Table S13. ΔG_{solv} for Neutral Species in kJ/mol in Acetonitrile.

Species	B3LYP/aT/SMD	B3LYP/aT/COSMO	MP2/aT/SMD	MP2/aT/COSMO
Benzoic Acid	-25.7	-23.2	-25.5	-21.8
CH_4	9.7	6.4	9.5	6.2
CH_3F	-1.3	-4.0	-1.4	-4.2
CH_2F_2	-5.4	-7.8	-5.4	-7.9
CHF_3	-1.7	-5.0	-1.5	-4.8
CH_3Cl	-5.5	-3.7	-5.0	-3.2
CH_2Cl_2	-12.7	-6.5	-12.2	-5.9
$CHCl_3$	-11.5	-3.6	-11.1	-3.1
CH_3Br	-5.7	-3.0	-5.3	-2.5
CH_2Br_2	-13.5	-6.0	-13.1	-5.4
$CHBr_3$	-15.5	-4.3	-15.2	-3.7
CH_3I	2.5	-2.2	3.0	-1.8
CH_2I_2	-2.8	-5.7	-2.0	-5.2
CHI_3	-6.7	-5.8	-5.1	-5.1
CH_2FCl	-9.4	-7.3	-9.0	-7.0
CH_2FBr	-9.4	-7.1	-9.0	-6.7
CH_2FI	-4.3	-6.9	-3.9	-6.6
CH_2ClBr	-12.9	-6.2	-12.3	-5.7
CH_2ClI	-8.5	-6.1	-7.8	-5.5
CH_2BrI	-9.3	-5.8	-8.7	-5.3
CHF_2Cl	-5.1	-4.5	-4.7	-4.2
CHF_2Br	-5.8	-4.7	-5.4	-4.3
CHF_2I	-3.9	-5.2	-3.4	-4.8
$CHCl_2F$	-8.4	-4.0	-8.0	-3.6
$CHCl_2Br$	-12.6	-3.9	-12.2	-3.4
$CHCl_2I$	-11.4	-4.5	-10.7	-3.9
$CHBr_2F$	-10.5	-4.5	-10.1	-4.1
$CHBr_2Cl$	-13.9	-4.2	-13.5	-3.7
$CHBr_2I$	-14.0	-5.0	-13.5	-4.3
CHI_2F	-5.7	-5.5	-5.0	-5.0
CHI_2Cl	-9.9	-5.3	-8.9	-4.6
CHI_2Br	-11.5	-5.5	-10.6	-4.8
CHBrClF	-9.2	-4.3	-8.8	-3.9
CHIClF	-7.7	-4.8	-7.2	-4.3
CHIBrF	-8.8	-5.0	-8.3	-4.5
CHIBrCl	-12.4	-4.7	-11.8	-4.1

Table S14. ΔG_{solv} for Neutral Species in kJ/mol in THF.

Species	B3LYP/aT/SMD	B3LYP/aT/COSMO	MP2/aT/SMD	MP2/aT/COSMO
Benzoic Acid	-23.6	-19.3	-23.3	-18.1
CH_4	9.9	6.5	9.8	6.4
CH_3F	0.7	-2.5	0.7	-2.6
CH_2F_2	-2.4	-5.8	-2.5	-5.9
CHF_3	0.9	-3.4	1.1	-3.2
CH_3Cl	-3.9	-2.2	-3.5	-1.7
CH_2Cl_2	-10.6	-4.6	-10.2	-4.2
$CHCl_3$	-10.4	-2.2	-10.0	-1.7
CH_3Br	-4.7	-1.6	-4.4	-1.2
CH_2Br_2	-12.7	-4.2	-12.3	-3.7
$CHBr_3$	-15.9	-2.8	-15.7	-2.3
CH_3I	4.1	-0.9	4.5	-0.6
CH_2I_2	0.0	-4.0	0.6	-3.5
CHI_3	-2.9	-4.1	-1.7	-3.5
CH_2FCl	-6.8	-5.3	-6.5	-5.1
CH_2FBr	-7.4	-5.2	-7.2	-4.9
CH_2FI	-1.4	-5.0	-1.1	-4.7
CH_2ClBr	-11.4	-4.4	-11.0	-3.9
CH_2ClI	-5.9	-4.3	-5.4	-3.8
CH_2BrI	-7.3	-4.1	-6.8	-3.6
CHF_2Cl	-2.9	-2.9	-2.6	-2.7
CHF_2Br	-4.3	-3.1	-3.9	-2.8
CHF_2I	-0.7	-3.6	-0.4	-3.2
$CHCl_2F$	-6.7	-2.5	-6.4	-2.2
$CHCl_2Br$	-12.0	-2.4	-11.7	-1.9
$CHCl_2I$	-9.0	-2.9	-8.5	-2.4
$CHBr_2F$	-9.9	-3.0	-9.7	-2.6
$CHBr_2Cl$	-13.9	-2.7	-13.5	-2.2
$CHBr_2I$	-12.6	-3.4	-12.2	-2.8
CHI_2F	-2.2	-3.8	-1.6	-3.4
CHI_2Cl	-6.6	-3.6	-5.8	-3.1
CHI_2Br	-8.5	-3.8	-7.9	-3.2
CHBrClF	-8.1	-2.7	-7.9	-2.4
CHIClF	-5.0	-3.2	-4.6	-2.8
CHIBrF	-6.6	-3.4	-6.2	-2.9
CHIBrCl	-10.6	-3.1	-10.1	-2.6

Table S15. ΔG_{solv} for Anion Species in kJ/mol in Water.

Species	B3LYP/aT/SMD	B3LYP/aT/COSMO	MP2/aT/SMD	MP2/aT/COSMO
Benzoate	-258.1	-261.1	-256.5	-260.7
CH_4	-276.5	-284.4	-279.1	-286.8
CH_3F	-269.0	-280.7	-272.7	-283.9
CH_2F_2	-255.3	-270.7	-258.5	-273.6
CHF_3	-236.5	-255.0	-239.2	-257.7
CH_3Cl	-239.4	-256.4	-244.0	-259.6
CH_2Cl_2	-208.9	-228.8	-214.1	-232.5
$CHCl_3$	-188.1	-207.5	-193.6	-211.8
CH_3Br	-216.6	-245.9	-222.3	-249.7
CH_2Br_2	-180.6	-213.8	-186.1	-218.5
$CHBr_3$	-164.2	-191.9	-168.5	-196.9
CH_3I	-216.3	-231.3	-221.3	-235.2
CH_2I_2	-174.9	-194.6	-180.3	-199.1
CHI_3	-150.9	-172.6	-155.0	-176.3
CH_2FCl	-224.9	-245.5	-230.7	-249.2
CH_2FBr	-202.4	-234.7	-210.8	-239.2
CH_2FI	-200.6	-219.9	-207.0	-224.7
CH_2ClBr	-193.6	-220.9	-199.5	-225.2
CH_2ClI	-191.4	-209.3	-197.2	-214.1
CH_2BrI	-181.4	-203.6	-186.7	-208.2
CHF_2Cl	-207.3	-234.9	-211.1	-234.5
CHF_2Br	-182.9	-226.6	-188.7	-225.3
CHF_2I	-190.2	-213.8	-192.5	-215.6
$CHCl_2F$	-193.3	-216.4	-199.8	-220.5
$CHCl_2Br$	-178.2	-201.4	-184.2	-206.3
$CHCl_2I$	-174.4	-191.9	-180.6	-197.3
$CHBr_2F$	-167.9	-202.3	-175.5	-206.7
$CHBr_2Cl$	-170.0	-196.3	-175.5	-201.4
$CHBr_2I$	-164.3	-184.4	-168.5	-189.2
CHI_2F	-159.2	-183.6	-165.0	-187.6
CHI_2Cl	-162.1	-180.7	-167.9	-185.7
CHI_2Br	-159.9	-178.0	-164.3	-182.4
CHBrClF	-178.8	-209.0	-186.8	-213.1
CHIClF	-176.4	-197.7	-181.6	-201.7
CHIBrF	-168.1	-192.2	-173.4	-196.4
CHIBrCl	-168.7	-187.9	-174.1	-193.0

Table S16. ΔG_{solv} for Anion Species in kJ/mol in DMSO.

Species	B3LYP/aT/SMD	B3LYP/aT/COSMO	MP2/aT/SMD	MP2/aT/COSMO
Benzoate	-223.9	-258.6	-225.1	-258.1
CH_4	-276.6	-281.7	-279.2	-284.1
CH_3F	-269.9	-278.1	-273.6	-281.3
CH_2F_2	-257.0	-268.2	-260.2	-271.1
CHF_3	-239.0	-252.7	-241.6	-255.4
CH_3Cl	-242.7	-254.1	-247.2	-257.2
CH_2Cl_2	-215.1	-226.7	-220.2	-230.4
$CHCl_3$	-196.9	-205.6	-202.1	-209.9
CH_3Br	-220.1	-243.6	-225.6	-247.4
CH_2Br_2	-187.1	-211.8	-192.4	-216.5
$CHBr_3$	-173.4	-190.1	-177.4	-195.1
CH_3I	-210.7	-229.2	-215.7	-233.0
CH_2I_2	-163.9	-192.8	-169.5	-197.2
CHI_3	-134.9	-170.9	-139.3	-174.7
CH_2FCl	-229.0	-243.2	-234.7	-246.9
CH_2FBr	-206.7	-232.5	-214.9	-237.0
CH_2FI	-195.8	-217.9	-202.3	-222.6
CH_2ClBr	-200.0	-218.8	-205.7	-223.1
CH_2ClI	-189.0	-207.4	-194.8	-212.1
CH_2BrI	-179.0	-201.7	-184.4	-206.3
CHF_2Cl	-212.2	-232.7	-215.8	-232.3
CHF_2Br	-188.1	-224.5	-193.7	-223.2
CHF_2I	-185.8	-211.8	-188.2	-213.6
$CHCl_2F$	-200.3	-214.4	-206.6	-218.4
$CHCl_2Br$	-187.1	-199.6	-192.8	-204.4
$CHCl_2I$	-174.7	-190.1	-180.9	-195.4
$CHBr_2F$	-175.3	-200.4	-182.4	-204.8
$CHBr_2Cl$	-179.1	-194.5	-184.2	-199.5
$CHBr_2I$	-165.0	-182.7	-169.1	-187.5
CHI_2F	-148.9	-181.9	-142.4	-175.6
CHI_2Cl	-154.2	-179.0	-160.1	-183.9
CHI_2Br	-152.2	-176.3	-156.7	-180.7
CHBrClF	-186.0	-207.0	-193.6	-211.2
CHIClF	-174.7	-195.9	-179.9	-199.8
CHIBrF	-166.5	-190.4	-171.8	-194.6
CHIBrCl	-169.3	-186.1	-174.6	-191.2

Table S17. ΔG_{solv} for Anion Species in kJ/mol in Acetonitrile.

Species	B3LYP/aT/SMD	B3LYP/aT/COSMO	MP2/aT/SMD	MP2/aT/COSMO
Benzoate	-231.0	-256.6	-232.0	-256.1
CH_4	-278.2	-279.6	-280.8	-282.0
CH_3F	-271.8	-276.1	-275.4	-279.2
CH_2F_2	-259.1	-266.3	-262.2	-269.1
CHF_3	-241.3	-250.9	-243.9	-253.6
CH_3Cl	-244.8	-252.2	-249.2	-255.3
CH_2Cl_2	-217.6	-225.1	-222.5	-228.7
$CHCl_3$	-199.6	-204.1	-204.7	-208.4
CH_3Br	-222.3	-241.8	-227.7	-245.6
CH_2Br_2	-189.7	-210.3	-194.9	-214.9
$CHBr_3$	-176.2	-188.7	-180.1	-193.6
CH_3I	-213.7	-227.5	-218.5	-231.3
CH_2I_2	-167.9	-191.4	-173.3	-195.7
CHI_3	-139.8	-169.7	-144.0	-173.4
CH_2FCl	-231.4	-241.5	-237.0	-245.1
CH_2FBr	-209.3	-230.9	-217.2	-235.3
CH_2FI	-199.1	-216.3	-205.4	-221.0
CH_2ClBr	-202.6	-217.2	-208.1	-221.5
CH_2ClI	-192.2	-205.9	-197.8	-210.5
CH_2BrI	-182.3	-200.2	-187.5	-204.7
CHF_2Cl	-215.0	-231.1	-218.4	-230.7
CHF_2Br	-191.1	-222.9	-196.5	-221.6
CHF_2I	-189.5	-210.2	-191.7	-212.1
$CHCl_2F$	-203.1	-212.9	-209.2	-216.9
$CHCl_2Br$	-189.9	-198.1	-195.4	-202.9
$CHCl_2I$	-178.3	-188.8	-184.2	-194.0
$CHBr_2F$	-178.3	-199.0	-185.2	-203.3
$CHBr_2Cl$	-181.9	-193.1	-186.9	-198.0
$CHBr_2I$	-168.5	-181.3	-172.4	-186.1
CHI_2F	-153.3	-180.5	-146.8	-174.3
CHI_2Cl	-158.4	-177.7	-164.1	-182.6
CHI_2Br	-156.4	-175.0	-160.7	-179.4
CHBrClF	-188.9	-205.6	-196.3	-209.7
CHIClF	-178.3	-194.5	-183.3	-198.3
CHIBrF	-170.1	-189.0	-175.3	-193.2
CHIBrCl	-172.8	-184.7	-177.9	-189.8

Table S18. ΔG_{solv} for Anion Species in kJ/mol in THF.

Species	B3LYP/aT/SMD	B3LYP/aT/COSMO	MP2/aT/SMD	MP2/aT/COSMO
Benzoate	-200.7	-225.1	-201.6	-224.8
CH_4	-241.7	-246.1	-244.1	-248.3
CH_3F	-237.0	-243.7	-240.3	-246.6
CH_2F_2	-226.7	-235.6	-229.6	-238.1
CHF_3	-211.9	-222.3	-214.2	-224.7
CH_3Cl	-214.3	-222.8	-218.0	-225.5
CH_2Cl_2	-191.5	-199.0	-195.5	-202.2
$CHCl_3$	-177.1	-180.4	-181.0	-184.2
CH_3Br	-194.4	-213.6	-198.8	-216.9
CH_2Br_2	-167.7	-185.8	-171.8	-189.8
$CHBr_3$	-157.6	-166.7	-160.7	-171.0
CH_3I	-185.4	-200.7	-189.5	-204.1
CH_2I_2	-144.9	-168.8	-149.6	-172.6
CHI_3	-120.1	-149.6	-123.5	-152.8
CH_2FCl	-203.1	-213.6	-207.9	-216.8
CH_2FBr	-183.7	-204.1	-190.3	-208.0
CH_2FI	-173.1	-191.1	-178.7	-195.3
CH_2ClBr	-178.7	-192.0	-183.2	-195.7
CH_2ClI	-167.8	-181.8	-172.5	-185.8
CH_2BrI	-159.4	-176.7	-163.7	-180.7
CHF_2Cl	-189.3	-204.5	-192.3	-204.2
CHF_2Br	-168.7	-197.1	-173.4	-196.1
CHF_2I	-164.5	-185.7	-166.6	-187.3
$CHCl_2F$	-179.4	-188.4	-184.5	-191.9
$CHCl_2Br$	-168.7	-175.1	-173.1	-179.3
$CHCl_2I$	-156.7	-166.7	-161.6	-171.3
$CHBr_2F$	-158.5	-175.9	-164.0	-179.8
$CHBr_2Cl$	-162.2	-170.6	-166.1	-174.9
$CHBr_2I$	-148.9	-160.0	-152.2	-164.2
CHI_2F	-132.9	-159.5	-126.3	-153.2
CHI_2Cl	-137.9	-156.8	-142.6	-161.0
CHI_2Br	-136.6	-154.4	-140.1	-158.2
CHBrClF	-167.4	-181.8	-173.5	-185.4
CHIClF	-156.1	-171.9	-160.6	-175.3
CHIBrF	-149.3	-167.0	-153.7	-170.7
CHIBrCl	-152.3	-163.1	-156.5	-167.5

Table S19. Absolute pK_as in Water.

Solvation	SMD	COSMO	SMD	COSMO	SMD	COSMO
Gas	B3LYP/aT	B3LYP/aT	MP2/aT	MP2/aT	G3MP2	G3MP2
Benzoic Acid	7.0	8.1	6.9	6.9	9.3	10.2
CH_4	54.8	55.3	53.4	53.9	56.6	57.1
CH_3F	52.2	52.1	52.4	52.3	54.1	54.0
CH_2F_2	47.0	46.2	48.1	47.4	49.1	48.4
CHF_3	35.2	34.1	36.6	35.5	37.7	36.7
CH_3Cl	48.6	47.1	48.5	47.2	48.6	47.4
CH_2Cl_2	39.9	37.5	40.9	38.8	40.2	38.0
$CHCl_3$	29.6	27.4	31.1	29.1	29.8	27.8
CH_3Br	49.8	45.9	50.0	46.4	49.4	45.9
CH_2Br_2	40.3	35.3	42.1	37.3	40.2	35.4
$CHBr_3$	29.0	24.9	32.4	28.0	30.0	25.7
CH_3I	46.6	45.1	46.5	45.3	46.4	45.1
CH_2I_2	38.0	34.5	39.2	35.9	37.7	34.4
CHI_3	30.1	24.7	31.3	26.2	29.9	24.7
CH_2FCl	43.0	40.9	44.3	42.5	43.4	41.6
CH_2FBr	43.4	39.2	45.1	41.6	43.3	39.8
CH_2FI	40.7	38.2	42.9	40.7	41.7	39.4
CH_2ClBr	40.2	36.5	41.7	38.2	40.2	36.8
CH_2ClI	38.8	36.0	40.1	37.5	38.7	36.1
CH_2BrI	38.9	35.2	40.5	36.9	38.7	35.1
CHF_2Cl	30.7	27.7	34.1	31.8	33.5	31.3
CHF_2Br	30.6	24.7	34.7	30.1	32.4	27.8
CHF_2I	25.8	22.4	30.6	27.4	28.2	25.0
$CHCl_2F$	29.9	27.4	32.6	30.5	31.3	29.2
$CHCl_2Br$	29.2	26.2	31.5	28.7	29.7	26.9
$CHCl_2I$	29.1	26.0	31.4	28.5	29.3	26.4
$CHBr_2F$	29.3	24.6	33.4	29.1	30.4	26.1
$CHBr_2Cl$	29.2	25.5	32.0	28.3	29.7	26.0
$CHBr_2I$	28.7	24.8	31.7	27.7	29.0	25.0
CHI_2F	28.4	23.6	32.6	28.1	30.0	25.6
CHI_2Cl	29.5	25.3	31.7	27.7	29.5	25.6
CHI_2Br	29.1	24.8	31.5	27.2	29.1	24.8
CHBrClF	29.7	25.9	33.0	29.8	30.7	27.5
CHIClF	28.4	25.0	32.5	29.4	30.3	27.2
CHIBrF	28.0	24.0	32.5	28.7	29.6	25.8
CHIBrCl	28.8	25.4	31.5	28.1	29.2	25.7

Table S20. Absolute p*K*ₐs in DMSO.

Solvation	SMD	COSMO	SMD	COSMO	SMD	COSMO
Gas	B3LYP/aT	B3LYP/aT	MP2/aT	MP2/aT	G3MP2	G3MP2
Benzoic Acid	6.1	0.5	5.9	0.3	8.3	2.7
CH_4	47.6	47.8	46.1	46.4	49.3	49.6
CH_3F	44.9	44.5	45.1	44.7	46.8	46.4
CH_2F_2	39.7	38.7	40.8	39.8	41.8	40.8
CHF_3	27.8	26.5	29.2	27.9	30.3	29.1
CH_3Cl	41.2	39.5	41.2	39.6	41.3	39.8
CH_2Cl_2	32.4	29.8	33.5	31.2	32.7	30.4
$CHCl_3$	22.1	19.8	23.6	21.4	22.3	20.1
CH_3Br	42.4	38.3	42.6	38.8	42.1	38.3
CH_2Br_2	32.8	27.7	34.7	29.7	32.7	27.7
$CHBr_3$	21.5	17.2	24.9	20.3	22.6	18.0
CH_3I	39.2	37.5	39.2	37.6	39.0	37.5
CH_2I_2	30.6	26.8	31.7	28.2	30.2	26.7
CHI_3	22.5	16.9	23.8	18.4	22.3	17.0
CH_2FCl	35.6	33.2	36.9	34.9	36.0	34.0
CH_2FBr	36.0	31.6	37.7	34.0	35.9	32.2
CH_2FI	33.3	30.6	35.5	33.0	34.2	31.8
CH_2ClBr	32.8	28.8	34.2	30.5	32.8	29.1
CH_2ClI	31.3	28.3	32.6	29.9	31.3	28.5
CH_2BrI	31.4	27.5	33.0	29.3	31.3	27.5
CHF_2Cl	23.2	20.1	26.6	24.2	26.1	23.6
CHF_2Br	23.1	17.0	27.3	22.4	24.9	20.1
CHF_2I	18.4	14.8	23.2	19.7	20.9	17.3
$CHCl_2F$	22.4	19.7	25.1	22.8	23.8	21.5
$CHCl_2Br$	21.6	18.5	24.0	21.0	22.2	19.2
$CHCl_2I$	21.6	18.3	23.9	20.8	21.9	18.8
$CHBr_2F$	21.8	16.9	25.9	21.5	22.9	18.5
$CHBr_2Cl$	21.7	17.8	24.5	20.7	22.2	18.3
$CHBr_2I$	21.2	17.1	24.2	20.0	21.5	17.3
CHI_2F	20.9	15.9	27.3	22.2	24.7	19.7
CHI_2Cl	22.0	17.6	24.1	20.0	22.0	17.9
CHI_2Br	21.6	17.1	24.0	19.5	21.6	17.1
CHBrClF	22.2	18.2	25.5	22.1	23.2	19.8
CHIClF	20.9	17.3	25.0	21.7	22.8	19.5
CHIBrF	20.5	16.3	25.0	21.0	22.1	18.1
CHIBrCl	21.3	17.7	24.0	20.4	21.7	18.0

Table S21. Absolute pK_as in MeCN.

Solvation	SMD	COSMO	SMD	COSMO	SMD	COSMO
Gas	B3LYP/aT	B3LYP/aT	MP2/aT	MP2/aT	G3MP2	G3MP2
Benzoic Acid	20.4	15.5	20.2	15.3	22.6	17.7
CH_4	62.6	62.9	61.2	61.5	64.3	64.7
CH_3F	59.9	59.6	60.0	59.8	61.7	61.5
CH_2F_2	54.6	53.7	55.7	54.9	56.7	55.9
CHF_3	42.7	41.5	44.0	42.9	45.2	44.1
CH_3Cl	56.1	54.5	56.1	54.7	56.2	54.8
CH_2Cl_2	47.2	44.8	48.3	46.2	47.6	45.4
$CHCl_3$	36.9	34.7	38.4	36.4	37.1	35.1
CH_3Br	57.2	53.3	57.5	53.9	57.0	53.3
CH_2Br_2	47.6	42.6	49.5	44.7	47.5	42.7
$CHBr_3$	36.3	32.1	39.7	35.3	37.3	32.9
CH_3I	54.1	52.5	54.1	52.7	53.9	52.5
CH_2I_2	45.3	41.7	46.5	43.1	45.0	41.7
CHI_3	37.3	31.9	38.5	33.4	37.1	31.9
CH_2FCl	50.4	48.3	51.7	49.9	50.8	49.0
CH_2FBr	50.8	46.6	52.5	49.0	50.8	47.2
CH_2FI	48.1	45.5	50.3	48.0	49.1	46.8
CH_2ClBr	47.6	43.8	49.0	45.5	47.6	44.1
CH_2ClI	46.1	43.3	47.5	44.8	46.1	43.5
CH_2BrI	46.2	42.5	47.8	44.2	46.1	42.4
CHF_2Cl	38.0	35.1	41.4	39.2	40.9	38.6
CHF_2Br	37.8	32.0	42.0	37.4	39.7	35.1
CHF_2I	33.1	29.8	38.0	34.7	35.6	32.3
$CHCl_2F$	37.2	34.7	39.9	37.8	38.6	36.5
$CHCl_2Br$	36.4	33.5	38.8	36.0	37.0	34.2
$CHCl_2I$	36.3	33.3	38.7	35.8	36.6	33.7
$CHBr_2F$	36.5	31.9	40.7	36.4	37.7	33.4
$CHBr_2Cl$	36.4	32.8	39.3	35.6	37.0	33.3
$CHBr_2I$	35.9	32.1	38.9	34.9	36.2	32.3
CHI_2F	35.6	30.8	42.0	37.2	39.4	34.6
CHI_2Cl	36.7	32.5	38.9	34.9	36.8	32.8
CHI_2Br	36.3	32.0	38.7	34.4	36.4	32.1
CHBrClF	36.9	33.2	40.3	37.1	38.0	34.8
CHIClF	35.6	32.3	39.8	36.7	37.6	34.4
CHIBrF	35.2	31.3	39.8	36.0	36.9	33.1
CHIBrCl	36.1	32.6	38.8	35.3	36.4	33.0

Table S22. Absolute pK_as in THF.

Solvation	SMD	COSMO	SMD	COSMO	SMD	COSMO
Gas	B3LYP/aT	B3LYP/aT	MP2/aT	MP2/aT	G3MP2	G3MP2
Benzoic Acid	22.6	17.6	22.4	17.4	24.7	19.8
CH_4	66.1	65.9	64.7	64.6	67.9	67.8
CH_3F	62.8	62.2	63.0	62.5	64.7	64.2
CH_2F_2	56.9	56.0	58.1	57.2	59.1	58.2
CHF_3	44.6	43.5	46.0	44.9	47.2	46.1
CH_3Cl	58.4	56.6	58.5	56.9	58.6	57.0
CH_2Cl_2	48.7	46.3	49.9	47.7	49.2	46.9
$CHCl_3$	37.9	35.8	39.6	37.6	38.3	36.3
CH_3Br	59.2	55.3	59.6	55.9	59.1	55.4
CH_2Br_2	48.5	43.8	50.6	46.0	48.7	44.0
$CHBr_3$	36.8	32.9	40.3	36.2	38.0	33.9
CH_3I	56.0	54.2	56.1	54.4	56.0	54.3
CH_2I_2	46.1	42.6	47.4	44.1	46.0	42.6
CHI_3	37.3	32.3	38.7	33.9	37.3	32.5
CH_2FCl	52.1	50.0	53.6	51.8	52.7	50.9
CH_2FBr	52.1	48.2	54.1	50.6	52.4	48.9
CH_2FI	49.4	46.8	51.7	49.4	50.5	48.2
CH_2ClBr	48.7	45.2	50.4	47.0	49.0	45.5
CH_2ClI	47.2	44.4	48.7	46.1	47.3	44.7
CH_2BrI	47.1	43.5	48.9	45.4	47.1	43.6
CHF_2Cl	39.4	36.7	42.8	40.8	42.3	40.2
CHF_2Br	38.7	33.5	43.0	38.8	40.7	36.5
CHF_2I	34.2	31.0	39.1	35.9	36.7	33.5
$CHCl_2F$	38.2	35.9	41.2	39.2	39.9	37.9
$CHCl_2Br$	37.3	34.4	39.9	37.1	38.0	35.3
$CHCl_2I$	36.9	34.1	39.5	36.7	37.4	34.7
$CHBr_2F$	37.1	32.9	41.5	37.5	38.5	34.5
$CHBr_2Cl$	37.1	33.7	40.2	36.6	37.8	34.3
$CHBr_2I$	36.3	32.7	39.5	35.7	36.8	33.0
CHI_2F	35.8	31.4	42.2	37.8	39.7	35.3
CHI_2Cl	36.9	33.1	39.4	35.6	37.2	33.5
CHI_2Br	36.5	32.5	39.1	35.1	36.7	32.7
CHBrClF	37.7	34.3	41.4	38.3	39.0	36.0
CHIClF	36.3	33.2	40.5	37.7	38.3	35.4
CHIBrF	35.7	32.0	40.4	36.8	37.5	34.0
CHIBrCl	36.6	33.3	39.4	36.2	37.1	33.8

Table S23. Water: Relative Solution Acidities (ΔG_{298}) in kJ/mol and pK_a Values at the B3LYP/aT Level.

Species	ΔG_{298}	ΔG_{298}	pK_a	pK_a
Implicit	COSMO	SMD	COSMO	SMD
CH_4	269.7	272.8	51.5	52.0
CH_3F	251.1	258.0	48.2	49.5
CH_2F_2	217.9	228.4	42.4	44.3
CHF_3	148.7	160.9	30.3	32.4
CH_3Cl	222.6	237.4	43.2	45.8
CH_2Cl_2	167.8	187.5	33.7	37.1
$CHCl_3$	110.6	128.9	23.6	26.8
CH_3Br	216.1	244.0	42.1	47.0
CH_2Br_2	155.6	189.8	31.5	37.5
$CHBr_3$	95.9	125.8	21.0	26.3
CH_3I	211.4	225.8	41.3	43.8
CH_2I_2	150.7	177.0	30.7	35.3
CHI_3	94.7	131.6	20.8	27.3
CH_2FCl	187.2	205.3	37.0	40.2
CH_2FBr	178.0	207.9	35.4	40.7
CH_2FI	172.1	192.3	34.4	37.9
CH_2ClBr	162.3	189.5	32.7	37.5
CH_2ClI	159.5	181.4	32.2	36.0
CH_2BrI	154.8	181.9	31.4	36.1
CHF_2Cl	112.3	135.3	23.9	28.0
CHF_2Br	94.9	134.5	20.9	27.8
CHF_2I	82.0	107.2	18.6	23.0
$CHCl_2F$	110.2	130.6	23.6	27.1
$CHCl_2Br$	103.4	126.3	22.4	26.4
$CHCl_2I$	102.4	125.8	22.2	26.3
$CHBr_2F$	94.3	127.5	20.8	26.6
$CHBr_2Cl$	99.5	126.5	21.7	26.4
$CHBr_2I$	95.7	123.7	21.0	25.9
CHI_2F	88.7	122.1	19.8	25.6
CHI_2Cl	98.2	128.2	21.4	26.7
CHI_2Br	95.4	126.1	21.0	26.3
CHBrClF	101.5	129.5	22.0	26.9
CHIClF	96.7	121.9	21.2	25.6
CHIBrF	91.0	119.8	20.2	25.2
CHIBrCl	98.7	124.5	21.5	26.1

Table S24. Water: Relative Solution Acidities (ΔG_{298}) in kJ/mol and pK_a Values at the MP2/aT Level.

Species	ΔG_{298}	ΔG_{298}	pK_a	pK_a
Implicit	COSMO	SMD	COSMO	SMD
CH_4	262.8	265.5	50.3	50.8
CH_3F	253.4	259.6	48.7	49.7
CH_2F_2	225.7	235.5	43.8	45.5
CHF_3	157.6	169.5	31.9	33.9
CH_3Cl	224.5	237.6	43.6	45.9
CH_2Cl_2	176.4	194.3	35.2	38.3
$CHCl_3$	120.9	138.0	25.4	28.4
CH_3Br	220.1	246.0	42.8	47.3
CH_2Br_2	168.0	201.2	33.7	39.5
$CHBr_3$	114.7	145.4	24.3	29.7
CH_3I	213.3	226.3	41.6	43.9
CH_2I_2	159.7	184.2	32.2	36.5
CHI_3	104.3	139.5	22.5	28.7
CH_2FCl	197.8	213.4	38.9	41.6
CH_2FBr	192.4	218.2	37.9	42.5
CH_2FI	187.1	205.4	37.0	40.2
CH_2ClBr	172.9	198.5	34.5	39.0
CH_2ClI	169.1	189.5	33.9	37.4
CH_2BrI	165.7	191.8	33.3	37.9
CHF_2Cl	136.6	155.1	28.2	31.4
CHF_2Br	126.8	159.0	26.5	32.1
CHF_2I	111.1	135.6	23.7	28.0
$CHCl_2F$	129.1	146.7	26.9	30.0
$CHCl_2Br$	118.7	140.6	25.0	28.9
$CHCl_2I$	117.6	139.8	24.8	28.7
$CHBr_2F$	121.3	151.3	25.5	30.8
$CHBr_2Cl$	116.7	143.3	24.7	29.4
$CHBr_2I$	112.9	141.4	24.0	29.0
CHI_2F	115.6	146.7	24.5	29.9
CHI_2Cl	113.0	141.4	24.0	29.0
CHI_2Br	110.2	140.5	23.6	28.9
CHBrClF	125.0	149.1	26.2	30.4
CHIClF	122.7	146.3	25.7	29.9
CHIBrF	118.7	146.1	25.0	29.8
CHIBrCl	115.1	140.4	24.4	28.9

Table S25. Water: Relative Solution Acidities (ΔG_{298}) in kJ/mol and pK_a Values at the G3(MP2) Level.

Species	ΔG_{298}	ΔG_{298}	pK_a	pK_a
Implicit	COSMO	SMD	COSMO	SMD
CH_4	267.5	270.1	51.1	51.6
CH_3F	249.7	255.8	48.0	49.1
CH_2F_2	217.7	227.5	42.4	44.1
CHF_3	150.7	162.6	30.7	32.7
CH_3Cl	211.8	224.9	41.4	43.6
CH_2Cl_2	158.5	176.4	32.0	35.2
$CHCl_3$	100.1	117.2	21.8	24.8
CH_3Br	203.6	229.5	39.9	44.4
CH_2Br_2	143.4	176.6	29.4	35.2
$CHBr_3$	88.0	118.7	19.7	25.0
CH_3I	199.1	212.1	39.1	41.4
CH_2I_2	137.8	162.3	28.4	32.7
CHI_3	82.5	117.7	18.7	24.9
CH_2FCl	179.2	194.8	35.6	38.4
CH_2FBr	168.7	194.6	33.8	38.3
CH_2FI	166.6	185.0	33.4	36.7
CH_2ClBr	151.4	176.9	30.8	35.2
CH_2ClI	147.7	168.1	30.1	33.7
CH_2BrI	142.1	168.1	29.1	33.7
CHF_2Cl	119.9	138.5	25.3	28.5
CHF_2Br	100.1	132.4	21.8	27.4
CHF_2I	83.9	108.4	19.0	23.2
$CHCl_2F$	108.2	125.8	23.2	26.3
$CHCl_2Br$	94.8	116.6	20.9	24.7
$CHCl_2I$	92.4	114.7	20.4	24.3
$CHBr_2F$	90.7	120.7	20.1	25.4
$CHBr_2Cl$	89.9	116.5	20.0	24.7
$CHBr_2I$	84.2	112.7	19.0	24.0
CHI_2F	87.6	118.6	19.6	25.0
CHI_2Cl	87.4	115.9	19.6	24.5
CHI_2Br	83.3	113.5	18.8	24.1
CHBrClF	98.2	122.3	21.5	25.7
CHIClF	96.5	120.1	21.2	25.3
CHIBrF	88.9	116.4	19.8	24.6
CHIBrCl	88.3	113.6	19.7	24.2

Table S26. DMSO: Relative Solution Acidities (ΔG_{298}) in kJ/mol and pK_a Values at the B3LYP/aT Level.

Species	ΔG_{298}	ΔG_{298}	pK_a	pK_a
Implicit	COSMO	SMD	COSMO	SMD
CH_4	270.2	236.4	51.6	45.7
CH_3F	251.4	221.3	48.3	43.0
CH_2F_2	218.0	191.3	42.4	37.8
CHF_3	148.7	123.7	30.3	25.9
CH_3Cl	222.6	200.3	43.2	39.3
CH_2Cl_2	167.5	150.0	33.6	30.5
$CHCl_3$	110.1	91.2	23.5	20.2
CH_3Br	216.0	206.8	42.1	40.5
CH_2Br_2	155.1	152.1	31.4	30.9
$CHBr_3$	95.3	87.8	20.9	19.6
CH_3I	211.2	188.7	41.3	37.3
CH_2I_2	150.2	139.4	30.6	28.7
CHI_3	94.0	93.5	20.7	20.6
CH_2FCl	187.0	167.9	37.0	33.7
CH_2FBr	177.7	170.4	35.4	34.1
CH_2FI	171.7	155.0	34.3	31.4
CH_2ClBr	161.9	151.9	32.6	30.9
CH_2ClI	159.1	143.9	32.1	29.5
CH_2BrI	154.3	144.3	31.3	29.5
CHF_2Cl	112.0	97.6	23.9	21.4
CHF_2Br	94.6	96.6	20.8	21.2
CHF_2I	81.6	70.0	18.5	16.5
$CHCl_2F$	109.8	92.8	23.5	20.5
$CHCl_2Br$	102.8	88.5	22.3	19.8
$CHCl_2I$	101.8	88.0	22.1	19.7
$CHBr_2F$	93.8	89.4	20.7	19.9
$CHBr_2Cl$	98.9	88.5	21.6	19.8
$CHBr_2I$	95.1	85.7	20.9	19.3
CHI_2F	88.0	84.4	19.7	19.0
CHI_2Cl	97.5	90.2	21.3	20.1
CHI_2Br	94.6	88.1	20.8	19.7
CHBrClF	101.1	91.5	22.0	20.3
CHIClF	96.1	84.3	21.1	19.0
CHIBrF	90.4	82.0	20.1	18.6
CHIBrCl	98.1	86.6	21.4	19.4

Table S27. DMSO: Relative Solution Acidities (ΔG_{298}) in kJ/mol and pK_a Values at the MP2/aT Level.

Species	ΔG_{298}	ΔG_{298}	pK_a	pK_a
Implicit	COSMO	SMD	COSMO	SMD
CH_4	261.5	232.2	54.4	47.5
CH_3F	252.0	226.0	52.7	46.4
CH_2F_2	224.0	201.6	47.8	42.2
CHF_3	155.8	135.4	35.9	30.6
CH_3Cl	222.8	203.7	47.6	42.6
CH_2Cl_2	174.4	160.0	39.1	34.9
$CHCl_3$	118.7	103.5	29.4	25.0
CH_3Br	218.3	212.0	46.8	44.0
CH_2Br_2	165.9	166.8	37.6	36.1
$CHBr_3$	112.4	110.7	28.3	26.3
CH_3I	211.4	192.4	45.6	40.6
CH_2I_2	157.4	149.7	36.1	33.1
CHI_3	101.8	104.5	26.4	25.2
CH_2FCl	195.9	179.2	42.9	38.3
CH_2FBr	190.4	184.0	41.9	39.1
CH_2FI	185.0	171.2	41.0	36.8
CH_2ClBr	170.8	164.1	38.5	35.6
CH_2ClI	166.9	155.1	37.8	34.0
CH_2BrI	163.5	157.4	37.2	34.4
CHF_2Cl	134.6	120.7	32.1	28.0
CHF_2Br	124.7	124.3	30.4	28.6
CHF_2I	109.0	101.5	27.7	24.6
$CHCl_2F$	126.9	112.2	30.8	26.5
$CHCl_2Br$	116.5	106.0	29.0	25.4
$CHCl_2I$	115.3	105.2	28.8	25.3
$CHBr_2F$	119.1	116.6	29.4	27.3
$CHBr_2Cl$	114.5	108.7	28.6	25.9
$CHBr_2I$	110.5	106.6	27.9	25.5
CHI_2F	123.5	124.6	30.2	28.7
CHI_2Cl	110.6	106.6	27.9	25.5
CHI_2Br	107.8	105.5	27.5	25.4
CHBrClF	122.9	114.5	30.1	26.9
CHIClF	120.4	111.7	29.7	26.4
CHIBrF	116.4	111.4	28.9	26.4
CHIBrCl	112.8	105.7	28.3	25.4

Table S28. DMSO: Relative Solution Acidities (ΔG_{298}) in kJ/mol and pK_a Values at the G3(MP2) Level.

Species	ΔG_{298}	ΔG_{298}	pK_a	pK_a
Implicit	COSMO	SMD	COSMO	SMD
CH_4	266.2	236.8	57.7	52.6
CH_3F	248.2	222.2	54.6	50.0
CH_2F_2	216.1	193.6	49.0	45.0
CHF_3	148.9	128.5	37.2	33.6
CH_3Cl	210.1	191.0	47.9	44.6
CH_2Cl_2	156.5	142.2	38.5	36.0
$CHCl_3$	97.9	82.7	28.3	25.6
CH_3Br	201.8	195.5	46.4	45.3
CH_2Br_2	141.2	142.1	35.8	36.0
$CHBr_3$	85.7	84.0	26.1	25.8
CH_3I	197.2	178.2	45.6	42.3
CH_2I_2	135.5	127.8	34.8	33.5
CHI_3	80.0	82.7	25.1	25.6
CH_2FCl	177.3	160.6	42.2	39.2
CH_2FBr	166.8	160.4	40.3	39.2
CH_2FI	164.6	150.8	39.9	37.5
CH_2ClBr	149.3	142.6	37.3	36.1
CH_2ClI	145.6	133.7	36.6	34.5
CH_2BrI	139.9	133.7	35.6	34.5
CHF_2Cl	117.9	104.0	31.8	29.3
CHF_2Br	98.0	97.7	28.3	28.2
CHF_2I	81.8	74.3	25.4	24.1
$CHCl_2F$	106.1	91.3	29.7	27.1
$CHCl_2Br$	92.6	82.1	27.3	25.5
$CHCl_2I$	90.1	80.0	26.9	25.1
$CHBr_2F$	88.5	86.0	26.6	26.2
$CHBr_2Cl$	87.7	81.9	26.5	25.5
$CHBr_2I$	81.8	77.9	25.4	24.7
CHI_2F	95.4	96.5	27.8	28.0
CHI_2Cl	85.0	81.0	26.0	25.3
CHI_2Br	80.9	78.6	25.3	24.9
CHBrClF	96.1	87.7	27.9	26.5
CHIClF	94.2	85.5	27.6	26.1
CHIBrF	86.6	81.6	26.3	25.4
CHIBrCl	85.9	78.9	26.2	24.9

Table S29. MeCN: Relative Solution Acidities (ΔG_{298}) in kJ/mol and pK_a Values at the B3LYP/aT Level.

Species	ΔG_{298}	ΔG_{298}	pK_a	pK_a
Implicit	COSMO	SMD	COSMO	SMD
CH_4	270.5	240.5	51.6	46.4
CH_3F	251.5	225.0	48.3	43.7
CH_2F_2	218.0	194.8	42.4	38.4
CHF_3	148.6	126.9	30.3	26.5
CH_3Cl	222.6	203.8	43.2	39.9
CH_2Cl_2	167.3	153.0	33.6	31.1
$CHCl_3$	109.8	94.1	23.5	20.7
CH_3Br	216.0	210.1	42.1	41.1
CH_2Br_2	154.8	154.9	31.4	31.4
$CHBr_3$	94.8	90.5	20.9	20.1
CH_3I	211.1	192.0	41.2	37.9
CH_2I_2	149.7	142.2	30.5	29.2
CHI_3	93.4	96.0	20.6	21.1
CH_2FCl	186.9	171.0	37.0	34.2
CH_2FBr	177.6	173.3	35.4	34.6
CH_2FI	171.4	157.9	34.3	31.9
CH_2ClBr	161.6	154.9	32.6	31.4
CH_2ClI	158.7	146.7	32.1	30.0
CH_2BrI	153.9	147.1	31.2	30.0
CHF_2Cl	111.9	100.4	23.8	21.8
CHF_2Br	94.4	99.2	20.8	21.6
CHF_2I	81.3	72.6	18.5	17.0
$CHCl_2F$	109.5	95.6	23.4	21.0
$CHCl_2Br$	102.4	91.3	22.2	20.2
$CHCl_2I$	101.3	90.7	22.0	20.1
$CHBr_2F$	93.4	92.0	20.6	20.4
$CHBr_2Cl$	98.5	91.3	21.5	20.2
$CHBr_2I$	94.6	88.4	20.8	19.7
CHI_2F	87.5	86.8	19.6	19.5
CHI_2Cl	96.9	92.8	21.2	20.5
CHI_2Br	94.1	90.6	20.7	20.1
CHBrClF	100.7	94.2	21.9	20.8
CHIClF	95.7	86.8	21.0	19.5
CHIBrF	89.9	84.5	20.0	19.1
CHIBrCl	97.6	89.3	21.4	19.9

Table S30. MeCN: Relative Solution Acidities (ΔG_{298}) in kJ/mol and pK_a Values at the MP2/aT Level.

Species	ΔG_{298}	ΔG_{298}	pK_a	pK_a
Implicit	COSMO	SMD	COSMO	SMD
CH_4	263.6	233.7	67.7	62.5
CH_3F	253.9	227.2	44.5	39.8
CH_2F_2	225.8	202.5	39.6	35.5
CHF_3	157.5	136.1	27.6	23.8
CH_3Cl	224.6	204.7	39.3	35.9
CH_2Cl_2	175.9	160.6	30.8	28.1
$CHCl_3$	120.1	104.0	21.0	18.2
CH_3Br	220.0	212.9	38.5	37.3
CH_2Br_2	167.3	167.2	29.3	29.3
$CHBr_3$	113.7	111.0	19.9	19.4
CH_3I	213.1	193.3	37.3	33.9
CH_2I_2	158.7	150.1	27.8	26.3
CHI_3	103.0	104.6	18.0	18.3
CH_2FCl	197.5	179.9	34.6	31.5
CH_2FBr	192.0	184.6	33.6	32.3
CH_2FI	186.5	171.7	32.7	30.1
CH_2ClBr	172.3	164.6	30.2	28.8
CH_2ClI	168.4	155.6	29.5	27.3
CH_2BrI	164.9	157.8	28.9	27.6
CHF_2Cl	136.1	121.1	23.8	21.2
CHF_2Br	126.2	124.6	22.1	21.8
CHF_2I	110.4	101.6	19.3	17.8
$CHCl_2F$	128.4	112.7	22.5	19.7
$CHCl_2Br$	117.9	106.4	20.7	18.6
$CHCl_2I$	116.6	105.5	20.4	18.5
$CHBr_2F$	120.5	116.9	21.1	20.5
$CHBr_2Cl$	115.8	109.1	20.3	19.1
$CHBr_2I$	111.8	106.9	19.6	18.7
CHI_2F	124.7	124.4	21.8	21.8
CHI_2Cl	111.8	106.8	19.6	18.7
CHI_2Br	109.1	105.7	19.1	18.5
CHBrClF	124.3	114.8	21.8	20.1
CHIClF	121.8	111.9	21.3	19.6
CHIBrF	117.7	111.6	20.6	19.6
CHIBrCl	114.1	106.0	20.0	18.6

Table S31. MeCN: Relative Solution Acidities (ΔG_{298}) in kJ/mol and pK_a Values at the G3(MP2) Level.

Species	ΔG_{298}	ΔG_{298}	pK_a	pK_a
Implicit	COSMO	SMD	COSMO	SMD
CH_4	268.3	238.4	68.5	63.3
CH_3F	263.6	236.9	67.7	63.0
CH_2F_2	231.4	208.1	62.0	58.0
CHF_3	164.1	142.7	50.3	46.5
CH_3Cl	225.4	205.5	61.0	57.5
CH_2Cl_2	171.6	156.2	51.6	48.9
$CHCl_3$	112.8	96.7	41.3	38.4
CH_3Br	217.0	209.9	59.5	58.3
CH_2Br_2	156.2	156.1	48.9	48.8
$CHBr_3$	100.5	97.8	39.1	38.6
CH_3I	212.3	192.5	58.7	55.2
CH_2I_2	150.3	141.7	47.8	46.3
CHI_3	94.7	96.3	38.1	38.4
CH_2FCl	192.4	174.8	55.2	52.1
CH_2FBr	181.8	174.4	53.4	52.1
CH_2FI	179.6	164.7	53.0	50.4
CH_2ClBr	164.3	156.6	50.3	48.9
CH_2ClI	160.5	147.7	49.6	47.4
CH_2BrI	154.8	147.6	48.6	47.4
CHF_2Cl	132.9	117.9	44.8	42.2
CHF_2Br	113.0	111.4	41.3	41.0
CHF_2I	96.7	87.9	38.4	36.9
$CHCl_2F$	121.0	105.2	42.7	39.9
$CHCl_2Br$	107.4	96.0	40.3	38.3
$CHCl_2I$	105.0	93.8	39.9	37.9
$CHBr_2F$	103.3	99.7	39.6	39.0
$CHBr_2Cl$	102.5	95.7	39.5	38.3
$CHBr_2I$	96.6	91.6	38.4	37.5
CHI_2F	110.1	109.8	40.8	40.7
CHI_2Cl	99.8	94.7	39.0	38.1
CHI_2Br	95.6	92.2	38.2	37.7
CHBrClF	111.0	101.5	40.9	39.3
CHIClF	109.1	99.1	40.6	38.9
CHIBrF	101.4	95.3	39.3	38.2
CHIBrCl	100.7	92.6	39.1	37.7

Table S32. THF: Relative Solution Acidities (ΔG_{298}) in kJ/mol and pK_a Values at the B3LYP/aT Level.

Species	ΔG_{298}	ΔG_{298}	pK_a	pK_a
Implicit	COSMO	SMD	COSMO	SMD
CH_4	276.2	248.5	52.6	47.8
CH_3F	254.9	229.5	48.9	44.5
CH_2F_2	219.2	195.9	42.7	38.6
CHF_3	148.1	125.4	30.2	26.2
CH_3Cl	222.9	204.5	43.3	40.1
CH_2Cl_2	164.0	148.8	33.0	30.3
$CHCl_3$	104.4	87.3	22.5	19.5
CH_3Br	215.3	208.9	42.0	40.8
CH_2Br_2	150.0	147.9	30.5	30.2
$CHBr_3$	87.8	81.3	19.6	18.5
CH_3I	208.9	190.5	40.9	37.6
CH_2I_2	143.0	134.2	29.3	27.8
CHI_3	84.2	83.7	19.0	18.9
CH_2FCl	185.3	168.5	36.7	33.8
CH_2FBr	174.8	168.8	34.9	33.8
CH_2FI	167.2	152.8	33.5	31.0
CH_2ClBr	157.5	149.2	31.8	30.4
CH_2ClI	153.5	140.4	31.1	28.9
CH_2BrI	148.1	139.9	30.2	28.8
CHF_2Cl	109.4	95.7	23.4	21.0
CHF_2Br	91.1	91.8	20.2	20.3
CHF_2I	76.6	66.3	17.7	15.9
$CHCl_2F$	105.0	89.4	22.6	19.9
$CHCl_2Br$	96.4	83.8	21.1	18.9
$CHCl_2I$	94.2	81.7	20.8	18.6
$CHBr_2F$	87.4	83.0	19.6	18.8
$CHBr_2Cl$	91.9	82.8	20.4	18.8
$CHBr_2I$	86.7	78.4	19.4	18.0
CHI_2F	79.3	75.5	18.1	17.5
CHI_2Cl	88.6	81.9	19.8	18.6
CHI_2Br	85.5	79.3	19.2	18.1
CHBrClF	95.4	86.4	21.0	19.4
CHIClF	89.1	78.1	19.9	17.9
CHIBrF	82.8	74.9	18.7	17.4
CHIBrCl	90.1	79.7	20.0	18.2

Table S33. THF: Relative Solution Acidities (ΔG_{298}) in kJ/mol and pK_a Values at the MP2/aT Level.

Species	ΔG_{298}	ΔG_{298}	pK_a	pK_a
Implicit	COSMO	SMD	COSMO	SMD
CH_4	269.4	241.9	72.3	67.5
CH_3F	257.4	231.9	70.2	65.7
CH_2F_2	227.2	203.9	64.9	60.8
CHF_3	157.2	134.9	52.6	48.7
CH_3Cl	225.3	206.2	64.6	61.2
CH_2Cl_2	173.0	157.3	55.4	52.7
$CHCl_3$	115.2	98.3	45.3	42.3
CH_3Br	219.7	212.6	63.6	62.4
CH_2Br_2	163.1	161.3	53.7	53.4
$CHBr_3$	107.3	102.5	43.9	43.1
CH_3I	211.4	192.5	62.1	58.8
CH_2I_2	152.5	143.0	51.8	50.2
CHI_3	94.3	93.4	41.6	41.5
CH_2FCl	196.3	178.2	59.5	56.3
CH_2FBr	189.7	181.3	58.3	56.9
CH_2FI	182.8	167.3	57.1	54.4
CH_2ClBr	168.7	159.9	54.7	53.1
CH_2ClI	163.7	150.2	53.8	51.4
CH_2BrI	159.7	151.4	53.1	51.6
CHF_2Cl	133.4	116.8	48.5	45.6
CHF_2Br	122.4	117.9	46.6	45.8
CHF_2I	105.9	95.3	43.7	41.8
$CHCl_2F$	124.3	107.4	46.9	43.9
$CHCl_2Br$	112.4	99.9	44.8	42.6
$CHCl_2I$	110.2	97.5	44.4	42.2
$CHBr_2F$	114.9	109.3	45.2	44.3
$CHBr_2Cl$	109.9	101.5	44.4	42.9
$CHBr_2I$	104.5	97.5	43.4	42.2
CHI_2F	116.6	113.3	45.5	45.0
CHI_2Cl	104.2	96.9	43.4	42.1
CHI_2Br	101.0	95.3	42.8	41.8
CHBrClF	119.4	108.4	46.0	44.1
CHIClF	115.6	103.7	45.4	43.3
CHIBrF	111.0	102.7	44.5	43.1
CHIBrCl	107.2	97.4	43.9	42.2

Table S34. THF: Relative Solution Acidities (ΔG_{298}) in kJ/mol and pK_a Values at the G3(MP2) Level.

Species	ΔG_{298}	ΔG_{298}	pK_a	pK_a
Implicit	COSMO	SMD	COSMO	SMD
CH_4	274.1	246.5	73.1	68.3
CH_3F	253.6	228.1	69.5	65.1
CH_2F_2	219.2	196.0	63.5	59.4
CHF_3	150.3	128.0	51.4	47.5
CH_3Cl	212.6	193.4	62.4	59.0
CH_2Cl_2	155.2	139.4	52.3	49.5
$CHCl_3$	94.5	77.6	41.7	38.7
CH_3Br	203.2	196.1	60.7	59.5
CH_2Br_2	138.4	136.6	49.4	49.0
$CHBr_3$	80.6	75.8	39.2	38.4
CH_3I	197.2	178.3	59.7	56.3
CH_2I_2	130.6	121.1	48.0	46.3
CHI_3	72.5	71.7	37.8	37.7
CH_2FCl	177.7	159.6	56.2	53.1
CH_2FBr	166.1	157.7	54.2	52.7
CH_2FI	162.3	146.9	53.6	50.8
CH_2ClBr	147.2	138.4	50.9	49.3
CH_2ClI	142.3	128.8	50.0	47.7
CH_2BrI	136.0	127.8	48.9	47.5
CHF_2Cl	116.7	100.2	45.6	42.7
CHF_2Br	95.8	91.3	41.9	41.1
CHF_2I	78.7	68.2	38.9	37.1
$CHCl_2F$	103.4	86.5	43.2	40.3
$CHCl_2Br$	88.5	76.0	40.6	38.4
$CHCl_2I$	85.1	72.4	40.0	37.8
$CHBr_2F$	84.3	78.7	39.9	38.9
$CHBr_2Cl$	83.1	74.7	39.7	38.2
$CHBr_2I$	75.8	68.8	38.4	37.2
CHI_2F	88.5	85.2	40.6	40.0
CHI_2Cl	78.6	71.4	38.9	37.6
CHI_2Br	74.1	68.4	38.1	37.1
CHBrClF	92.6	81.6	41.3	39.4
CHIClF	89.4	77.5	40.8	38.7
CHIBrF	81.2	73.0	39.3	37.9
CHIBrCl	80.4	70.6	39.2	37.5

Table S35. T1 Values (Multireference Diagnostic) for Radical Species using CCSD(T)/aug-cc-pV5Z(-PP).

Species	a5(-PP) T1
$CH_3^\bullet$	0.006
$CH_2F^\bullet$	0.015
$CHF_2^\bullet$	0.016
$CF_3^\bullet$	0.015
$CH_2Cl^\bullet$	0.016
$CHCl_2^\bullet$	0.017
$CCl_3^\bullet$	0.016
$CH_2Br^\bullet$	0.019
$CHBr_2^\bullet$	0.019
$CBr_3^\bullet$	0.018
$CH_2I^\bullet$	0.022
$CHI_2^\bullet$	0.023
$CI_3^\bullet$	0.022
$CHFCl^\bullet$	0.017
$CHFBr^\bullet$	0.018
$CHFI^\bullet$	0.020
$CHClBr^\bullet$	0.018
$CHClI^\bullet$	0.020
$CHBrI^\bullet$	0.021
$CF_2Cl^\bullet$	0.016
$CF_2Br^\bullet$	0.017
$CF_2I^\bullet$	0.018
$CCl_2F^\bullet$	0.016
$CCl_2Br^\bullet$	0.017
$CCl_2I^\bullet$	0.018
$CBr_2F^\bullet$	0.018
$CBr_2Cl^\bullet$	0.018
$CBr_2I^\bullet$	0.020
$CI_2F^\bullet$	0.021
$CI_2Cl^\bullet$	0.020
$CI_2Br^\bullet$	0.021
$CBrClF^\bullet$	0.017
$CIClF^\bullet$	0.018
$CIBrF^\bullet$	0.019
$CIBrCl^\bullet$	0.019

Table S36. Gas Phase Total Energies for Neutral Species in a.u.

Species	B3LYP/aT	MP2/aT
Benzoic Acid	-420.988753	-420.062388
CH_4	-40.538425	-40.414459
CH_3F	-139.804430	-139.541532
CH_2F_2	-239.088401	-238.688661
CHF_3	-338.381371	-337.846364
CH_3Cl	-500.165806	-499.529249
CH_2Cl_2	-959.792070	-958.645088
$CHCl_3$	-1419.413616	-1417.758762
CH_3Br	-456.852461	-455.558833
CH_2Br_2	-873.163502	-870.703296
$CHBr_3$	-1289.469389	-1285.845993
CH_3I	-335.737094	-334.696672
CH_2I_2	-630.930843	-628.977992
CHI_3	-926.119152	-923.258094
CH_2FCl	-599.438126	-598.663370
CH_2FBr	-556.122749	-554.690453
CH_2FI	-435.004575	-433.824608
CH_2ClBr	-916.477755	-914.673960
CH_2ClI	-795.361045	-793.810536
CH_2BrI	-752.047003	-749.840286
CHF_2Cl	-698.721792	-697.810744
CHF_2Br	-655.405112	-653.836195
CHF_2I	-534.285462	-532.968156
$CHCl_2F$	-1059.065785	-1057.781472
$CHCl_2Br$	-1376.098835	-1373.787430
$CHCl_2I$	-1254.981636	-1252.923602
$CHBr_2F$	-972.434293	-969.835902
$CHBr_2Cl$	-1332.784087	-1329.816512
$CHBr_2I$	-1168.352459	-1164.982805
CHI_2F	-730.197806	-728.105201
CHI_2Cl	-1090.550100	-1088.090056
CHI_2Br	-1047.235703	-1044.120161
CHBrClF	-1015.750023	-1013.808502
CHIClF	-894.631576	-892.942573
CHIBrF	-851.315973	-848.970288
CHIBrCl	-1211.667017	-1208.953018

Table S37. Gas Phase Total Energies for Radical Species in a.u.

Species	B3LYP/aT	MP2/aT
$CH_3^{\bullet}$	-39.859409	-39.740296
$CH_2F^{\bullet}$	-139.132756	-138.872751
$CHF_2^{\bullet}$	-238.417557	-238.019203
$CF_3^{\bullet}$	-337.703562	-337.169204
$CH_2Cl^{\bullet}$	-499.496309	-498.862135
$CHCl_2^{\bullet}$	-959.129508	-957.982744
$CCl_3^{\bullet}$	-1418.756892	-1417.100340
$CH_2Br^{\bullet}$	-456.181131	-454.889468
$CHBr_2^{\bullet}$	-872.499448	-870.038589
$CBr_3^{\bullet}$	-1288.813177	-1285.186602
$CH_2I^{\bullet}$	-335.065082	-334.026558
$CHI_2^{\bullet}$	-630.267575	-628.314041
$CI_3^{\bullet}$	-925.466013	-922.601885
$CHFCl^{\bullet}$	-598.771047	-597.997139
$CHFBr^{\bullet}$	-555.455089	-554.023216
$CHFI^{\bullet}$	-434.337469	-433.157934
$CHClBr^{\bullet}$	-915.814410	-914.010460
$CHClI^{\bullet}$	-794.698067	-793.147485
$CHBrI^{\bullet}$	-751.383342	-749.176021
$CF_2Cl^{\bullet}$	-698.051857	-697.140623
$CF_2Br^{\bullet}$	-654.737099	-653.167246
$CF_2I^{\bullet}$	-533.620887	-532.302469
$CCl_2F^{\bullet}$	-1058.402523	-1057.117229
$CCl_2Br^{\bullet}$	-1375.442308	-1373.128798
$CCl_2I^{\bullet}$	-1254.326511	-1252.266507
$CBr_2F^{\bullet}$	-971.772750	-969.172086
$CBr_2Cl^{\bullet}$	-1332.127743	-1329.157553
$CBr_2I^{\bullet}$	-1167.697443	-1164.324700
$CI_2F^{\bullet}$	-729.539920	-727.444625
$CI_2Cl^{\bullet}$	-1089.896187	-1087.433627
$CI_2Br^{\bullet}$	-1046.581707	-1043.463104
$CBrClF^{\bullet}$	-1015.087646	-1013.144561
$CIClF^{\bullet}$	-893.971366	-892.280713
$CIBrF^{\bullet}$	-850.656414	-848.308306
$CIBrCl^{\bullet}$	-1211.011976	-1208.295459

Table S38. Gas Phase Total Energies for Anion Species in a.u.

Species	B3LYP/aT	MP2/aT
Benzoate	-420.435381	-419.513170
CH_3^-	-39.862397	-39.740230
CH_2F^-	-139.140668	-138.875832
CHF_2^-	-238.443420	-238.039860
CF_3^-	-337.767646	-337.228346
CH_2Cl^-	-499.522530	-498.884245
$CHCl_2^-$	-959.182550	-958.031274
CCl_3^-	-1418.833913	-1417.172961
CH_2Br^-	-456.215599	-454.919285
$CHBr_2^-$	-872.563920	-870.097614
CBr_3^-	-1288.901096	-1285.269095
CH_2I^-	-335.107598	-334.065389
CHI_2^-	-630.340785	-628.383437
CI_3^-	-925.559962	-922.693331
$CHFCl^-$	-598.814503	-598.034724
$CHFBr^-$	-555.506821	-554.067775
$CHFI^-$	-434.396629	-433.209705
$CHClBr^-$	-915.873468	-914.064407
$CHClI^-$	-794.762338	-793.206931
$CHBrI^-$	-751.452451	-749.240311
CF_2Cl^-	-698.129147	-697.209366
CF_2Br^-	-654.822501	-653.242204
CF_2I^-	-533.712900	-532.383583
CCl_2F^-	-1058.481290	-1057.188782
CCl_2Br^-	-1375.523432	-1373.204887
CCl_2I^-	-1254.410623	-1252.345373
CBr_2F^-	-971.861898	-969.252065
CBr_2Cl^-	-1332.212479	-1329.236929
CBr_2I^-	-1167.787567	-1164.410023
CI_2F^-	-729.635526	-727.531745
CI_2Cl^-	-1089.985759	-1087.518765
CI_2Br^-	-1046.673820	-1043.551425
$CBrClF^-$	-1015.172004	-1013.220459
$CIClF^-$	-894.059996	-892.360214
$CIBrF^-$	-850.749095	-848.391807
$CIBrCl^-$	-1211.099271	-1208.377625

Table S39. Gas Phase Enthalpy at 0 K using G3MP2 in a.u.

Species	Neutrals	Radicals	Anions
Benzoic Acid	-420.200783		-419.660364
CH_4	-40.422085	-39.757116	-39.757340
CH_3F	-139.569865	-138.909474	-138.916929
CH_2F_2	-238.738275	-238.077043	-238.103717
CHF_3	-337.918244	-337.248813	-337.314028
CH_3Cl	-499.573650	-498.916049	-498.943490
CH_2Cl_2	-958.727861	-958.075247	-958.130278
$CHCl_3$	-1417.881255	-1417.232641	-1417.312780
CH_3Br	-2612.649708	-2611.990164	-2612.026022
CH_2Br_2	-5184.877746	-5184.223540	-5184.290258
$CHBr_3$	-7757.104197	-7756.455911	-7756.547066
CH_3I	-335.226232	-334.566296	-334.609514
CH_2I_2	-630.030127	-629.377159	-629.452097
CHI_3	-924.833527	-924.188755	-924.285816
CH_2FCl	-598.729673	-598.072303	-598.117285
CH_2FBr	-2711.803040	-2711.144988	-2711.197683
CH_2FI	-434.376410	-433.719066	-433.777314
CH_2ClBr	-3071.802709	-3071.149282	-3071.210352
CH_2ClI	-794.378351	-793.725799	-793.791383
CH_2BrI	-2907.454615	-2906.801179	-2906.872119
CHF_2Cl	-697.899805	-697.238054	-697.313696
CHF_2Br	-2810.971347	-2810.311172	-2810.395688
CHF_2I	-533.542882	-532.886017	-532.977258
$CHCl_2F$	-1057.887524	-1057.232385	-1057.312113
$CHCl_2Br$	-3530.955455	-3530.306983	-3530.391041
$CHCl_2I$	-1253.531038	-1252.884473	-1252.970819
$CHBr_2F$	-5284.033268	-5283.379385	-5283.468987
$CHBr_2Cl$	-5644.029769	-5643.381422	-5643.469146
$CHBr_2I$	-5479.682113	-5479.035352	-5479.128547
CHI_2F	-729.181206	-728.530790	-728.625925
CHI_2Cl	-1089.181601	-1088.536130	-1088.628179
CHI_2Br	-3202.257538	-3201.611889	-3201.707054
CHBrClF	-3170.960342	-3170.305875	-3170.390298
CHIClF	-893.533880	-892.881734	-892.968944
CHIBrF	-3006.607933	-3005.956066	-3006.048281
CHIBrCl	-3366.606531	-3365.959883	-3366.049778

Table S40. B3LYP/aT/SMD Total Energies for Neutral Species in a.u.

Species	Water	DMSO	MeCN	THF
CH_4	-40.537657	-40.536557	-40.537766	-40.537657
CH_3F	-139.807165	-139.806778	-139.807946	-139.807165
CH_2F_2	-239.092347	-239.092332	-239.093480	-239.092347
CHF_3	-338.384034	-338.383888	-338.385045	-338.384034
CH_3Cl	-500.170308	-500.169776	-500.170930	-500.170308
CH_2Cl_2	-959.799135	-959.798818	-959.799944	-959.799135
$CHCl_3$	-1419.420591	-1419.419852	-1419.421022	-1419.420591
CH_3Br	-456.857279	-456.856513	-456.857659	-456.857279
CH_2Br_2	-873.171358	-873.170562	-873.171673	-873.171358
$CHBr_3$	-1289.478475	-1289.477166	-1289.478314	-1289.478475
CH_3I	-335.738554	-335.737737	-335.739155	-335.738554
CH_2I_2	-630.933875	-630.933296	-630.934918	-630.933875
CHI_3	-926.123272	-926.122859	-926.124715	-926.123272
CH_2FCl	-599.443717	-599.443571	-599.444707	-599.443717
CH_2FBr	-556.128601	-556.128200	-556.129332	-556.128601
CH_2FI	-435.008111	-435.007830	-435.009223	-435.008111
CH_2ClBr	-916.485133	-916.484546	-916.485668	-916.485133
CH_2ClI	-795.366327	-795.365925	-795.367300	-795.366327
CH_2BrI	-752.052785	-752.052194	-752.053555	-752.052785
CHF_2Cl	-698.725912	-698.725576	-698.726735	-698.725912
CHF_2Br	-655.409756	-655.409186	-655.410340	-655.409756
CHF_2I	-534.288764	-534.288564	-534.289963	-534.288764
$CHCl_2F$	-1059.071365	-1059.070832	-1059.071995	-1059.071365
$CHCl_2Br$	-1376.106416	-1376.105479	-1376.106641	-1376.106416
$CHCl_2I$	-1254.988090	-1254.987615	-1254.989007	-1254.988090
$CHBr_2F$	-972.441101	-972.440148	-972.441297	-972.441101
$CHBr_2Cl$	-1332.792387	-1332.791252	-1332.792408	-1332.792387
$CHBr_2I$	-1168.360289	-1168.359445	-1168.360819	-1168.360289
CHI_2F	-730.201651	-730.201374	-730.203003	-730.201651
CHI_2Cl	-1090.555637	-1090.555275	-1090.556896	-1090.555637
CHI_2Br	-1047.241977	-1047.241482	-1047.243088	-1047.241977
CHBrClF	-1015.756141	-1015.755385	-1015.756542	-1015.756141
CHIClF	-894.636496	-894.636144	-894.637539	-894.636496
CHIBrF	-851.321490	-851.320948	-851.322333	-851.321490
CHIBrCl	-1211.674064	-1211.673367	-1211.674753	-1211.674064

Table S41. B3LYP/aT/COSMO Total Energies for Neutral Species in a.u.

Species	Water	DMSO	MeCN	THF
CH_4	-40.538956	-40.539029	-40.539024	-40.538956
CH_3F	-139.808417	-139.809026	-139.808990	-139.808417
CH_2F_2	-239.093639	-239.094444	-239.094396	-239.093639
CHF_3	-338.385667	-338.386323	-338.386284	-338.385667
CH_3Cl	-500.169651	-500.170262	-500.170225	-500.169651
CH_2Cl_2	-959.796846	-959.797603	-959.797557	-959.796846
$CHCl_3$	-1419.417456	-1419.418046	-1419.418011	-1419.417456
CH_3Br	-456.856096	-456.856659	-456.856624	-456.856096
CH_2Br_2	-873.168109	-873.168831	-873.168787	-873.168109
$CHBr_3$	-1289.473457	-1289.474069	-1289.474032	-1289.473457
CH_3I	-335.740466	-335.740995	-335.740963	-335.740466
CH_2I_2	-630.935375	-630.936076	-630.936033	-630.935375
CHI_3	-926.123742	-926.124426	-926.124385	-926.123742
CH_2FCl	-599.443173	-599.443963	-599.443915	-599.443173
CH_2FBr	-556.127735	-556.128514	-556.128467	-556.127735
CH_2FI	-435.009503	-435.010271	-435.010225	-435.009503
CH_2ClBr	-916.482442	-916.483180	-916.483135	-916.482442
CH_2ClI	-795.365688	-795.366416	-795.366373	-795.365688
CH_2BrI	-752.051571	-752.052283	-752.052240	-752.051571
CHF_2Cl	-698.725926	-698.726562	-698.726524	-698.725926
CHF_2Br	-655.409329	-655.409976	-655.409937	-655.409329
CHF_2I	-534.289839	-534.290510	-534.290469	-534.289839
$CHCl_2F$	-1059.069764	-1059.070378	-1059.070341	-1059.069764
$CHCl_2Br$	-1376.102762	-1376.103362	-1376.103326	-1376.102762
$CHCl_2I$	-1254.985764	-1254.986395	-1254.986357	-1254.985764
$CHBr_2F$	-972.438443	-972.439077	-972.439039	-972.438443
$CHBr_2Cl$	-1332.788132	-1332.788746	-1332.788709	-1332.788132
$CHBr_2I$	-1168.356762	-1168.357411	-1168.357372	-1168.356762
CHI_2F	-730.202278	-730.202957	-730.202916	-730.202278
CHI_2Cl	-1090.554495	-1090.555160	-1090.555120	-1090.554495
CHI_2Br	-1047.240171	-1047.240841	-1047.240801	-1047.240171
CHBrClF	-1015.754083	-1015.754706	-1015.754668	-1015.754083
CHIClF	-894.635799	-894.636446	-894.636408	-894.635799
CHIBrF	-851.320273	-851.320928	-851.320889	-851.320273
CHIBrCl	-1211.671212	-1211.671847	-1211.671809	-1211.671212

Table S42. MP2/aT/SMD Total Energies for Neutral Species in a.u.

Species	Water	DMSO	MeCN	THF
CH_4	-40.411058	-40.412671	-40.413878	-40.413755
CH_3F	-139.542103	-139.543912	-139.545080	-139.544290
CH_2F_2	-238.690612	-238.692608	-238.693755	-238.692619
CHF_3	-337.846587	-337.848792	-337.849948	-337.848957
CH_3Cl	-499.530421	-499.533022	-499.534172	-499.533588
CH_2Cl_2	-958.648090	-958.651622	-958.652744	-958.651975
$CHCl_3$	-1417.760439	-1417.764852	-1417.766015	-1417.765608
CH_3Br	-455.560130	-455.562738	-455.563874	-455.563522
CH_2Br_2	-870.706635	-870.710191	-870.711290	-870.711009
$CHBr_3$	-1285.849239	-1285.853673	-1285.854805	-1285.854974
CH_3I	-334.697916	-334.697144	-334.698551	-334.697995
CH_2I_2	-628.983153	-628.980148	-628.981755	-628.980792
CHI_3	-923.266274	-923.261238	-923.263073	-923.261766
CH_2FCl	-598.665914	-598.668689	-598.669822	-598.668858
CH_2FBr	-554.693000	-554.695790	-554.696913	-554.696209
CH_2FI	-433.828295	-433.827737	-433.829119	-433.828043
CH_2ClBr	-914.677022	-914.680568	-914.681681	-914.681179
CH_2ClI	-793.814941	-793.815177	-793.816540	-793.815623
CH_2BrI	-749.845006	-749.845264	-749.846611	-749.845892
CHF_2Cl	-697.811432	-697.814403	-697.815557	-697.814759
CHF_2Br	-653.837133	-653.840121	-653.841267	-653.840714
CHF_2I	-532.971432	-532.971096	-532.972484	-532.971329
$CHCl_2F$	-1057.782667	-1057.786375	-1057.787531	-1057.786932
$CHCl_2Br$	-1373.789526	-1373.793947	-1373.795098	-1373.794893
$CHCl_2I$	-1252.928137	-1252.929329	-1252.930709	-1252.929856
$CHBr_2F$	-969.837905	-969.841635	-969.842772	-969.842599
$CHBr_2Cl$	-1329.819116	-1329.823545	-1329.824687	-1329.824684
$CHBr_2I$	-1164.988381	-1164.989615	-1164.990971	-1164.990476
CHI_2F	-728.111250	-728.108495	-728.110107	-728.108831
CHI_2Cl	-1088.096822	-1088.094861	-1088.096464	-1088.095298
CHI_2Br	-1044.127550	-1044.125621	-1044.127207	-1044.126176
CHBrClF	-1013.810024	-1013.813744	-1013.814891	-1013.814517
CHIClF	-892.946493	-892.946937	-892.948320	-892.947330
CHIBrF	-848.974624	-848.975058	-848.976463	-848.975662
CHIBrCl	-1208.957950	-1208.959165	-1208.960535	-1208.959892

Table S43. MP2/aT/COSMO Total Energies for Neutral Species in a.u.

Species	Water	DMSO	MeCN	THF
CH_4	-40.415132	-40.415126	-40.415122	-40.415046
CH_3F	-139.546222	-139.546174	-139.546137	-139.545558
CH_2F_2	-238.694791	-238.694729	-238.694680	-238.693921
CHF_3	-337.851309	-337.851259	-337.851220	-337.850610
CH_3Cl	-499.533549	-499.533504	-499.533469	-499.532924
CH_2Cl_2	-958.650473	-958.650416	-958.650372	-958.649690
$CHCl_3$	-1417.763052	-1417.763008	-1417.762974	-1417.762443
CH_3Br	-455.562885	-455.562842	-455.562810	-455.562299
CH_2Br_2	-870.708482	-870.708428	-870.708386	-870.707735
$CHBr_3$	-1285.850502	-1285.850456	-1285.850421	-1285.849872
CH_3I	-334.700441	-334.700402	-334.700372	-334.699902
CH_2I_2	-628.983089	-628.983037	-628.982996	-628.982363
CHI_3	-923.263156	-923.263106	-923.263067	-923.262455
CH_2FCl	-598.669149	-598.669089	-598.669042	-598.668317
CH_2FBr	-554.696137	-554.696078	-554.696033	-554.695320
CH_2FI	-433.830227	-433.830169	-433.830124	-433.829421
CH_2ClBr	-914.679234	-914.679179	-914.679136	-914.678471
CH_2ClI	-793.815755	-793.815701	-793.815659	-793.815002
CH_2BrI	-749.845422	-749.845368	-749.845327	-749.844685
CHF_2Cl	-697.815441	-697.815393	-697.815356	-697.814773
CHF_2Br	-653.840956	-653.840908	-653.840870	-653.840280
CHF_2I	-532.973097	-532.973046	-532.973007	-532.972395
$CHCl_2F$	-1057.785963	-1057.785917	-1057.785881	-1057.785323
$CHCl_2Br$	-1373.791807	-1373.791762	-1373.791728	-1373.791188
$CHCl_2I$	-1252.928184	-1252.928137	-1252.928101	-1252.927536
$CHBr_2F$	-969.840566	-969.840518	-969.840482	-969.839906
$CHBr_2Cl$	-1329.821010	-1329.820964	-1329.820929	-1329.820377
$CHBr_2I$	-1164.987563	-1164.987515	-1164.987478	-1164.986898
CHI_2F	-728.110208	-728.110157	-728.110118	-728.109501
CHI_2Cl	-1088.094926	-1088.094877	-1088.094839	-1088.094243
CHI_2Br	-1044.125088	-1044.125039	-1044.125001	-1044.124402
CHBrClF	-1013.813073	-1013.813027	-1013.812991	-1013.812424
CHIClF	-892.947318	-892.947270	-892.947232	-892.946644
CHIBrF	-848.975107	-848.975058	-848.975020	-848.974426
CHIBrCl	-1208.957667	-1208.957620	-1208.957584	-1208.957014

Table S44. B3LYP/aT/SMD Total Energies for Anionic Species in a.u.

Species	Water	DMSO	MeCN	THF
CH_3^-	-39.970722	-39.970754	-39.971366	-39.957477
CH_2F^-	-139.246146	-139.246504	-139.247203	-139.233974
CHF_2^-	-238.543667	-238.544320	-238.545118	-238.532802
CF_3^-	-337.860751	-337.861682	-337.862577	-337.851376
CH_2Cl^-	-499.616715	-499.617980	-499.618789	-499.607163
$CHCl_2^-$	-959.265133	-959.267502	-959.268447	-959.258502
CCl_3^-	-1418.908589	-1418.911935	-1418.912969	-1418.904370
CH_2Br^-	-456.301127	-456.302448	-456.303300	-456.292655
$CHBr_2^-$	-872.635726	-872.638204	-872.639207	-872.630824
CBr_3^-	-1288.966663	-1288.970145	-1288.971218	-1288.964149
CH_2I^-	-335.193007	-335.190882	-335.192017	-335.181243
CHI_2^-	-630.410414	-630.406223	-630.407760	-630.399009
CI_3^-	-925.620449	-925.614381	-925.616237	-925.608726
$CHFCl^-$	-598.903190	-598.904751	-598.905669	-598.894882
$CHFBr^-$	-555.586931	-555.588572	-555.589548	-555.579823
$CHFI^-$	-434.476049	-434.474220	-434.475489	-434.465560
$CHClBr^-$	-915.950237	-915.952665	-915.953643	-915.944537
$CHClI^-$	-794.838271	-794.837326	-794.838578	-794.829263
$CHBrI^-$	-751.524544	-751.523657	-751.524918	-751.516167
CF_2Cl^-	-698.211109	-698.213001	-698.214051	-698.204279
CF_2Br^-	-654.895166	-654.897178	-654.898307	-654.889782
CF_2I^-	-533.788365	-533.786705	-533.788109	-533.778569
CCl_2F^-	-1058.557919	-1058.560585	-1058.561651	-1058.552653
CCl_2Br^-	-1375.594312	-1375.597723	-1375.598786	-1375.590689
CCl_2I^-	-1254.480062	-1254.480199	-1254.481535	-1254.473317
CBr_2F^-	-971.928881	-971.931684	-971.932810	-971.925302
CBr_2Cl^-	-1332.280251	-1332.283711	-1332.284789	-1332.277270
CBr_2I^-	-1167.853177	-1167.853426	-1167.854755	-1167.847290
CI_2F^-	-729.699162	-729.695256	-729.696941	-729.689166
CI_2Cl^-	-1090.050514	-1090.047509	-1090.049115	-1090.041293
CI_2Br^-	-1046.737746	-1046.734815	-1046.736408	-1046.728873
$CBrClF^-$	-1015.243126	-1015.245868	-1015.246969	-1015.238786
$CIClF^-$	-894.130212	-894.129538	-894.130925	-894.122475
$CIBrF^-$	-850.816123	-850.815527	-850.816918	-850.808993
$CIBrCl^-$	-1211.166557	-1211.166755	-1211.168091	-1211.160293

Table S45. B3LYP/aT/COSMO Total Energies for Anionic Species in a.u.

Species	Water	DMSO	MeCN	THF
CH_3^-	-39.973733	-39.972699	-39.971898	-39.959164
CH_2F^-	-139.250612	-139.249614	-139.248840	-139.236517
CHF_2^-	-238.549552	-238.548605	-238.547871	-238.536164
CF_3^-	-337.867797	-337.866916	-337.866233	-337.855316
CH_2Cl^-	-499.623220	-499.622313	-499.621610	-499.610412
$CHCl_2^-$	-959.272728	-959.271924	-959.271301	-959.261358
CCl_3^-	-1418.915951	-1418.915224	-1418.914660	-1418.905656
CH_2Br^-	-456.312280	-456.311408	-456.310732	-456.299966
$CHBr_2^-$	-872.648382	-872.647626	-872.647040	-872.637694
CBr_3^-	-1288.977208	-1288.976529	-1288.976003	-1288.967606
CH_2I^-	-335.198726	-335.197900	-335.197261	-335.187078
CHI_2^-	-630.417930	-630.417234	-630.416695	-630.408108
CI_3^-	-925.628706	-925.628087	-925.627608	-925.619968
$CHFCl^-$	-598.911028	-598.910169	-598.909503	-598.898873
$CHFBr^-$	-555.599231	-555.598407	-555.597768	-555.587578
$CHFI^-$	-434.483407	-434.482631	-434.482029	-434.472438
$CHClBr^-$	-915.960607	-915.959828	-915.959225	-915.949599
$CHClI^-$	-794.845092	-794.844350	-794.843774	-794.834601
$CHBrI^-$	-751.533003	-751.532278	-751.531717	-751.522774
CF_2Cl^-	-698.221634	-698.220815	-698.220180	-698.210041
CF_2Br^-	-654.911837	-654.911041	-654.910424	-654.900581
CF_2I^-	-533.797337	-533.796580	-533.795993	-533.786637
CCl_2F^-	-1058.566725	-1058.565971	-1058.565386	-1058.556048
CCl_2Br^-	-1375.603177	-1375.602468	-1375.601919	-1375.593153
CCl_2I^-	-1254.486738	-1254.486060	-1254.485534	-1254.477147
CBr_2F^-	-971.941962	-971.941253	-971.940704	-971.931925
CBr_2Cl^-	-1332.290262	-1332.289569	-1332.289032	-1332.280467
CBr_2I^-	-1167.860810	-1167.860154	-1167.859646	-1167.851544
CI_2F^-	-729.708458	-729.707809	-729.707306	-729.699281
CI_2Cl^-	-1090.057606	-1090.056963	-1090.056464	-1090.048514
CI_2Br^-	-1046.744628	-1046.743992	-1046.743500	-1046.735648
$CBrClF^-$	-1015.254610	-1015.253879	-1015.253313	-1015.244268
$CIClF^-$	-894.138332	-894.137637	-894.137098	-894.128500
$CIBrF^-$	-850.825305	-850.824628	-850.824103	-850.815731
$CIBrCl^-$	-1211.173839	-1211.173173	-1211.172657	-1211.164425

Table S46. MP2/aT/SMD Total Energies for Anionic Species in a.u.

Species	Water	DMSO	MeCN	THF
CH_3^-	-39.849567	-39.849591	-39.850197	-39.836209
CH_2F^-	-138.982711	-138.983055	-138.983741	-138.970386
CHF_2^-	-238.141330	-238.141967	-238.142754	-238.130310
CF_3^-	-337.322470	-337.323385	-337.324271	-337.312968
CH_2Cl^-	-498.980201	-498.981430	-498.982191	-498.970293
$CHCl_2^-$	-958.115846	-958.118148	-958.119037	-958.108765
CCl_3^-	-1417.249707	-1417.252973	-1417.253963	-1417.244929
CH_2Br^-	-455.006970	-455.008244	-455.009031	-454.998022
$CHBr_2^-$	-870.171529	-870.173918	-870.174848	-870.166060
CBr_3^-	-1285.336298	-1285.339674	-1285.340701	-1285.333323
CH_2I^-	-334.152690	-334.150571	-334.151639	-334.140600
CHI_2^-	-628.455144	-628.451009	-628.452464	-628.443429
CI_3^-	-922.755377	-922.749408	-922.751190	-922.743374
$CHFCl^-$	-598.125623	-598.127133	-598.128002	-598.116921
$CHFBr^-$	-554.151076	-554.152635	-554.153531	-554.143285
$CHFI^-$	-433.291572	-433.289773	-433.290961	-433.280769
$CHClBr^-$	-914.143418	-914.145771	-914.146685	-914.137219
$CHClI^-$	-793.285077	-793.284128	-793.285305	-793.275667
$CHBrI^-$	-749.314450	-749.313550	-749.314735	-749.305667
CF_2Cl^-	-697.292775	-697.294589	-697.295583	-697.285644
CF_2Br^-	-653.317091	-653.319005	-653.320061	-653.311253
CF_2I^-	-532.459904	-532.458277	-532.459633	-532.450073
CCl_2F^-	-1057.267915	-1057.270478	-1057.271479	-1057.262091
CCl_2Br^-	-1373.278049	-1373.281346	-1373.282346	-1373.273844
CCl_2I^-	-1252.417176	-1252.417290	-1252.418553	-1252.409947
CBr_2F^-	-969.321912	-969.324574	-969.325614	-969.317560
CBr_2Cl^-	-1329.306784	-1329.310123	-1329.311138	-1329.303224
CBr_2I^-	-1164.477235	-1164.477449	-1164.478720	-1164.471004
CI_2F^-	-727.597611	-727.588987	-727.590668	-727.582859
CI_2Cl^-	-1087.585734	-1087.582771	-1087.584298	-1087.576099
CI_2Br^-	-1043.617040	-1043.614141	-1043.615665	-1043.607820
$CBrClF^-$	-1013.294616	-1013.297231	-1013.298253	-1013.289555
$CIClF^-$	-892.432413	-892.431759	-892.433066	-892.424397
$CIBrF^-$	-848.460866	-848.460269	-848.461581	-848.453382
$CIBrCl^-$	-1208.446965	-1208.447130	-1208.448398	-1208.440255

Table S47. MP2/aT/COSMO Total Energies for Anion Species in a.u.

Species	Water	DMSO	MeCN	THF
CH_3^-	-39.852491	-39.851451	-39.850645	-39.837833
CH_2F^-	-138.986999	-138.985992	-138.985211	-138.972782
CHF_2^-	-238.147076	-238.146122	-238.145383	-238.133582
CF_3^-	-337.329527	-337.328639	-337.327950	-337.316938
CH_2Cl^-	-498.986133	-498.985215	-498.984503	-498.973163
$CHCl_2^-$	-958.122865	-958.122047	-958.121413	-958.111304
CCl_3^-	-1417.256668	-1417.255924	-1417.255347	-1417.246139
CH_2Br^-	-455.017419	-455.016533	-455.015846	-455.004907
$CHBr_2^-$	-870.183857	-870.183083	-870.182483	-870.172924
CBr_3^-	-1285.347105	-1285.346406	-1285.345864	-1285.337232
CH_2I^-	-334.157997	-334.157156	-334.156504	-334.146137
CHI_2^-	-628.462278	-628.461564	-628.461011	-628.452208
CI_3^-	-922.763517	-922.762881	-922.762389	-922.754554
$CHFCl^-$	-598.132646	-598.131774	-598.131098	-598.120312
$CHFBr^-$	-554.161903	-554.161063	-554.160412	-554.150026
$CHFI^-$	-433.298317	-433.297522	-433.296907	-433.287092
$CHClBr^-$	-914.153201	-914.152406	-914.151789	-914.141967
$CHClI^-$	-793.291482	-793.290720	-793.290130	-793.280730
$CHBrI^-$	-749.322630	-749.321887	-749.321312	-749.312149
CF_2Cl^-	-697.301687	-697.300874	-697.300245	-697.290179
CF_2Br^-	-653.331022	-653.330239	-653.329631	-653.319927
CF_2I^-	-532.468720	-532.467958	-532.467368	-532.457952
CCl_2F^-	-1057.275768	-1057.274999	-1057.274403	-1057.264881
CCl_2Br^-	-1373.286477	-1373.285750	-1373.285186	-1373.276191
CCl_2I^-	-1252.423527	-1252.422827	-1252.422283	-1252.413628
CBr_2F^-	-969.333816	-969.333091	-969.332529	-969.323552
CBr_2Cl^-	-1329.316645	-1329.315932	-1329.315380	-1329.306574
CBr_2I^-	-1164.485114	-1164.484438	-1164.483914	-1164.475573
CI_2F^-	-727.606212	-727.601651	-727.601146	-727.593098
CI_2Cl^-	-1087.592499	-1087.591834	-1087.591319	-1087.583114
CI_2Br^-	-1043.623924	-1043.623269	-1043.622762	-1043.614686
$CBrClF^-$	-1013.304657	-1013.303911	-1013.303333	-1013.294102
$CIClF^-$	-892.440040	-892.439330	-892.438780	-892.430005
$CIBrF^-$	-848.469634	-848.468941	-848.468404	-848.459835
$CIBrCl^-$	-1208.454164	-1208.453476	-1208.452944	-1208.444454

Table S48. CCSD(T) Total Energies for Neutral Species in a.u. "FC" Denotes that valence-only correlation was implemented in the calculation to distinguish it from the small core awCT(-PP) calculation.

Species	aT(-PP)	awCT(-PP) FC	awCT(-PP)	aT-DK	aQ(-PP)	a5(-PP)
CH_4	-40.440895	-40.442673	-40.491304	-40.455576	-40.451718	-40.454897
CH_3F	-139.571272	-139.579607	-139.683232	-139.672714	-139.608372	-139.620502
CH_2F_2	-238.721032	-238.736001	-238.894665	-238.909272	-238.784645	-238.805752
CHF_3	-337.881191	-337.902856	-338.116621	-338.156243	-337.971493	-338.001603
CH_3Cl	-499.575396	-499.582910	-499.905105	-501.002784	-499.606452	-499.616589
CH_2Cl_2	-958.709801	-958.723170	-959.319007	-961.550036	-958.761084	-958.778273
$CHCl_3$	-1417.840903	-1417.860280	-1418.729808	-1422.094088	-1417.912499	-1417.936854
CH_3Br	-455.534559	-455.540676	-456.338122	-2644.566395	-455.561819	-455.570419
CH_2Br_2	-870.626229	-870.636756	-872.183227	-5248.675175	-870.669953	-870.684036
$CHBr_3$	-1285.714108	-1285.729143	-1288.024760	-7852.780151	-1285.774509	-1285.794166
CH_3I	-334.664693	-334.672701	-335.471249	-7153.059305	-334.691887	-334.699831
CH_2I_2	-628.885110	-628.899566	-630.448271	-14265.659659	-628.928877	-628.941696
CHI_3	-923.101836	-923.122940	-925.421933	-21378.256298	-923.162683	-923.180555
CH_2FCl	-598.712406	-598.726519	-599.103757	-600.226643	-598.769739	-598.788843
CH_2FBr	-554.669015	-554.681725	-555.534318	-2743.787634	-554.722507	-554.740043
CH_2FI	-433.795745	-433.810322	-434.664071	-7252.277148	-433.849102	-433.865968
CH_2ClBr	-914.667890	-914.679872	-915.751035	-3105.112469	-914.715389	-914.731039
CH_2ClI	-793.796592	-793.810529	-794.882854	-7613.603978	-793.844061	-793.859083
CH_2BrI	-749.755380	-749.767851	-751.315448	-9757.167123	-749.799096	-749.812541
CHF_2Cl	-697.862291	-697.883090	-698.315414	-699.463384	-697.946152	-697.974250
CHF_2Br	-653.817174	-653.836567	-654.744313	-2843.022594	-653.897139	-653.923650
CHF_2I	-532.941958	-532.963228	-533.872133	-7351.510183	-533.021746	-533.047578
$CHCl_2F$	-1057.848800	-1057.868845	-1058.519759	-1060.775939	-1057.926421	-1057.952601
$CHCl_2Br$	-1373.798431	-1373.816417	-1375.161322	-3565.655872	-1373.866284	-1373.889095
$CHCl_2I$	-1252.926591	-1252.946586	-1254.292670	-8074.146864	-1252.994483	-1253.016703
$CHBr_2F$	-969.761396	-969.778577	-971.380282	-5347.897151	-969.831332	-969.854355
$CHBr_2Cl$	-1329.756161	-1329.772704	-1331.592972	-5709.217886	-1329.820285	-1329.841531

$CHBr_2I$	-1164.842940	-1164.859973	-1167.156726	-12361.271785	-1164.903443	-1164.922496
CHI_2F	-728.015327	-728.036398	-729.640409	-14364.876692	-728.085230	-728.106971
CHI_2Cl	-1088.013521	-1088.034091	-1089.856676	-14726.200890	-1088.077821	-1088.097879
CHI_2Br	-1043.972166	-1043.991219	-1046.289100	-16869.763816	-1044.032817	-1044.051276
CHBrClF	-1013.805010	-1013.823654	-1014.949969	-3204.336449	-1013.878784	-1013.903394
CHIClF	-892.931474	-892.952059	-894.079552	-7712.825722	-893.005170	-893.029141
CHIBrF	-848.888162	-848.907278	-850.510147	-9856.386716	-848.958056	-848.980435
CHIBrCl	-1208.884648	-1208.903190	-1210.724621	-10217.709191	-1208.948836	-1208.969482

Table S49. CCSD(T) Total Energies for Radical Species in a.u. "FC" Denotes that valence-only correlation was implemented in the calculation to distinguish it from the small core awCT(-PP) calculation.

Species	aT(-PP)	awCT(-PP) FC	awCT(-PP)	aT-DK	aQ(-PP)	a5(-PP)
$CH_3^•$	-39.763605	-39.765349	-39.813707	-39.778313	-39.773305	-39.776114
$CH_2F^•$	-138.899447	-138.907861	-139.011357	-139.000921	-138.935660	-138.947497
$CHF_2^•$	-238.048777	-238.063925	-238.222475	-238.237063	-238.111611	-238.132501
$CF_3^•$	-337.201226	-337.223167	-337.436778	-337.476337	-337.290788	-337.320758
$CH_2Cl^•$	-498.905443	-498.913552	-499.235656	-500.332830	-498.936015	-498.946231
$CHCl_2^•$	-958.045053	-958.059403	-958.655199	-960.885258	-958.096368	-958.113920
$CCl_3^•$	-1417.180431	-1417.201006	-1418.070473	-1421.433559	-1417.252520	-1417.277387
$CH_2Br^•$	-454.863098	-454.869348	-455.666536	-2643.894895	-454.889617	-454.898012
$CHBr_2^•$	-869.960493	-869.971236	-871.517351	-5248.009380	-870.003811	-870.017779
$CBr_3^•$	-1285.054749	-1285.070052	-1287.365170	-7852.120718	-1285.115029	-1285.134629
$CH_2I^•$	-333.992957	-334.001387	-334.799557	-7152.387626	-334.019399	-334.027125
$CHI_2^•$	-628.220681	-628.235838	-629.784007	-14264.995305	-628.264041	-628.276742
$CI_3^•$	-922.446076	-922.468079	-924.766418	-21377.600670	-922.506823	-922.524684
$CHFCl^•$	-598.043655	-598.058295	-598.435441	-599.557912	-598.100567	-598.119713
$CHFBr^•$	-553.999920	-554.012824	-554.865157	-2743.118519	-554.052797	-554.070169
$CHFI^•$	-433.127468	-433.142458	-433.995798	-7251.608941	-433.180222	-433.196922
$CHClBr^•$	-914.002646	-914.015223	-915.086186	-3104.447187	-914.049952	-914.065720
$CHClI^•$	-793.132044	-793.146815	-794.218820	-7612.939455	-793.179309	-793.194449
$CHBrI^•$	-749.090308	-749.103234	-750.650382	-9756.502058	-749.133617	-749.146945
$CF_2Cl^•$	-697.189751	-697.211018	-697.643186	-698.790901	-697.273162	-697.301272
$CF_2Br^•$	-653.146521	-653.166185	-654.073583	-2842.351950	-653.225920	-653.252304
$CF_2I^•$	-532.274730	-532.296383	-533.204726	-7350.843040	-532.353954	-532.379665
$CCl_2F^•$	-1057.182435	-1057.203243	-1057.854030	-1060.109588	-1057.260006	-1057.286421
$CCl_2Br^•$	-1373.138432	-1373.157291	-1374.501972	-3564.995833	-1373.206552	-1373.229670
$CCl_2I^•$	-1252.268268	-1252.289344	-1253.635097	-8073.488540	-1252.336406	-1252.358945
$CBr_2F^•$	-969.096872	-969.114315	-970.715565	-5347.232583	-969.166473	-969.189411
$CBr_2Cl^•$	-1329.096535	-1329.113646	-1330.933547	-5708.558211	-1329.160725	-1329.182094

$CBr_2I^•$	-1164.184974	-1164.202462	-1166.498642	-12360.613812	-1164.245339	-1164.264345
$CI_2F^•$	-727.354585	-727.376204	-728.979495	-14364.216045	-727.424145	-727.445814
$CI_2Cl^•$	-1087.356745	-1087.378292	-1089.200357	-14725.544175	-1087.421084	-1087.441295
$CI_2Br^•$	-1043.315389	-1043.335108	-1045.632369	-16869.107103	-1043.375908	-1043.394335
$CBrClF^•$	-1013.139623	-1013.158775	-1014.284790	-3203.671054	-1013.213197	-1013.237882
$CIClF^•$	-892.268397	-892.289640	-893.416660	-7712.162700	-892.341890	-892.365942
$CIBrF^•$	-848.225720	-848.245239	-849.847499	-9855.724297	-848.295274	-848.317572
$CIBrCl^•$	-1208.226552	-1208.245867	-1210.066840	-10217.051099	-1208.290789	-1208.311573

Table S50. CCSD(T) Total Energies for Anionic Species in a.u. "FC" Denotes that valence-only correlation was implemented in the calculation to distinguish it from the small core awCT(-PP) calculation.

Species	aT(-PP)	awCT(-PP) FC	awCT(-PP)	aT-DK	aQ(-PP)	a5(-PP)
CH_3^-	-39.763259	-39.764984	-39.813174	-39.777945	-39.773904	-39.777161
CH_2F^-	-138.904426	-138.912787	-139.015880	-139.005890	-138.941229	-138.953315
CHF_2^-	-238.072124	-238.087170	-238.245190	-238.260423	-238.135253	-238.156345
CF_3^-	-337.263189	-337.284987	-337.497986	-337.538375	-337.352787	-337.382969
CH_2Cl^-	-498.929292	-498.936640	-499.258158	-500.356644	-498.960523	-498.970672
$CHCl_2^-$	-958.095932	-958.108960	-958.703884	-960.936148	-958.147850	-958.165159
CCl_3^-	-1417.255371	-1417.274163	-1418.142525	-1421.508632	-1417.327903	-1417.352428
CH_2Br^-	-454.895080	-454.901058	-455.697586	-2643.926973	-454.922853	-454.931548
$CHBr_2^-$	-870.022452	-870.032774	-871.577979	-5248.071488	-870.067476	-870.081863
CBr_3^-	-1285.140005	-1285.154747	-1287.448784	-7852.206083	-1285.202220	-1285.222340
CH_2I^-	-334.033193	-334.040968	-334.838465	-7152.427948	-334.061236	-334.069402
CHI_2^-	-628.291555	-628.305511	-629.852823	-14265.066320	-628.337111	-628.350425
CI_3^-	-922.537845	-922.558053	-924.855287	-21377.692604	-922.601006	-922.619542
$CHFCl^-$	-598.084213	-598.098035	-598.474442	-599.598485	-598.141573	-598.160627
$CHFBr^-$	-554.048154	-554.060756	-554.912301	-2743.166855	-554.102170	-554.119844
$CHFI^-$	-433.182773	-433.197021	-434.049588	-7251.664370	-433.237051	-433.254192
$CHClBr^-$	-914.059259	-914.070962	-915.141030	-3104.503893	-914.107736	-914.123599
$CHClI^-$	-793.193725	-793.207225	-794.278365	-7613.001238	-793.242448	-793.257790
$CHBrI^-$	-749.156931	-749.169043	-750.715303	-9756.568834	-749.202207	-749.216056
CF_2Cl^-	-697.262362	-697.282663	-697.713941	-698.863598	-697.345961	-697.373919
CF_2Br^-	-653.226723	-653.246020	-654.152439	-2842.432325	-653.307331	-653.334073
CF_2I^-	-532.362168	-532.382962	-533.290087	-7350.930740	-532.443727	-532.470095
CCl_2F^-	-1057.257299	-1057.276638	-1057.926409	-1060.184572	-1057.335172	-1057.361275
CCl_2Br^-	-1373.217004	-1373.234507	-1374.578102	-3565.074548	-1373.286110	-1373.309200
CCl_2I^-	-1252.349366	-1252.368651	-1253.713332	-8073.569817	-1252.418719	-1252.441299
CBr_2F^-	-969.181043	-969.197988	-970.798232	-5347.316890	-969.252274	-969.275609
CBr_2Cl^-	-1329.178544	-1329.194696	-1331.013517	-5708.640361	-1329.244208	-1329.265829

CBr_2I^-	-1164.272416	-1164.288933	-1166.584041	-12360.701380	-1164.334913	-1164.354499
CI_2F^-	-727.445327	-727.465233	-729.066827	-14364.307018	-727.516986	-727.539167
CI_2Cl^-	-1087.443422	-1087.463181	-1089.284162	-14725.631038	-1087.509642	-1087.530227
CI_2Br^-	-1043.405014	-1043.423352	-1045.719527	-16869.196871	-1043.467825	-1043.486883
$CBrClF^-$	-1013.219353	-1013.237520	-1014.362521	-3203.750923	-1013.293915	-1013.318650
$CIClF^-$	-892.351659	-892.371469	-893.497513	-7712.246153	-892.426537	-892.450768
$CIBrF^-$	-848.313085	-848.331655	-849.932943	-9855.811841	-848.384597	-848.407400
$CIBrCl^-$	-1208.310924	-1208.328856	-1210.148753	-10217.135638	-1208.376850	-1208.397949

Table S51. CCSD(T) CBS Extrapolation Total Energies for Neutral Species in a.u.

Species	TQ	Q5	TQ5
CH_4	-40.457964	-40.457479	-40.456739
CH_3F	-139.629784	-139.630351	-139.627544
CH_2F_2	-238.821360	-238.822891	-238.818010
CHF_3	-338.023611	-338.026052	-338.019092
CH_3Cl	-499.624376	-499.624820	-499.622474
CH_2Cl_2	-958.790682	-958.792231	-958.788258
$CHCl_3$	-1417.953822	-1417.956631	-1417.951007
CH_3Br	-455.577552	-455.577402	-455.575409
CH_2Br_2	-870.695189	-870.695471	-870.692210
$CHBr_3$	-1285.809371	-1285.810128	-1285.805578
CH_3I	-334.707583	-334.706282	-334.704432
CH_2I_2	-628.954138	-628.952106	-628.949121
CHI_3	-923.197801	-923.195067	-923.190907
CH_2FCl	-598.802830	-598.804356	-598.799939
CH_2FBr	-554.753381	-554.754282	-554.750225
CH_2FI	-433.879898	-433.879663	-433.875753
CH_2ClBr	-914.742804	-914.743747	-914.740127
CH_2ClI	-793.871458	-793.871281	-793.867799
CH_2BrI	-749.824327	-749.823458	-749.820337
CHF_2Cl	-697.994554	-697.997066	-697.990572
CHF_2Br	-653.943292	-653.945177	-653.939047
CHF_2I	-533.067797	-533.068554	-533.062573
$CHCl_2F$	-1057.971221	-1057.973859	-1057.967811
$CHCl_2Br$	-1373.905446	-1373.907618	-1373.902347
$CHCl_2I$	-1253.033668	-1253.034746	-1253.029604
$CHBr_2F$	-969.871697	-969.873050	-969.867724
$CHBr_2Cl$	-1329.857295	-1329.858783	-1329.853870
$CHBr_2I$	-1164.938363	-1164.937967	-1164.933549
CHI_2F	-728.125575	-728.124625	-728.119580
CHI_2Cl	-1088.114933	-1088.114166	-1088.109513
CHI_2Br	-1044.067823	-1044.066265	-1044.061977
CHBrClF	-1013.921364	-1013.923377	-1013.917689
CHIClF	-893.047705	-893.048606	-893.043057
CHIBrF	-848.998396	-848.998607	-848.993423
CHIBrCl	-1208.985883	-1208.986247	-1208.981465

Table S52. CCSD(T) CBS Extrapolation Total Energies for Radical Species in a.u.

Species	TQ	Q5	TQ5
$CH_3^{\bullet}$	-39.778904	-39.778394	-39.777740
$CH_2F^{\bullet}$	-138.956561	-138.957108	-138.954369
$CHF_2^{\bullet}$	-238.147876	-238.149465	-238.144635
$CF_3^{\bullet}$	-337.342480	-337.345094	-337.338168
$CH_2Cl^{\bullet}$	-498.953660	-498.954527	-498.952165
$CHCl_2^{\bullet}$	-958.125985	-958.128172	-958.124120
$CCl_3^{\bullet}$	-1417.294127	-1417.297579	-1417.291841
$CH_2Br^{\bullet}$	-454.904923	-454.904829	-454.902883
$CHBr_2^{\bullet}$	-870.028812	-870.029121	-870.025887
$CBr_3^{\bullet}$	-1285.149820	-1285.150545	-1285.146008
$CH_2I^{\bullet}$	-334.034660	-334.033400	-334.031600
$CHI_2^{\bullet}$	-628.289067	-628.287055	-628.284098
$CI_3^{\bullet}$	-922.541885	-922.539187	-922.535029
$CHFCl^{\bullet}$	-598.133415	-598.135260	-598.130836
$CHFBr^{\bullet}$	-554.083316	-554.084275	-554.080256
$CHFI^{\bullet}$	-433.210670	-433.210482	-433.206611
$CHClBr^{\bullet}$	-914.077255	-914.078524	-914.074879
$CHClI^{\bullet}$	-793.206589	-793.206743	-793.203236
$CHBrI^{\bullet}$	-749.158613	-749.157767	-749.154673
$CF_2Cl^{\bullet}$	-697.321304	-697.324097	-697.317603
$CF_2Br^{\bullet}$	-653.271746	-653.273728	-653.267628
$CF_2I^{\bullet}$	-532.399679	-532.400542	-532.394591
$CCl_2F^{\bullet}$	-1057.304777	-1057.307870	-1057.301771
$CCl_2Br^{\bullet}$	-1373.245868	-1373.248442	-1373.243103
$CCl_2I^{\bullet}$	-1252.375733	-1252.377247	-1252.372035
$CBr_2F^{\bullet}$	-969.206644	-969.208037	-969.202731
$CBr_2Cl^{\bullet}$	-1329.197773	-1329.199446	-1329.194506
$CBr_2I^{\bullet}$	-1164.280179	-1164.279778	-1164.275371
$CI_2F^{\bullet}$	-727.464292	-727.463409	-727.458382
$CI_2Cl^{\bullet}$	-1087.458218	-1087.457706	-1087.453020
$CI_2Br^{\bullet}$	-1043.410837	-1043.409298	-1043.405017
$CBrClF^{\bullet}$	-1013.255661	-1013.257926	-1013.252222
$CIClF^{\bullet}$	-892.384307	-892.385472	-892.379907
$CIBrF^{\bullet}$	-848.335418	-848.335678	-848.330513
$CIBrCl^{\bullet}$	-1208.327864	-1208.328450	-1208.323638

Table S53. CCSD(T) CBS Extrapolation Total Energies for Anionic Species in a.u.

Species	TQ	Q5	TQ5
CH_3^-	-39.780047	-39.779806	-39.779049
CH_2F^-	-138.962471	-138.963128	-138.960332
CHF_2^-	-238.171689	-238.173472	-238.168597
CF_3^-	-337.404500	-337.407476	-337.400503
CH_2Cl^-	-498.978548	-498.978913	-498.976564
$CHCl_2^-$	-958.177815	-958.179214	-958.175213
CCl_3^-	-1417.369765	-1417.372343	-1417.366678
CH_2Br^-	-454.938883	-454.938608	-454.936592
$CHBr_2^-$	-870.093462	-870.093545	-870.090212
CBr_3^-	-1285.238128	-1285.238678	-1285.234020
CH_2I^-	-334.077421	-334.076032	-334.074131
CHI_2^-	-628.363404	-628.361236	-628.358136
CI_3^-	-922.637460	-922.634594	-922.630279
$CHFCl^-$	-598.174679	-598.176099	-598.171694
$CHFBr^-$	-554.133346	-554.134195	-554.130106
$CHFI^-$	-433.268378	-433.268111	-433.264137
$CHClBr^-$	-914.135715	-914.136480	-914.132809
$CHClI^-$	-793.270569	-793.270248	-793.266691
$CHBrI^-$	-749.228339	-749.227301	-749.224085
CF_2Cl^-	-697.394211	-697.396621	-697.390159
CF_2Br^-	-653.353855	-653.355788	-653.349604
CF_2I^-	-532.490800	-532.491506	-532.485401
CCl_2F^-	-1057.380117	-1057.382471	-1057.376439
CCl_2Br^-	-1373.325995	-1373.327949	-1373.322612
CCl_2I^-	-1252.458747	-1252.459634	-1252.454408
CBr_2F^-	-969.293386	-969.294557	-969.289158
CBr_2Cl^-	-1329.282107	-1329.283385	-1329.278384
CBr_2I^-	-1164.370984	-1164.370403	-1164.365860
CI_2F^-	-727.558345	-727.557178	-727.552030
CI_2Cl^-	-1087.547862	-1087.546942	-1087.542166
CI_2Br^-	-1043.504078	-1043.502358	-1043.497930
$CBrClF^-$	-1013.336950	-1013.338734	-1013.333015
$CIClF^-$	-892.469754	-892.470443	-892.464833
$CIBrF^-$	-848.425871	-848.425916	-848.420633
$CIBrCl^-$	-1208.414901	-1208.415082	-1208.410194

Additional experimental details

2-bromo-2,2-dichloro-N-phenethylacetamide (Reaction with LDA)

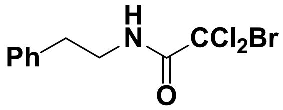

To a solution of phenethyl isocyanate (0.19 ml, 1.36 mmol, 1.0 equiv) in dry THF (5 mL), cooled at -78 °C, bromodichloromethane (0.34 ml, 4.08 mmol, 3.0 equiv) was added under argon atmosphere. After 10 min, LDA 2.0 M solution in THF (1.19 ml, 3.81 mmol, 2.8 equiv) was added with a syringe pump 0.20 mL/min and then the stirring was continued for 1 h. The mixture was quenched with (*aq.*) HCl 1.0 M (3 mL) and extracted with ethyl acetate (5 mL). The organic layer was washed with saturated (*aq.*) NaCl (5 mL), dried over anhydrous Na_2SO_4, filtered and concentrated under reduced pressure to give the compound in 72 % yield (305 mg) as a brown oil after column chromatography on neutral alumina Brockmann grade IV (petroleum ether/ethyl acetate 9:1 as eluent).

^{1}H NMR (400 MHz, $CDCl_3$) δ: 7.26 (m, 2H, Ph H-2,4), 7.16 (m, 3H, Ph H- 3,5,6), 6.77 (bs, 1H, NH), 3.55 (q, *J*= 6.6 Hz, 2H, CH_2), 2.84 (t, *J*= 7.0 Hz, 2H, CH_2).

^{13}C NMR (100 MHz, $CDCl_3$) δ: 162.0 (CO), 137.6 (Ph C-1), 128.6 (4C, Ph C-2, 3, 5, 6), 126.7 (Ph C-4), 74.9 (CCl_2Br), 42.4 (CH_2), 34.7 (CH_2).

HRMS (ESI), m/z: calcd. For $C_{10}H_{11}BrCl_2NO^+$: 309.9396 $[M+H]^+$; found:309,9392.
Spectroscopic and spectrometric data match with those ones reported for the other reactions.

2-bromo-2,2-dichloro-N-phenethylacetamide (Reaction with LTMP)

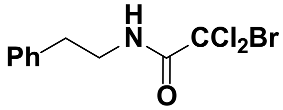

Preparation of LTMP. Freshly distilled 2,2,6,6-tetramethylpiperidine (TMP) (0.65 mL, 3.81 mmol, 2.8 equiv) was added to THF (3 mL) and the resulting mixture was cooled at 0 °C. Then, MeLi-LiBr (2.2 M solution in Et_2O) (1.73 mL, 3.81 mmol, 2.8 equiv) was added dropwise over 10 min. The so obtained solution was transferred via cannula to the solution indicated below.

Homologation.

To a solution of phenethyl isocyanate (0.19 ml, 1.36 mmol, 1.0 equiv) in dry THF (5 mL), cooled at -78 °C, bromodichloromethane (0.34 ml, 4.08 mmol, 3.0 equiv) was added under argon atmosphere. After 10 min, the above prepared solution of LTMP (2.8 equiv) was added during a period of 15 min and, then the stirring was continued for 1 h. The mixture was quenched with (*aq.*) HCl 1.0 M (3 mL) and extracted with ethyl acetate (5 mL). The organic layer was washed with saturated (*aq.*) NaCl (5 mL), dried over anhydrous Na_2SO_4, filtered and concentrated under reduced pressure to give the compound in 69 % yield (292 mg) as a brown oil after column

chromatography on neutral alumina Brockmann grade IV (petroleum ether/ethyl acetate 9:1 as eluent).

^{1}H NMR (400 MHz, $CDCl_3$) δ: 7.26 (m, 2H, Ph H-2,4), 7.16 (m, 3H, Ph H- 3,5,6), 6.77 (bs, 1H, NH), 3.55 (q, *J*= 6.6 Hz, 2H, CH_2), 2.84 (t, *J*= 7.0 Hz, 2H, CH_2).

^{13}C NMR (100 MHz, $CDCl_3$) δ: 162.0 (CO), 137.6 (Ph C-1), 128.6 (4C, Ph C-2, 3, 5, 6), 126.7 (Ph C-4), 74.9 (CCl_2Br), 42.4 (CH_2), 34.7 (CH_2).

HRMS (ESI), m/z: calcd. For $C_{10}H_{11}BrCl_2NO^+$: 309.9396 $[M+H]^+$; found:309,9395.
Spectroscopic and spectrometric data match with those ones reported for the other reactions.

2-bromo-2,2-dichloro-N-phenethylacetamide (Reaction with DBU)

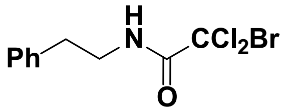

To the mixture of phenethyl isocyanate (0.19 ml, 1.36 mmol, 1.0 equiv) and bromodichloromethane (0.34 ml, 4.08 mmol, 3.0 equiv) under argon atmosphere, DBU (0.57 ml, 3.81 mmol, 2.8 equiv) was added dropwise during a period of 15 min at room temperature, and then the stirring was continued for 2 h. The mixture was quenched with (*aq.*) HCl 1.0 M (3 mL) and extracted with ethyl acetate (5 mL). The organic layer was washed with saturated (*aq.*) NaCl (5 mL), dried over anhydrous Na_2SO_4, filtered and concentrated under reduced pressure to give the compound in 50 % yield (212 mg) as a brown oil after column chromatography on neutral alumina Brockmann grade IV (petroleum ether/ethyl acetate 9:1 as eluent).

^{1}H NMR (400 MHz, $CDCl_3$) δ: 7.26 (m, 2H, Ph H-2,4), 7.16 (m, 3H, Ph H- 3,5,6), 6.77 (bs, 1H, NH), 3.55 (q, *J*= 6.6 Hz, 2H, CH_2), 2.84 (t, *J*= 7.0 Hz, 2H, CH_2).

^{13}C NMR (100 MHz, $CDCl_3$) δ: 162.0 (CO), 137.6 (Ph C-1), 128.6 (4C, Ph C-2, 3, 5, 6), 126.7 (Ph C-4), 74.9 (CCl_2Br), 42.4 (CH_2), 34.7 (CH_2).

HRMS (ESI), m/z: calcd. For $C_{10}H_{11}BrCl_2NO^+$: 309.9396 $[M+H]^+$; found:309,9394.

Spectroscopic and spectrometric data match with those ones reported for the other reactions.

2-bromo-2,2-dichloro-N-phenethylacetamide (Reaction with KOH)

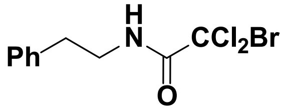

To a solution of phenethyl isocyanate (0.19 ml, 1.36 mmol, 1.0 equiv) in dry DMF (5 mL), bromodichloromethane (0.34 ml, 4.08 mmol, 3.0 equiv) was added under argon atmosphere. After cooling the mixture at 0°C, KOH (214 mg, 3.81 mmol, 2.8 equiv) was added during a period of 15 min and the stirring was continued for 3 h. The mixture was quenched with (*aq.*) HCl 1.0 M (3 mL) and extracted with ethyl acetate (5 mL). The organic layer was washed with saturated (*aq.*)

NaCl (5 mL), dried over anhydrous Na_2SO_4, filtered and concentrated under reduced pressure to give the compound in 36 % yield (152 mg) as a brown oil after column chromatography on neutral alumina Brockmann grade IV (petroleum ether/ethyl acetate 9:1 as eluent).

^{1}H NMR (400 MHz, $CDCl_3$) δ: 7.26 (m, 2H, Ph H-2,4), 7.16 (m, 3H, Ph H- 3,5,6), 6.77 (bs, 1H, NH), 3.55 (q, *J*= 6.6 Hz, 2H, CH_2), 2.84 (t, *J*= 7.0 Hz, 2H, CH_2).

^{13}C NMR (100 MHz, $CDCl_3$) δ: 162.0 (CO), 137.6 (Ph C-1), 128.6 (4C, Ph C-2, 3, 5, 6), 126.7 (Ph C-4), 74.9 (CCl_2Br), 42.4 (CH_2), 34.7 (CH_2).

HRMS (ESI), m/z: calcd. For $C_{10}H_{11}BrCl_2NO^+$: 309.9396 $[M+H]^+$; found:309,9392.
Spectroscopic and spectrometric data match with those ones reported for the other reactions.

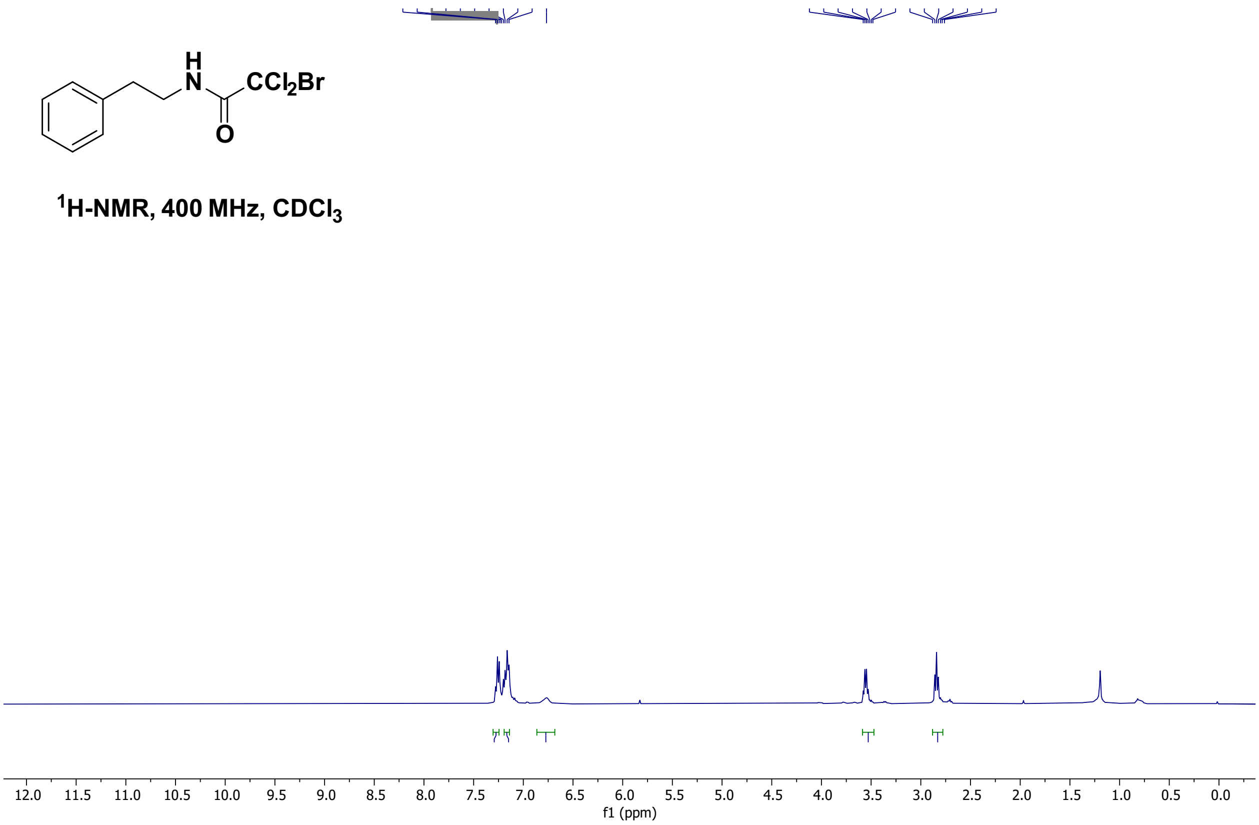

Figure S1 ^{1}H NMR Spectrum for 2-bromo-2,2-dichloro-N-phenethylacetamide

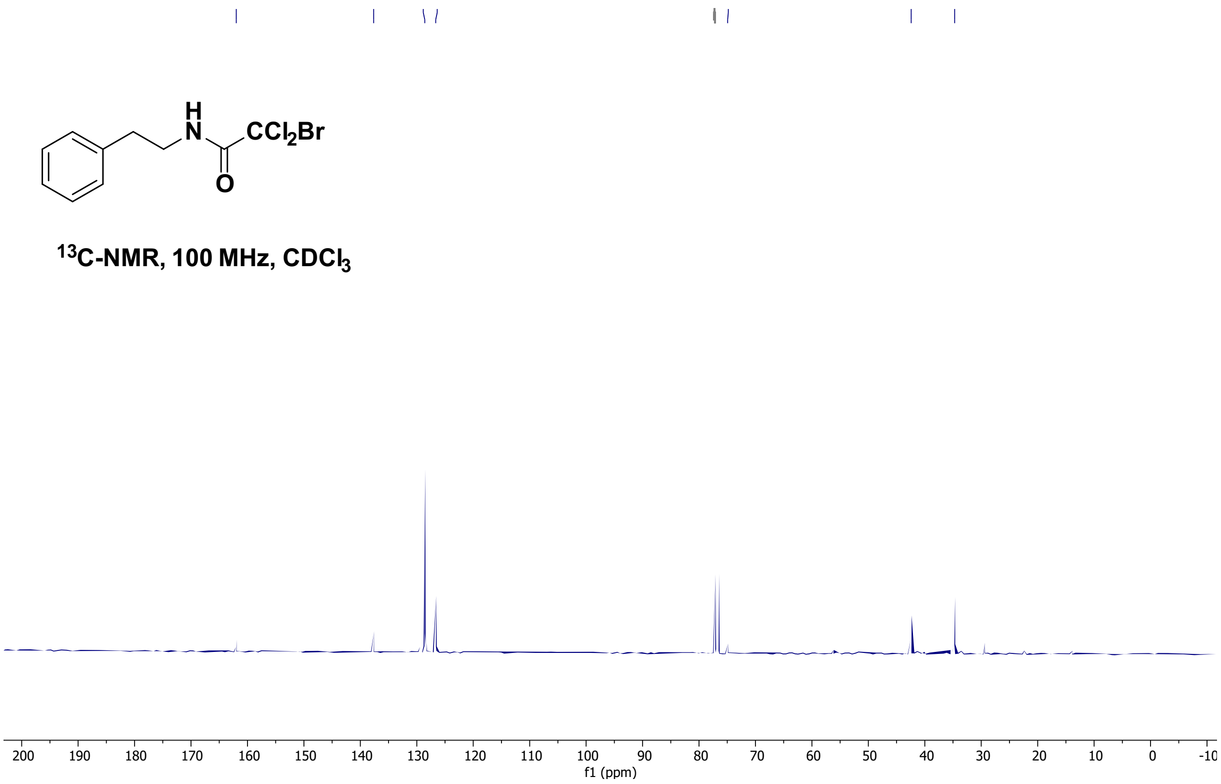

Figure S2. ^{13}C NMR Spectrum for 2-bromo-2,2-dichloro-N-phenethylacetamide

Table S54. Coordinates of Species

Optimized Coordinates from B3LYP/aug-cc-pVTZ Neutral Species

Benzoic Acid

C	-1.124024	-0.608274	-0.000000
C	-0.966837	-1.987395	0.000000
C	0.307570	-2.543911	0.000000
C	1.429585	-1.720551	0.000000
C	1.277727	-0.342621	0.000000
C	0.000000	0.220139	-0.000000
H	-2.111014	-0.171284	-0.000000
H	-1.837869	-2.628244	-0.000000
H	0.426718	-3.619052	0.000000
H	2.420155	-2.154175	0.000000
H	2.135288	0.314350	0.000000
C	-0.110802	1.700293	-0.000000
O	0.824222	2.465151	-0.000000
O	-1.394344	2.140480	-0.000000
H	-1.351615	3.107267	-0.000000

CH_4

C	0.000000	0.000000	0.000000
H	0.628146	0.628146	0.628146
H	-0.628146	-0.628146	0.628146
H	0.628146	-0.628146	-0.628146
H	-0.628146	0.628146	-0.628146

CH_3F

C	0.000000	0.000000	-0.637600
H	0.000000	1.032642	-0.986997
H	-0.894294	-0.516321	-0.986997
H	0.894294	-0.516321	-0.986997
F	0.000000	0.000000	0.754066

CH_2F_2

C	0.000000	0.505858	0.000000
H	-0.000044	1.102707	0.911883
H	-0.000044	1.102707	-0.911883
F	-1.105893	-0.291195	0.000000
F	1.105903	-0.291089	0.000000

CHF_3

C	0.000000	0.000000	0.340244
H	0.000000	0.000000	1.429034
F	0.000000	1.256297	-0.128537

F	-1.087985	-0.628148	-0.128537
F	1.087985	-0.628148	-0.128537

CH_3Cl

C	0.000000	0.000000	-1.138842
H	0.000000	1.030345	-1.477227
H	-0.892305	-0.515172	-1.477227
H	0.892305	-0.515172	-1.477227
CL	0.000000	0.000000	0.662631

CH_2Cl_2

C	0.000000	0.769467	0.000000
H	-0.000073	1.375080	0.896336
H	-0.000073	1.375080	-0.896336
CL	-1.489013	-0.216688	0.000000
CL	1.489022	-0.216663	0.000000

$CHCl_3$

C	-0.000005	-0.000004	0.457792
H	-0.000008	-0.000012	1.537624
CL	0.950260	-1.406149	-0.084004
CL	0.742654	1.526010	-0.084014
CL	-1.692911	-0.119859	-0.084004

CH_3Br

C	0.000000	0.000000	-1.537642
H	0.000000	1.032808	-1.864537
H	0.894438	-0.516404	-1.864537
H	-0.894438	-0.516404	-1.864537
BR	0.000000	0.000000	0.423413

CH_2Br_2

C	0.000000	0.000000	0.936825
H	-0.899115	0.000000	1.535767
H	0.899115	0.000000	1.535767
BR	0.000000	1.632224	-0.124178
BR	0.000000	-1.632224	-0.124178

$CHBr_3$

C	0.000000	0.000000	0.522074
H	0.000000	0.000000	1.600702
BR	0.000000	1.861877	-0.045078
BR	-1.612433	-0.930938	-0.045078
BR	1.612433	-0.930938	-0.045078

CH_3I

C	0.000000	0.000000	-1.834059
H	0.000000	1.033637	-2.156682
H	-0.895156	-0.516819	-2.156682
H	0.895156	-0.516819	-2.156682
I	0.000000	0.000000	0.329706

CH_2I_2

C	0.000000	0.000000	1.052523
H	-0.896218	0.000000	1.655595
H	0.896218	0.000000	1.655595
I	0.000000	1.827126	-0.090814
I	0.000000	-1.827126	-0.090814

CHI_3

C	0.000000	0.000000	0.552227
H	0.000000	0.000000	1.631261
I	0.000000	2.083813	-0.031098
I	-1.804635	-1.041907	-0.031098
I	1.804635	-1.041907	-0.031098

CH_2FCl

C	0.000000	0.811371	0.000000
H	0.337764	1.307054	0.904524
H	0.337764	1.307054	-0.904524
F	-1.364262	0.761626	0.000000
CL	0.682519	-0.843351	0.000000

CH_2FBr

C	-0.518258	-1.126376	0.000000
H	-1.088802	-1.296625	0.906590
H	-1.088802	-1.296625	-0.906590
BR	0.000000	0.762042	0.000000
F	0.587462	-1.924441	0.000000

CH_2FI

C	-0.526029	-1.490740	0.000000
H	-1.095343	-1.667935	0.905752
H	-1.095343	-1.667935	-0.905752
I	0.000000	0.617464	0.000000
F	0.594095	-2.271696	0.000000

CH_2ClBr

C	0.000000	1.030588	0.000000
H	0.324311	1.536520	0.898531
H	0.324311	1.536520	-0.898531
CL	-1.778075	0.961480	0.000000

BR	0.845104	-0.731478	0.000000

CH_2ClI

C	-0.796461	-1.089266	0.000000
H	-1.391199	-1.184265	0.897460
H	-1.391199	-1.184265	-0.897460
CL	0.444774	-2.363079	0.000000
I	0.000000	0.925971	0.000000

CH_2BrI

C	0.000000	1.089345	0.000000
H	-0.231399	1.645656	0.897001
H	-0.231399	1.645656	-0.897001
BR	1.911530	0.729682	0.000000
I	-1.253599	-0.667288	0.000000

CHF_2Cl

C	0.446888	-0.000011	0.366760
H	0.551803	-0.000010	1.448022
CL	-1.288422	-0.000011	-0.050637
F	1.037200	1.088367	-0.154872
F	1.037249	-1.088338	-0.154879

CHF_2Br

C	-0.426963	-0.921667	0.000000
H	-1.511042	-0.987145	0.000000
F	0.076846	-1.524241	1.088906
F	0.076846	-1.524241	-1.088906
BR	0.076846	0.970099	0.000000

CHF_2I

C	-0.453289	-1.301438	0.000000
H	-1.536055	-1.389704	0.000000
F	0.059941	-1.898014	1.090403
F	0.059941	-1.898014	-1.090403
I	0.059941	0.818162	0.000000

$CHCl_2F$

C	-0.178208	0.524305	0.000000
H	-1.074646	1.132380	0.000000
F	0.911440	1.319423	0.000000
CL	-0.178208	-0.475089	1.474517
CL	-0.178208	-0.475089	-1.474517

$CHCl_2Br$

C	0.676030	-0.142553	0.000000

H	1.577261	0.451494	0.000000
CL	0.676030	-1.142828	1.468584
CL	0.676030	-1.142828	-1.468584
BR	-0.817671	1.121713	0.000000

$CHCl_2I$

C	0.913403	-0.454778	0.000000
H	1.817368	0.135068	0.000000
CL	0.913403	-1.454793	1.468878
CL	0.913403	-1.454793	-1.468878
I	-0.723651	0.982199	0.000000

$CHBr_2F$

C	0.102233	0.791073	0.000000
H	1.009754	1.381384	0.000000
BR	0.102233	-0.293165	1.620976
BR	0.102233	-0.293165	-1.620976
F	-0.975499	1.599305	0.000000

$CHBr_2Cl$

C	-0.232402	0.601315	0.000000
H	-1.159512	1.153351	0.000000
BR	-0.232402	-0.495997	1.613791
BR	-0.232402	-0.495997	-1.613791
CL	1.107180	1.762267	0.000000

$CHBr_2I$

C	0.640551	-0.016685	0.000000
H	1.565039	0.539193	0.000000
BR	0.640551	-1.108163	1.611571
BR	0.640551	-1.108163	-1.611571
I	-0.948055	1.455327	0.000000

CHI_2F

C	0.071082	0.959677	0.000000
H	0.987007	1.537503	0.000000
I	0.071082	-0.221106	1.820530
I	0.071082	-0.221106	-1.820530
F	-0.994244	1.793513	0.000000

CHI_2Cl

C	-0.163325	0.816764	0.000000
H	-1.103772	1.346043	0.000000
I	-0.163325	-0.382766	1.808739
I	-0.163325	-0.382766	-1.808739
CL	1.140951	2.019209	0.000000

CHI_2Br

C	0.327277	0.534137	0.000000
H	1.263040	1.071092	0.000000
I	0.327277	-0.659553	1.805984
I	0.327277	-0.659553	-1.805984
BR	-1.083373	1.875335	0.000000

CHBrClF

C	0.578370	0.464130	0.412574
H	0.617397	0.595616	1.486711
CL	1.842079	-0.686845	-0.067493
BR	-1.213414	-0.186084	-0.028220
F	0.785170	1.645437	-0.203007

CHIClF

C	1.004249	0.477651	0.416834
H	1.071528	0.614331	1.488982
CL	2.180878	-0.758215	-0.070614
I	-1.051149	-0.101167	-0.016880
F	1.282104	1.641253	-0.210546

CHIBrF

C	-0.452524	0.747868	0.428790
H	-0.484206	0.915535	1.498003
I	1.459141	-0.181209	-0.017990
BR	-1.977525	-0.375154	-0.031475
F	-0.546865	1.925745	-0.223960

CHIBrCl

C	0.448131	0.481399	0.505601
H	0.471108	0.515034	1.583868
CL	0.732742	2.122423	-0.103834
BR	1.863446	-0.743639	-0.043528
I	-1.525229	-0.253910	-0.025072

Optimized Coordinates from MP2/aug-cc-pVTZ Neutral Species

Benzoic Acid

C	-1.130326	-0.600555	0.000000
C	-0.973269	-1.983744	0.000000
C	0.302723	-2.545717	0.000000
C	1.428283	-1.721406	0.000000
C	1.279857	-0.338871	-0.000000
C	-0.000000	0.220839	-0.000000
H	-2.115860	-0.157197	0.000000

H	-1.845782	-2.623631	0.000000
H	0.419665	-3.621535	0.000000
H	2.418477	-2.157451	-0.000000
H	2.136915	0.321137	-0.000000
C	-0.106956	1.699617	-0.000000
O	0.833840	2.465027	-0.000000
O	-1.393431	2.129899	-0.000000
H	-1.338558	3.098289	-0.000000

CH_4

C	0.000000	0.000000	0.000000
H	0.627086	0.627086	0.627086
H	-0.627086	-0.627086	0.627086
H	0.627086	-0.627086	-0.627086
H	-0.627086	0.627086	-0.627086

CH_3F

C	0.000000	0.000000	-0.636546
H	0.000000	1.029888	-0.983809
H	-0.891909	-0.514944	-0.983809
H	0.891909	-0.514944	-0.983809
F	0.000000	0.000000	0.752301

CH_2F_2

C	0.000000	0.504943	0.000000
H	-0.000022	1.099614	0.909743
H	-0.000022	1.099614	-0.909743
F	-1.101784	-0.290547	0.000000
F	1.101789	-0.290441	0.000000

CHF_3

C	0.000000	0.000000	0.339441
H	0.000000	0.000000	1.424694
F	0.000000	1.251478	-0.128198
F	-1.083812	-0.625739	-0.128198
F	1.083812	-0.625739	-0.128198

CH_3Cl

C	0.000000	0.000000	-1.124062
H	0.000000	1.028418	-1.466189
H	-0.890636	-0.514209	-1.466189
H	0.890636	-0.514209	-1.466189
CL	0.000000	0.000000	0.655467

CH_2Cl_2

C	0.000000	0.769405	0.000000

H	-0.000020	1.374804	0.897060
H	-0.000020	1.374804	-0.897060
CL	-1.467387	-0.216656	0.000000
CL	1.467389	-0.216640	0.000000

$CHCl_3$

C	-0.000011	-0.000001	0.460759
H	-0.000026	0.000009	1.542595
CL	0.977671	-1.361963	-0.084463
CL	0.690682	1.527653	-0.084455
CL	-1.668347	-0.165691	-0.084443

CH_3Br

C	0.000000	0.000000	-1.508255
H	0.000000	1.030710	-1.841221
H	0.892621	-0.515355	-1.841221
H	-0.892621	-0.515355	-1.841221
BR	0.000000	0.000000	0.416377

CH_2Br_2

C	0.000000	0.932224	0.000000
H	-0.000030	1.535201	0.897498
H	-0.000030	1.535201	-0.897498
BR	-1.596296	-0.123762	0.000000
BR	1.596298	-0.123773	0.000000

$CHBr_3$

C	-0.000042	-0.000004	0.527437
H	-0.000125	0.000046	1.608515
BR	0.961168	-1.551868	-0.045498
BR	0.863462	1.608275	-0.045466
BR	-1.824620	-0.056408	-0.045411

CH_3I

C	0.000000	0.000000	-1.797610
H	0.000000	1.031584	-2.127179
H	-0.893378	-0.515792	-2.127179
H	0.893378	-0.515792	-2.127179
I	0.000000	0.000000	0.323909

CH_2I_2

C	0.000000	1.053211	0.000000
H	-0.000230	1.660924	0.894698
H	-0.000230	1.660924	-0.894698
I	-1.783537	-0.091077	0.000000
I	1.783546	-0.090831	0.000000

CHI_3

C	-0.000191	-0.000934	0.565126
H	-0.000286	-0.001843	1.647817
I	-1.837471	-0.889708	-0.031713
I	1.690010	-1.144914	-0.031706
I	0.147488	2.034762	-0.031649

CH_2FCl

C	0.000000	0.802028	0.000000
H	0.330701	1.303584	0.903005
H	0.330701	1.303584	-0.903005
F	-1.363875	0.739102	0.000000
CL	0.683145	-0.827721	0.000000

CH_2FBr

C	0.517466	-1.097590	0.000000
H	1.089946	-1.273822	0.903648
H	1.089946	-1.273822	-0.903648
BR	0.000000	0.749228	0.000000
F	-0.587187	-1.898865	0.000000

CH_2FI

C	0.525639	-1.454940	0.000000
H	1.097478	-1.638895	0.902540
H	1.097478	-1.638895	-0.902540
I	0.000000	0.606906	0.000000
F	-0.594310	-2.239844	0.000000

CH_2ClBr

C	0.000000	1.018163	0.000000
H	-0.314257	1.533723	0.897418
H	-0.314257	1.533723	-0.897418
BR	-0.837803	-0.706838	0.000000
CL	1.761859	0.915466	0.000000

CH_2ClI

C	0.796379	-1.062117	0.000000
H	1.394430	-1.163071	0.896369
H	1.394430	-1.163071	-0.896369
I	0.000000	0.907188	0.000000
CL	-0.445125	-2.316594	0.000000

CH_2BrI

C	0.000000	1.082411	0.000000
H	-0.226609	1.643285	0.896946

H -0.226609 1.643285 -0.896946
BR 1.875145 0.696201 0.000000
I -1.229752 -0.644303 0.000000

CHF_2Cl
C -0.435437 0.000010 0.366394
H -0.545067 -0.000035 1.445855
CL 1.273356 -0.000038 -0.051159
F -1.027262 -1.086011 -0.154123
F -1.027111 1.086081 -0.154155

CHF_2Br
C -0.424879 -0.895543 0.000000
H -1.507463 -0.967292 0.000000
F 0.076542 -1.501327 1.086874
F 0.076542 -1.501327 -1.086874
BR 0.076542 0.953270 0.000000

CHF2I.mp2.at.smd.mecn.sp.01.com.log
C -0.451880 -1.268581 0.000000
H -1.533299 -1.363430 0.000000
F 0.059783 -1.867784 1.089153
F 0.059783 -1.867784 -1.089153
I 0.059783 0.803680 0.000000

$CHCl_2F$
C -0.179415 0.519033 0.000000
H -1.068941 1.137220 0.000000
F 0.916172 1.311028 0.000000
CL -0.179415 -0.472079 1.452872
CL -0.179415 -0.472079 -1.452872

$CHCl_2Br$
C 0.671096 -0.123327 0.000000
H 1.571658 0.475701 0.000000
CL 0.671096 -1.119011 1.451631
CL 0.671096 -1.119011 -1.451631
BR -0.811871 1.094590 0.000000

$CHCl_2I$
C 0.908038 -0.423309 0.000000
H 1.811924 0.171129 0.000000
CL 0.908038 -1.419922 1.452941
CL 0.908038 -1.419922 -1.452941
I -0.719499 0.955586 0.000000

$CHBr_2F$

C	0.103236	0.779324	0.000000
H	1.002872	1.382741	0.000000
BR	0.103236	-0.290163	1.584570
BR	0.103236	-0.290163	-1.584570
F	-0.983200	1.583636	0.000000

$CHBr_2Cl$

C	-0.233935	0.595887	0.000000
H	-1.154977	1.162473	0.000000
BR	-0.233935	-0.486389	1.581292
BR	-0.233935	-0.486389	-1.581292
CL	1.113767	1.724085	0.000000

$CHBr_2I$

C	0.641216	0.002377	0.000000
H	1.562042	0.569643	0.000000
BR	0.641216	-1.077573	1.581348
BR	0.641216	-1.077573	-1.581348
I	-0.948953	1.412192	0.000000

CHI_2F

C	0.071908	0.950947	0.000000
H	0.981268	1.541314	0.000000
I	0.071908	-0.219566	1.775813
I	0.071908	-0.219566	-1.775813
F	-1.003885	1.780775	0.000000

CHI_2Cl

C	-0.165442	0.809555	0.000000
H	-1.098855	1.356995	0.000000
I	-0.165442	-0.375505	1.768693
I	-0.165442	-0.375505	-1.768693
CL	1.154610	1.975837	0.000000

CHI_2Br

C	0.329891	0.537899	0.000000
H	1.259400	1.091822	0.000000
I	0.329891	-0.642473	1.768280
I	0.329891	-0.642473	-1.768280
BR	-1.091634	1.822368	0.000000

CHBrClF

C	0.560722	0.455823	0.413939
H	0.604053	0.592349	1.487953
CL	1.807910	-0.682336	-0.067726

BR -1.189379 -0.185447 -0.028591
F 0.769491 1.640341 -0.202171

CHIClF
C 0.977474 0.470741 0.418519
H 1.051408 0.611388 1.490642
CL 2.136108 -0.755775 -0.070831
I -1.029715 -0.100318 -0.017110
F 1.260534 1.636574 -0.210091

CHIBrF
C -0.442586 0.734209 0.430932
H -0.475842 0.908113 1.500152
I 1.425280 -0.179124 -0.018198
BR -1.929962 -0.373490 -0.031807
F -0.539982 1.916929 -0.223113

CHIBrCl
C 0.437533 0.468464 0.510726
H 0.463037 0.506541 1.590705
CL 0.721115 2.093555 -0.104163
BR 1.823083 -0.734300 -0.044005
I -1.493493 -0.249194 -0.025361

Optimized Coordinates from G3MP2 Neutral Species (MP2=Full Step)

Benzoic Acid
C -1.139208 -0.592010 0.000000
C -0.992356 -1.977446 0.000000
C 0.279691 -2.551456 0.000000
C 1.413089 -1.736022 0.000000
C 1.276700 -0.351185 0.000000
C 0.000000 0.220010 -0.000000
H -2.125838 -0.140000 0.000000
H -1.874660 -2.612402 0.000000
H 0.387766 -3.633338 0.000000
H 2.404464 -2.181789 0.000000
H 2.143831 0.303022 -0.000000
C -0.091223 1.700120 -0.000000
O 0.862269 2.462050 -0.000000
O -1.376055 2.146574 -0.000000
H -1.305444 3.123453 -0.000000

CH_4
C 0.000000 0.000000 0.000000
H 0.628982 0.628982 0.628982

H	-0.628982	-0.628982	0.628982
H	0.628982	-0.628982	-0.628982
H	-0.628982	0.628982	-0.628982

CH_3F

C	0.000000	0.000000	-0.635463
H	0.000000	1.031385	-0.993324
H	-0.893205	-0.515692	-0.993324
H	0.893205	-0.515692	-0.993324
F	0.000000	0.000000	0.754750

CH_2F_2

C	0.000000	0.502952	0.000000
H	-0.000011	1.106679	0.908480
H	-0.000011	1.106679	-0.908480
F	-1.109667	-0.290620	0.000000
F	1.109669	-0.290610	0.000000

CHF_3

C	0.000000	0.000000	0.341090
H	0.000000	0.000000	1.429624
F	0.000000	1.258165	-0.128747
F	-1.089603	-0.629082	-0.128747
F	1.089603	-0.629082	-0.128747

CH_3Cl

C	0.000000	0.000000	-1.121193
H	0.000000	1.029052	-1.473626
H	-0.891185	-0.514526	-1.473626
H	0.891185	-0.514526	-1.473626
CL	0.000000	0.000000	0.655767

CH_2Cl_2

C	0.000000	0.760126	0.000000
H	0.000004	1.377398	0.894583
H	0.000004	1.377398	-0.894583
CL	-1.473782	-0.215162	0.000000
CL	1.473782	-0.215165	0.000000

$CHCl_3$

C	0.000000	0.000000	0.451777
H	0.000000	0.000000	1.537682
CL	0.000000	1.681683	-0.083301
CL	-1.456380	-0.840841	-0.083301
CL	1.456380	-0.840841	-0.083301

CH_3Br

C	0.000000	0.000000	-1.520303
H	0.000000	1.032651	-1.858136
H	0.894302	-0.516325	-1.858136
H	-0.894302	-0.516325	-1.858136
BR	0.000000	0.000000	0.419892

CH_2Br_2

C	0.000000	0.000000	0.933813
H	-0.898506	0.000000	1.542966
H	0.898506	0.000000	1.542966
BR	0.000000	1.614465	-0.124126
BR	0.000000	-1.614465	-0.124126

$CHBr_3$

C	-0.000007	-0.000001	0.525393
H	-0.000024	0.000010	1.610397
BR	0.840807	-1.641534	-0.045359
BR	1.001219	1.548920	-0.045359
BR	-1.842024	0.092613	-0.045360

CH_3I

C	0.000000	0.000000	-1.819404
H	0.000000	-1.035344	-2.150678
H	0.896634	0.517672	-2.150678
H	-0.896634	0.517672	-2.150678
I	0.000000	0.000000	0.327707

CH_2I_2

C	0.000000	1.060882	0.000000
H	0.000002	1.670377	0.898976
H	0.000002	1.670377	-0.898976
I	-1.805529	-0.091564	0.000000
I	1.805529	-0.091569	0.000000

CHI_3

C	-0.000028	-0.000011	0.570837
H	-0.000062	-0.000026	1.657737
I	2.013881	0.455315	-0.031862
I	-1.401256	1.516394	-0.031986
I	-0.612620	-1.971706	-0.032053

CH_2FCl

C	0.000000	0.801718	0.000000
H	0.332165	1.312367	0.902386
H	0.332165	1.312367	-0.902386

F	-1.368928	0.737203	0.000000
CL	0.685648	-0.827640	0.000000

CH_2FBr

C	-0.520456	-1.111300	0.000000
H	-1.097854	-1.288203	0.905030
H	-1.097854	-1.288203	-0.905030
BR	0.000000	0.754856	0.000000
F	0.590938	-1.908418	0.000000

CH_2FI

C	-0.529986	-1.479278	0.000000
H	-1.104557	-1.660306	0.906151
H	-1.104557	-1.660306	-0.906151
I	0.000000	0.613038	0.000000
F	0.598781	-2.254969	0.000000

CH_2ClBr

C	0.000000	1.022009	0.000000
H	0.333458	1.536305	0.896682
H	0.333458	1.536305	-0.896682
CL	-1.760219	0.952257	0.000000
BR	0.835909	-0.725515	0.000000

CH_2ClI

C	-0.791753	-1.085626	0.000000
H	-1.397032	-1.179125	0.897735
H	-1.397032	-1.179125	-0.897735
CL	0.443799	-2.340213	0.000000
I	0.000000	0.918031	0.000000

CH_2BrI

C	0.000000	1.092068	0.000000
H	-0.234538	1.654133	0.899214
H	-0.234538	1.654133	-0.899214
BR	1.891224	0.718053	0.000000
I	-1.240071	-0.660236	0.000000

CHF_2Cl

C	-0.434444	0.000002	0.368484
H	-0.546018	-0.000024	1.450892
CL	1.273850	0.000000	-0.051401
F	-1.027931	-1.092443	-0.154889
F	-1.027931	1.092444	-0.154886

CHF_2Br

C	-0.431165	0.906116	0.000000
H	-1.517076	0.977978	0.000000
F	0.077435	1.506366	-1.093290
F	0.077435	1.506366	1.093290
BR	0.077435	-0.957979	0.000000

CHF_2I

C	-0.456495	-1.290461	0.000000
H	-1.541552	-1.377964	0.000000
F	0.060289	-1.881311	1.097015
F	0.060289	-1.881311	-1.097015
I	0.060289	0.811025	0.000000

$CHCl_2F$

C	-0.181208	0.513402	0.000000
H	-1.069906	1.139941	0.000000
F	0.924248	1.303833	0.000000
CL	-0.181208	-0.469260	1.459857
CL	-0.181208	-0.469260	-1.459857

$CHCl_2Br$

C	0.671499	-0.141676	0.000000
H	1.574637	0.460621	0.000000
CL	0.671499	-1.130619	1.456547
CL	0.671499	-1.130619	-1.456547
BR	-0.812417	1.109443	0.000000

$CHCl_2I$

C	0.913266	-0.449639	0.000000
H	1.815864	0.155012	0.000000
CL	0.913266	-1.436944	1.456901
CL	0.913266	-1.436944	-1.456901
I	-0.723519	0.969791	0.000000

$CHBr_2F$

C	-0.104635	0.782854	0.000000
H	-1.003377	1.394478	0.000000
BR	-0.104635	-0.289842	-1.602502
BR	-0.104635	-0.289842	1.602502
F	0.995068	1.577481	0.000000

$CHBr_2Cl$

C	-0.230563	0.600966	0.000000
H	-1.164910	1.153015	0.000000
BR	-0.230563	-0.492725	1.596934
BR	-0.230563	-0.492725	-1.596934

CL 1.099275 1.748938 0.000000

$CHBr_2I$

C	0.647962	-0.006132	0.000000
H	1.569747	0.567522	0.000000
BR	0.647962	-1.090103	1.595860
BR	0.647962	-1.090103	-1.595860
I	-0.958771	1.429745	0.000000

CHI_2F

C	0.072924	0.960762	0.000000
H	0.982567	1.558142	0.000000
I	0.072924	-0.219879	1.800757
I	0.072924	-0.219879	-1.800757
F	-1.016678	1.776047	0.000000

CHI_2Cl

C	-0.162894	0.825466	0.000000
H	-1.111727	1.354089	0.000000
I	-0.162894	-0.380941	1.788629
I	-0.162894	-0.380941	-1.788629
CL	1.138578	2.004287	0.000000

CHI_2Br

C	0.330599	0.545371	0.000000
H	1.267847	1.094611	0.000000
I	0.330599	-0.650546	1.789764
I	0.330599	-0.650546	-1.789764
BR	-1.094141	1.845458	0.000000

CHBrClF

C	0.571903	0.448836	0.417461
H	0.611330	0.587164	1.495103
CL	1.824176	-0.680117	-0.069095
BR	-1.200453	-0.185053	-0.028683
F	0.773568	1.639853	-0.202372

CHIClF

C	0.999476	0.462958	0.423107
H	1.063718	0.606808	1.499278
CL	2.160268	-0.752878	-0.072605
I	-1.041720	-0.100208	-0.017091
F	1.269559	1.636153	-0.210867

CHIBrF

C	-0.453912	0.737106	0.438649

H	-0.482939	0.913429	1.511414
I	1.443425	-0.179377	-0.018303
BR	-1.953842	-0.374273	-0.032328
F	-0.545626	1.918945	-0.226863

CHIBrCl

C	0.450487	0.483607	0.507058
H	0.470726	0.513653	1.592566
CL	0.734866	2.105741	-0.104872
BR	1.840231	-0.742618	-0.043646
I	-1.510839	-0.249458	-0.024990

Optimized Coordinates from B3LYP/aug-cc-pVTZ Radical Species

$CH_3^{\bullet}$

C	0.000000	0.000000	0.000000
H	0.000000	1.078004	0.000000
H	0.933579	-0.539002	0.000000
H	-0.933579	-0.539002	0.000000

$CH_2F^{\bullet}$

C	-0.023484	0.656281	0.000000
H	0.176130	1.111605	0.957809
H	0.176130	1.111605	-0.957809
F	-0.023484	-0.684544	0.000000

$CHF_2^{\bullet}$

C	0.029846	0.509363	0.000000
H	-0.716312	1.300634	0.000000
F	0.029846	-0.242045	1.097203
F	0.029846	-0.242045	-1.097203

$CF_3^{\bullet}$

C	0.000000	0.000000	0.326146
F	0.000000	1.260436	-0.072477
F	-1.091570	-0.630218	-0.072477
F	1.091570	-0.630218	-0.072477

$CH_2Cl^{\bullet}$

C	-0.000090	-1.119841	0.000000
H	0.001035	-1.618902	0.952542
H	0.001035	-1.618902	-0.952542
CL	-0.000090	0.585697	0.000000

$CHCl_2^{\bullet}$

C	0.011449	0.693146	0.000000

H	-0.457973	1.662838	0.000000
CL	0.011449	-0.171227	1.476575
CL	0.011449	-0.171227	-1.476575

$CCl_3^{\bullet}$

C	0.000000	0.000000	0.274106
CL	0.000000	1.694857	-0.032248
CL	-1.467789	-0.847429	-0.032248
CL	1.467789	-0.847429	-0.032248

$CH_2Br^{\bullet}$

C	-0.000015	-1.490240	0.000000
H	0.000315	-1.989087	0.952607
H	0.000315	-1.989087	-0.952607
BR	-0.000015	0.369132	0.000000

$CHBr_2^{\bullet}$

C	0.006458	0.831902	0.000000
H	-0.490841	1.788409	0.000000
BR	0.006458	-0.096855	1.623165
BR	0.006458	-0.096855	-1.623165

$CBr_3^{\bullet}$

C	0.000000	0.000000	0.309957
BR	0.000000	1.860068	-0.017712
BR	-1.610866	-0.930034	-0.017712
BR	1.610866	-0.930034	-0.017712

$CH_2I^{\bullet}$

C	-0.000026	-1.768612	0.000000
H	0.000773	-2.276972	0.948295
H	0.000773	-2.276972	-0.948295
I	-0.000026	0.286144	0.000000

$CHI_2^{\bullet}$

C	0.003639	0.912926	0.000000
H	-0.407518	1.910508	0.000000
I	0.003639	-0.069699	1.822290
I	0.003639	-0.069699	-1.822290

$CI_3^{\bullet}$

C	-0.000418	0.000000	0.267576
I	1.040635	-1.800200	-0.010119
I	1.039110	1.801080	-0.010119
I	-2.079698	-0.000880	-0.010054

$CHFCl^{\bullet}$

C	0.542059	0.548029	-0.128909
H	0.724432	1.502070	0.348631
F	1.525749	-0.340177	0.026366
CL	-1.041678	-0.101685	0.011031

$CHFBr^{\bullet}$

C	1.049066	0.566613	-0.131245
H	1.276204	1.503522	0.363113
BR	-0.726268	-0.044698	0.005050
F	1.983199	-0.370976	0.027512

$CHFI^{\bullet}$

C	1.412474	0.571748	-0.127300
H	1.682644	1.503258	0.357487
I	-0.586106	-0.026500	0.003026
F	2.322905	-0.392139	0.027327

$CHClBr^{\bullet}$

C	0.585583	0.744727	-0.097299
H	0.646407	1.755257	0.273075
CL	2.005163	-0.200366	0.009587
BR	-1.092791	-0.080497	0.004221

$CHClI^{\bullet}$

C	1.002925	0.767873	-0.089481
H	1.116547	1.783902	0.253852
CL	2.393569	-0.220943	0.009110
I	-0.902354	-0.049719	0.002418

$CHBrI^{\bullet}$

C	-0.445096	0.864162	-0.092086
H	-0.498585	1.880020	0.267071
BR	-2.039079	-0.109305	0.004264
I	1.406357	-0.061120	0.002570

$CF_2Cl^{\bullet}$

C	0.427410	-0.000001	0.328261
CL	-1.276475	-0.000020	-0.031479
F	1.063048	1.086264	-0.079689
F	1.063131	-1.086224	-0.079691

$CF_2Br^{\bullet}$

C	-0.364088	-0.918047	0.000000
F	0.041217	-1.558632	1.082684
F	0.041217	-1.558632	-1.082684

BR 0.041217 0.958962 0.000000

$CF_2I^•$

C -0.379548 -1.302162 0.000000
F 0.032074 -1.946113 1.079242
F 0.032074 -1.946113 -1.079242
I 0.032074 0.808358 0.000000

$CCl_2F^•$

C -0.158849 0.436688 0.000000
F 0.705996 1.444936 0.000000
CL -0.158849 -0.459546 1.480022
CL -0.158849 -0.459546 -1.480022

$CCl_2Br^•$

C 0.440717 -0.355225 0.000000
CL 0.440717 -1.259063 1.464939
CL 0.440717 -1.259063 -1.464939
BR -0.503676 1.283985 0.000000

$CCl_2I^•$

C 0.560013 -0.726674 0.000000
CL 0.560013 -1.637669 1.461868
CL 0.560013 -1.637669 -1.461868
I -0.422651 1.132845 0.000000

$CBr_2F^•$

C 0.091347 0.698512 0.000000
BR 0.091347 -0.279029 1.632933
BR 0.091347 -0.279029 -1.632933
F -0.771378 1.704550 0.000000

$CBr_2Cl^•$

C -0.156334 0.468167 0.000000
BR -0.156334 -0.516009 1.614353
BR -0.156334 -0.516009 -1.614353
CL 0.698904 1.959506 0.000000

$CBr_2I^•$

C 0.392953 -0.228100 0.000000
BR 0.392953 -1.222990 1.606301
BR 0.392953 -1.222990 -1.606301
I -0.563479 1.641093 0.000000

$CI_2F^•$

C 0.061646 0.851893 0.000000

I	0.061646	-0.208640	1.839123
I	0.061646	-0.208640	-1.839123
F	-0.767153	1.889383	0.000000

$CI_2Cl^{\bullet}$

C	-0.099211	0.670949	0.000000
I	-0.099211	-0.393701	1.811831
I	-0.099211	-0.393701	-1.811831
CL	0.653627	2.218033	0.000000

$CI_2Br^{\bullet}$

C	0.192553	0.363643	0.000000
I	0.192553	-0.704446	1.807058
I	0.192553	-0.704446	-1.807058
BR	-0.616170	2.071126	0.000000

$CBrClF^{\bullet}$

C	0.565765	0.420478	0.328511
CL	1.869648	-0.655354	-0.036223
BR	-1.207500	-0.180761	-0.015798
F	0.787099	1.660531	-0.089148

$CIClF^{\bullet}$

C	0.984321	0.432803	0.328921
CL	2.220016	-0.722311	-0.037405
I	-1.043229	-0.098300	-0.009686
F	1.293882	1.654709	-0.091585

$CIBrF^{\bullet}$

C	-0.444768	0.682845	0.340447
I	1.466601	-0.171334	-0.010352
BR	-2.001710	-0.353872	-0.017446
F	-0.555709	1.929910	-0.098157

$CIBrCl^{\bullet}$

C	0.433927	0.459432	0.288140
CL	0.740123	2.120719	-0.037116
BR	1.871520	-0.732084	-0.016403
I	-1.522431	-0.248790	-0.009883

Optimized Coordinates from MP2/aug-cc-pVTZ Radical Species

$CH_3^{\bullet}$

C	0.000000	0.000000	0.000000
H	0.000000	1.075093	0.000000
H	0.931058	-0.537546	0.000000

H	-0.931058	-0.537546	0.000000

$CH_2F^{\bullet}$

C	-0.028793	0.657784	0.000000
H	0.215946	1.101848	0.949663
H	0.215946	1.101848	-0.949663
F	-0.028793	-0.683378	0.000000

$CHF_2^{\bullet}$

C	0.030240	0.510648	0.000000
H	-0.725750	1.287602	0.000000
F	0.030240	-0.241750	1.093950
F	0.030240	-0.241750	-1.093950

$CF_3^{\bullet}$

C	0.000000	0.000000	0.326458
F	0.000000	1.255537	-0.072546
F	-1.087327	-0.627769	-0.072546
F	1.087327	-0.627769	-0.072546

$CH_2Cl^{\bullet}$

C	0.007840	1.113122	0.000000
H	-0.090165	1.608372	0.947418
H	-0.090165	1.608372	-0.947418
CL	0.007840	-0.582087	0.000000

$CHCl_2^{\bullet}$

C	0.012858	0.701277	0.000000
H	-0.514303	1.640547	0.000000
CL	0.012858	-0.172006	1.459287
CL	0.012858	-0.172006	-1.459287

$CCl_3^{\bullet}$

C	0.000000	0.000000	0.297389
CL	0.000000	1.677727	-0.034987
CL	-1.452954	-0.838864	-0.034987
CL	1.452954	-0.838864	-0.034987

$CH_2Br^{\bullet}$

C	-0.005850	1.472294	0.000000
H	0.119918	1.965195	0.945904
H	0.119918	1.965195	-0.945904
BR	-0.005850	-0.364690	0.000000

$CHBr_2^{\bullet}$

C	0.006854	0.835012	0.000000

H	-0.520893	1.775002	0.000000
BR	0.006854	-0.096930	1.591589
BR	0.006854	-0.096930	-1.591589

$CBr_3^{\bullet}$

C	0.000000	0.000000	0.320494
BR	0.000000	1.826851	-0.018314
BR	-1.582099	-0.913425	-0.018314
BR	1.582099	-0.913425	-0.018314

$CH_2I^{\bullet}$

C	0.002127	1.745576	0.000000
H	-0.062756	2.257261	0.942777
H	-0.062756	2.257261	-0.942777
I	0.002127	-0.282792	0.000000

$CHI_2^{\bullet}$

C	0.003753	0.916629	0.000000
H	-0.420289	1.908498	0.000000
I	0.003753	-0.069889	1.784553
I	0.003753	-0.069889	-1.784553

$CI_3^{\bullet}$

C	0.000000	0.000000	0.257385
I	0.000000	2.039922	-0.009713
I	-1.766624	-1.019961	-0.009713
I	1.766624	-1.019961	-0.009713

$CHFCl^{\bullet}$

C	0.535406	0.549377	-0.132870
H	0.713657	1.493317	0.361612
CL	-1.033749	-0.102240	0.011344
F	1.516404	-0.339055	0.026974

$CHFBr^{\bullet}$

C	1.027686	0.567719	-0.133463
H	1.251534	1.498033	0.370328
BR	-0.716465	-0.045304	0.005174
F	1.962071	-0.368744	0.027705

$CHFI^{\bullet}$

C	1.385409	0.572499	-0.128291
H	1.654163	1.499872	0.360640
I	-0.578062	-0.026871	0.003083
F	2.296740	-0.390078	0.027300

CHClBr•

C	0.570680	0.749698	-0.105826
H	0.631299	1.746108	0.300103
CL	1.974306	-0.200266	0.010279
BR	-1.074817	-0.081136	0.004575

CHClI•

C	0.981661	0.773127	-0.096963
H	1.097491	1.777449	0.277563
CL	2.354481	-0.221317	0.009733
I	-0.887050	-0.050072	0.002618

CHBrI•

C	-0.436900	0.866400	-0.096227
H	-0.491915	1.875369	0.280417
BR	-1.998154	-0.109527	0.004431
I	1.378278	-0.061138	0.002677

$CF_2Cl^•$

C	0.419950	-0.000101	0.329903
CL	-1.264166	-0.000018	-0.031845
F	1.053895	1.083945	-0.079878
F	1.054008	-1.083843	-0.079905

$CF_2Br^•$

C	-0.363275	-0.894587	0.000000
F	0.041125	-1.534474	1.081659
F	0.041125	-1.534474	-1.081659
BR	0.041125	0.942516	0.000000

$CF_2I^•$

C	-0.375708	-1.267973	0.000000
F	0.031750	-1.912080	1.079531
F	0.031750	-1.912080	-1.079531
I	0.031750	0.792930	0.000000

$CCl_2F^•$

C	-0.161870	0.439120	0.000000
F	0.719420	1.433281	0.000000
CL	-0.161870	-0.456890	1.462358
CL	-0.161870	-0.456890	-1.462358

$CCl_2Br^•$

C	0.463301	-0.324966	0.000000
CL	0.463301	-1.229486	1.450614
CL	0.463301	-1.229486	-1.450614

BR	-0.529486	1.250066	0.000000

$CCl_2I^•$

C	0.584495	-0.689136	0.000000
CL	0.584495	-1.598834	1.447312
CL	0.584495	-1.598834	-1.447312
I	-0.441128	1.103683	0.000000

$CBr_2F^•$

C	0.092048	0.689518	0.000000
BR	0.092048	-0.276713	1.598255
BR	0.092048	-0.276713	-1.598255
F	-0.777296	1.692533	0.000000

$CBr_2Cl^•$

C	-0.164314	0.468033	0.000000
BR	-0.164314	-0.506237	1.585032
BR	-0.164314	-0.506237	-1.585032
CL	0.734581	1.919318	0.000000

$CBr_2I^•$

C	0.398982	-0.214560	0.000000
BR	0.398982	-1.195318	1.577797
BR	0.398982	-1.195318	-1.577797
I	-0.572126	1.603011	0.000000

$CI_2F^•$

C	0.061331	0.839474	0.000000
I	0.061331	-0.207576	1.794518
I	0.061331	-0.207576	-1.794518
F	-0.763233	1.885132	0.000000

$CI_2Cl^•$

C	-0.102141	0.661196	0.000000
I	-0.102141	-0.387536	1.775294
I	-0.102141	-0.387536	-1.775294
CL	0.672929	2.183035	0.000000

$CI_2Br^•$

C	0.192371	0.358092	0.000000
I	0.192371	-0.690765	1.771840
I	0.192371	-0.690765	-1.771840
BR	-0.615588	2.030644	0.000000

$CBrClF^•$

C	0.550920	0.416382	0.332393

CL	1.838460	-0.651957	-0.036601
BR	-1.185960	-0.180510	-0.016119
F	0.772139	1.655872	-0.089776

$CIClF^{\bullet}$

C	0.961934	0.428991	0.330025
CL	2.176480	-0.721802	-0.037441
I	-1.023759	-0.097239	-0.009807
F	1.276384	1.650037	-0.091542

$CIBrF^{\bullet}$

C	-0.435182	0.672708	0.338403
I	1.433446	-0.169578	-0.010370
BR	-1.954662	-0.353107	-0.017434
F	-0.549815	1.923348	-0.096738

$CIBrCl^{\bullet}$

C	0.424645	0.448137	0.299225
CL	0.729038	2.094191	-0.038484
BR	1.834357	-0.723588	-0.017082
I	-1.493283	-0.244613	-0.010250

Optimized Coordinates from G3MP2 Radical Species (MP2=Full Step)

$CH_3^{\bullet}$

C	0.000000	0.000000	0.000002
H	0.000000	1.078275	-0.000003
H	-0.933813	-0.539137	-0.000003
H	0.933813	-0.539137	-0.000003

$CH_2F^{\bullet}$

F	0.032640	-0.687513	0.000000
C	0.032640	0.662381	0.000000
H	-0.244804	1.106664	0.946176
H	-0.244804	1.106664	-0.946176

$CHF_2^{\bullet}$

C	0.030562	0.511096	0.000000
H	-0.733500	1.286092	0.000000
F	0.030562	-0.241815	1.104608
F	0.030562	-0.241815	-1.104608

$CF_3^{\bullet}$

C	0.000000	0.000000	0.329869
F	0.000000	1.264475	-0.073304
F	-1.095068	-0.632238	-0.073304

F	1.095068	-0.632238	-0.073304

$CH_2Cl^•$

C	0.010951	1.116765	0.000000
H	-0.125934	1.613613	0.946860
H	-0.125934	1.613613	-0.946860
CL	0.010951	-0.583989	0.000000

$CHCl_2^•$

C	0.012460	0.693470	0.000000
H	-0.498409	1.645699	0.000000
CL	0.012460	-0.170780	1.467822
CL	0.012460	-0.170780	-1.467822

$CCl_3^•$

C	0.000000	0.000000	0.273187
CL	0.000000	1.683418	-0.032140
CL	-1.457883	-0.841709	-0.032140
CL	1.457883	-0.841709	-0.032140

$CH_2Br^•$

C	-0.007499	1.487692	0.000000
H	0.153725	1.978673	0.946449
H	0.153725	1.978673	-0.946449
BR	-0.007499	-0.368100	0.000000

$CHBr_2^•$

C	0.006652	0.834788	0.000000
H	-0.505525	1.787592	0.000000
BR	0.006652	-0.097090	1.608257
BR	0.006652	-0.097090	-1.608257

$CBr_3^•$

C	0.000000	0.000000	0.296226
BR	0.000000	1.843415	-0.016927
BR	-1.596445	-0.921708	-0.016927
BR	1.596445	-0.921708	-0.016927

$CH_2I^•$

C	-0.004973	-1.767235	0.000000
H	0.146701	-2.266938	0.944608
H	0.146701	-2.266938	-0.944608
I	-0.004973	0.285609	0.000000

$CHI_2^•$

C	0.004148	0.927974	0.000000

H	-0.464539	1.904761	0.000000
I	0.004148	-0.070496	1.802678
I	0.004148	-0.070496	-1.802678

$CI_3^{\bullet}$

C	0.000000	-0.000037	0.264981
I	-1.784460	-1.030241	-0.009999
I	1.784459	-1.030243	-0.009999
I	0.000001	2.060488	-0.009999

$CHFCl^{\bullet}$

C	-0.535466	0.552493	-0.135216
H	-0.711364	1.496712	0.368917
F	-1.523547	-0.339779	0.027373
CL	1.037417	-0.103156	0.011530

$CHFBr^{\bullet}$

C	1.039865	0.572879	-0.136134
H	1.260906	1.501937	0.380082
BR	-0.721935	-0.045386	0.005214
F	1.974182	-0.372300	0.028247

$CHFI^{\bullet}$

C	1.407644	0.580191	-0.135475
H	1.663860	1.499021	0.385953
I	-0.583591	-0.026877	0.003162
F	2.313401	-0.395075	0.028810

$CHClBr^{\bullet}$

C	0.581682	0.744921	-0.102899
H	0.639973	1.750468	0.289581
CL	1.990847	-0.200208	0.010126
BR	-1.084985	-0.080470	0.004448

$CHClI^{\bullet}$

C	0.998456	0.770912	-0.098323
H	1.106092	1.779096	0.280692
CL	2.374184	-0.221667	0.009993
I	-0.895433	-0.049740	0.002629

$CHBrI^{\bullet}$

C	-0.444272	0.872226	-0.098740
H	-0.494788	1.881917	0.288635
BR	-2.017364	-0.110286	0.004553
I	1.391852	-0.061420	0.002726

$CF_2Cl^•$

C	-0.420414	0.000006	0.334415
CL	1.266265	-0.000005	-0.032295
F	-1.055787	-1.092321	-0.080970
F	-1.055770	1.092326	-0.080971

$CF_2Br^•$

C	-0.371126	0.905067	0.000000
F	0.042014	1.540927	-1.090266
F	0.042014	1.540927	1.090266
BR	0.042014	-0.947631	0.000000

$CF_2I^•$

C	-0.385933	-1.291083	0.000000
F	0.032614	-1.927191	1.088727
F	0.032614	-1.927191	-1.088727
I	0.032614	0.800678	0.000000

$CCl_2F^•$

C	-0.162977	0.433241	0.000000
F	0.724341	1.433646	0.000000
CL	-0.162977	-0.455949	1.468904
CL	-0.162977	-0.455949	-1.468904

$CCl_2Br^•$

C	0.433288	-0.355090	0.000000
CL	0.433288	-1.248961	1.456617
CL	0.433288	-1.248961	-1.456617
BR	-0.495186	1.274149	0.000000

$CCl_2I^•$

C	0.557010	-0.721557	0.000000
CL	0.557010	-1.620808	1.453603
CL	0.557010	-1.620808	-1.453603
I	-0.420385	1.121449	0.000000

$CBr_2F^•$

C	-0.093261	0.690729	0.000000
BR	-0.093261	-0.276844	-1.615206
BR	-0.093261	-0.276844	1.615206
F	0.787533	1.692749	0.000000

$CBr_2Cl^•$

C	-0.151693	0.462676	0.000000
BR	-0.151693	-0.514237	1.597830
BR	-0.151693	-0.514237	-1.597830

CL	0.678157	1.954150	0.000000

$CBr_2I^•$

C	0.382490	-0.231602	0.000000
BR	0.382490	-1.212691	1.591337
BR	0.382490	-1.212691	-1.591337
I	-0.548477	1.627887	0.000000

$CI_2F^•$

C	0.064136	0.857861	0.000000
I	0.064136	-0.207949	1.817469
I	0.064136	-0.207949	-1.817469
F	-0.798131	1.877274	0.000000

$CI_2Cl^•$

C	-0.098072	0.672770	0.000000
I	-0.098072	-0.392300	1.790704
I	-0.098072	-0.392300	-1.790704
CL	0.646124	2.208655	0.000000

$CI_2Br^•$

C	0.188906	0.361501	0.000000
I	0.188906	-0.698565	1.789316
I	0.188906	-0.698565	-1.789316
BR	-0.604500	2.053681	0.000000

$CBrClF^•$

C	0.559845	0.410804	0.334270
CL	1.853768	-0.651361	-0.037233
BR	-1.195944	-0.180613	-0.016167
F	0.776102	1.658862	-0.089647

$CIClF^•$

C	0.980263	0.423808	0.338465
CL	2.199154	-0.720373	-0.038991
I	-1.034204	-0.097642	-0.009950
F	1.282848	1.653170	-0.093400

$CIBrF^•$

C	-0.445207	0.674973	0.349582
I	1.450215	-0.170138	-0.010634
BR	-1.977343	-0.353826	-0.018101
F	-0.553685	1.927928	-0.100038

$CIBrCl^•$

C	0.432496	0.458763	0.282545

CL	0.737776	2.107704	-0.036668
BR	1.847970	-0.730325	-0.016080
I	-1.505965	-0.245702	-0.009606

Optimized Coordinates from B3LYP/aug-cc-pVTZ Anion Species

Benzoate

C	0.000000	1.197883	0.437451
C	0.000000	1.202318	1.829057
C	0.000000	0.000000	2.532244
C	-0.000000	-1.202318	1.829057
C	-0.000000	-1.197883	0.437451
C	-0.000000	-0.000000	-0.277850
H	0.000000	2.116715	-0.133617
H	0.000000	2.142673	2.369172
H	0.000000	0.000000	3.616060
H	-0.000000	-2.142673	2.369172
H	-0.000000	-2.116715	-0.133617
C	-0.000000	-0.000000	-1.828245
O	-0.000000	-1.131493	-2.365135
O	0.000000	1.131493	-2.365135

CH_3^-

C	0.000000	0.000000	0.121134
H	0.000000	1.037299	-0.242267
H	-0.898327	-0.518650	-0.242267
H	0.898327	-0.518650	-0.242267

CH_2F^-

C	0.752539	0.000017	-0.173664
H	1.064399	0.877176	0.430833
H	1.064651	-0.877139	0.430828
F	-0.738254	-0.000016	0.020036

CHF_2^-

C	0.043153	0.663298	0.000000
H	-1.035679	0.944486	0.000000
F	0.043153	-0.273571	1.134046
F	0.043153	-0.273571	-1.134046

CF_3^-

C	0.000000	0.000000	0.547981
F	0.000000	1.267885	-0.121774
F	-1.098021	-0.633943	-0.121774
F	1.098021	-0.633943	-0.121774

CH_2Cl^-

C	0.052905	1.329187	0.000000
H	-0.608403	1.492758	0.868756
H	-0.608403	1.492758	-0.868756
CL	0.052905	-0.644743	0.000000

$CHCl_2^-$

C	0.026357	0.971736	0.000000
H	-1.054289	1.177990	0.000000
CL	0.026357	-0.206130	1.538797
CL	0.026357	-0.206130	-1.538797

CCl_3^-

C	0.000000	0.000000	0.732116
CL	0.000000	1.735790	-0.086131
CL	-1.503238	-0.867895	-0.086131
CL	1.503238	-0.867895	-0.086131

CH_2Br^-

C	-0.031227	1.738924	0.000000
H	0.640147	1.875801	0.866538
H	0.640147	1.875801	-0.866538
BR	-0.031227	-0.405290	0.000000

$CHBr_2^-$

C	0.014142	1.145352	0.000000
H	-1.074786	1.316700	0.000000
BR	0.014142	-0.116983	1.686762
BR	0.014142	-0.116983	-1.686762

CBr_3^-

C	0.000000	0.000000	0.816633
BR	0.000000	1.906921	-0.046665
BR	-1.651442	-0.953461	-0.046665
BR	1.651442	-0.953461	-0.046665

CH_2I^-

C	0.022113	2.020637	0.000000
H	-0.652332	2.150552	0.865885
H	-0.652332	2.150552	-0.865885
I	0.022113	-0.309904	0.000000

CHI_2^-

C	0.009664	1.261923	0.000000
H	-1.082331	1.428678	0.000000

I	0.009664	-0.084908	1.873830
I	0.009664	-0.084908	-1.873830

CI_3^-

C	0.000359	-0.000022	0.857066
I	-1.042363	1.855260	-0.032312
I	-1.085878	-1.830140	-0.032305
I	2.128200	-0.025118	-0.032409

$CHFCl^-$

C	0.726794	0.711913	-0.195715
H	0.707440	1.158929	0.819320
F	1.580418	-0.412769	0.027691
CL	-1.134822	-0.100911	0.006221

$CHFBr^-$

C	1.315726	0.720022	-0.193619
H	1.275374	1.141965	0.832865
BR	-0.800449	-0.042417	0.002195
F	2.093998	-0.441945	0.028004

$CHFI^-$

C	1.738000	0.716788	-0.192589
H	1.681275	1.129041	0.839156
I	-0.649153	-0.024613	0.001124
F	2.477317	-0.458367	0.028532

$CHClBr^-$

C	0.662720	1.023051	-0.185096
H	0.655472	1.379682	0.856853
CL	2.083235	-0.247408	0.008469
BR	-1.144194	-0.094630	0.003136

$CHClI^-$

C	1.129341	1.049751	-0.184008
H	1.114816	1.397854	0.862274
CL	2.479517	-0.273841	0.008829
I	-0.944201	-0.057379	0.001730

$CHBrI^-$

C	-0.494304	1.181815	-0.181938
H	-0.487793	1.503085	0.873841
BR	-2.107532	-0.134335	0.003452
I	1.456929	-0.073439	0.001830

CF_2Cl^-

C	-0.711148	-0.000004	0.584392
CL	1.467783	-0.000002	-0.034524
F	-1.149196	-1.061832	-0.162192
F	-1.149185	1.061839	-0.162190

CF_2Br^-

C	-0.653523	-1.304908	0.000000
F	0.073984	-1.752273	1.055531
F	0.073984	-1.752273	-1.055531
BR	0.073984	1.124868	0.000000

CF_2I^-

C	-0.672158	-1.829223	0.000000
F	0.056802	-2.252028	1.050112
F	0.056802	-2.252028	-1.050112
I	0.056802	0.971922	0.000000

CCl_2F^-

C	-0.237430	0.802810	0.000000
F	1.055245	1.220987	0.000000
CL	-0.237430	-0.464875	1.568446
CL	-0.237430	-0.464875	-1.568446

CCl_2Br^-

C	0.924788	0.158257	0.000000
CL	0.924788	-1.018647	1.492237
CL	0.924788	-1.018647	-1.492237
BR	-1.056901	0.962413	0.000000

CCl_2I^-

C	1.260851	-0.126187	0.000000
CL	1.260851	-1.286171	1.481558
CL	1.260851	-1.286171	-1.481558
I	-0.951586	0.839376	0.000000

CBr_2F^-

C	0.134084	1.082960	0.000000
BR	0.134084	-0.288319	1.741295
BR	0.134084	-0.288319	-1.741295
F	-1.132264	1.520509	0.000000

CBr_2Cl^-

C	-0.316672	0.849965	0.000000
BR	-0.316672	-0.456121	1.666039
BR	-0.316672	-0.456121	-1.666039
CL	1.415708	1.578156	0.000000

CBr_2I^-

C	0.874818	0.282346	0.000000
BR	0.874818	-0.989012	1.641196
BR	0.874818	-0.989012	-1.641196
I	-1.254456	1.274278	0.000000

CI_2F^-

C	0.092285	1.267583	0.000000
I	0.092285	-0.220013	1.957316
I	0.092285	-0.220013	-1.957316
F	-1.148432	1.746214	0.000000

CI_2Cl^-

C	-0.220925	1.067275	0.000000
I	-0.220925	-0.358259	1.871664
I	-0.220925	-0.358259	-1.871664
CL	1.455507	1.857165	0.000000

CI_2Br^-

C	0.441421	0.797809	0.000000
I	0.441421	-0.605893	1.855143
I	0.441421	-0.605893	-1.855143
BR	-1.412547	1.698222	0.000000

$CBrClF^-$

C	0.702954	0.626363	0.643369
CL	1.973457	-0.713880	-0.060615
BR	-1.303334	-0.183866	-0.023485
F	0.872244	1.645897	-0.223089

$CIClF^-$

C	1.232704	0.628326	0.639927
CL	2.362553	-0.788545	-0.063723
I	-1.144449	-0.094375	-0.013333
F	1.455129	1.626356	-0.227735

$CIBrF^-$

C	-0.559344	0.954627	0.648635
I	1.577686	-0.178757	-0.014598
BR	-2.129411	-0.393136	-0.027023
F	-0.636877	1.945120	-0.241366

$CIBrCl^-$

C	0.509617	0.541074	0.787812
CL	0.787038	2.151596	-0.110891

BR	1.926728	-0.760956	-0.044147
I	-1.582506	-0.248870	-0.024464

Optimized Coordinates from MP2/aug-cc-pVTZ Anion Species

Benzoate

C	-0.000000	1.201648	0.432577
C	0.000000	1.205472	1.827216
C	0.000000	-0.000000	2.532461
C	-0.000000	-1.205472	1.827216
C	-0.000000	-1.201648	0.432577
C	-0.000000	0.000000	-0.281504
H	-0.000000	2.118657	-0.143513
H	0.000000	2.145354	2.368633
H	0.000000	-0.000000	3.616485
H	-0.000000	-2.145354	2.368633
H	-0.000000	-2.118657	-0.143513
C	-0.000000	0.000000	-1.824043
O	-0.000000	-1.138222	-2.359106
O	0.000000	1.138222	-2.359106

CH_3^-

C	0.000000	0.000000	0.121134
H	0.000000	1.037299	-0.242267
H	-0.898327	-0.518650	-0.242267
H	0.898327	-0.518650	-0.242267

CH_2F^-

C	-0.075040	0.761235	0.000000
H	0.562799	0.998670	0.874436
H	0.562799	0.998670	-0.874436
F	-0.075040	-0.729417	0.000000

CHF_2^-

C	0.043056	0.660471	0.000000
H	-1.033341	0.941186	0.000000
F	0.043056	-0.272445	1.126965
F	0.043056	-0.272445	-1.126965

CF_3^-

C	0.000000	0.000000	0.547835
F	0.000000	1.260536	-0.121741
F	-1.091656	-0.630268	-0.121741
F	1.091656	-0.630268	-0.121741

CH_2Cl^-

C	0.051729	1.273703	0.000000
H	-0.594883	1.473900	0.870296
H	-0.594883	1.473900	-0.870296
CL	0.051729	-0.622942	0.000000

$CHCl_2^-$

C	0.026113	0.947559	0.000000
H	-1.044521	1.199140	0.000000
CL	0.026113	-0.202485	1.488188
CL	0.026113	-0.202485	-1.488188

CCl_3^-

C	0.000000	0.000000	0.718730
CL	0.000000	1.690806	-0.084556
CL	-1.464281	-0.845403	-0.084556
CL	1.464281	-0.845403	-0.084556

CH_2Br^-

C	-0.030574	1.650364	0.000000
H	0.626762	1.830653	0.868055
H	0.626762	1.830653	-0.868055
BR	-0.030574	-0.387528	0.000000

$CHBr_2^-$

C	0.014018	1.116981	0.000000
H	-1.065398	1.340921	0.000000
BR	0.014018	-0.114897	1.610761
BR	0.014018	-0.114897	-1.610761

CBr_3^-

C	0.000000	0.000000	0.814089
BR	0.000000	1.839695	-0.046519
BR	-1.593222	-0.919847	-0.046519
BR	1.593222	-0.919847	-0.046519

CH_2I^-

C	0.021586	1.914419	0.000000
H	-0.636778	2.090171	0.868476
H	-0.636778	2.090171	-0.868476
I	0.021586	-0.295601	0.000000

CHI_2^-

C	0.009581	1.233941	0.000000
H	-1.073050	1.459132	0.000000
I	0.009581	-0.083611	1.784368
I	0.009581	-0.083611	-1.784368

CI_3^-

C	0.000580	-0.000004	0.871282
I	-1.018314	1.783755	-0.032869
I	-1.036076	-1.773503	-0.032869
I	2.054324	-0.010252	-0.032898

$CHFCl^-$

C	0.652727	0.709904	-0.196768
H	0.667648	1.184230	0.802774
F	1.545687	-0.400516	0.027242
CL	-1.087953	-0.108177	0.007803

$CHFBr^-$

C	1.186296	0.723154	-0.195252
H	1.197137	1.176431	0.814977
F	2.009595	-0.430788	0.027459
BR	-0.754322	-0.046808	0.003126

$CHFI^-$

C	1.565165	0.723615	-0.194566
H	1.571404	1.171451	0.820504
F	2.351094	-0.450043	0.028107
I	-0.606080	-0.027599	0.001772

$CHClBr^-$

C	0.610385	0.999075	-0.187650
H	0.617992	1.409725	0.833928
CL	2.002274	-0.240005	0.009336
BR	-1.094827	-0.094974	0.003808

$CHClI^-$

C	1.039948	1.029717	-0.186717
H	1.050849	1.435744	0.838626
CL	2.373700	-0.265632	0.009652
I	-0.898933	-0.058458	0.002219

$CHBrI^-$

C	-0.458263	1.153876	-0.184966
H	-0.461150	1.536632	0.850962
BR	-2.009473	-0.131375	0.003998
I	1.387590	-0.072863	0.002244

CF_2Cl^-

C	-0.600226	0.000005	0.583691
CL	1.359772	-0.000015	-0.040792

F	-1.084185	-1.070625	-0.156038
F	-1.084123	1.070650	-0.156037

CF_2Br^-

C	-0.649358	-1.145469	0.000000
F	0.073512	-1.630161	1.062561
F	0.073512	-1.630161	-1.062561
BR	0.073512	1.034734	0.000000

CF_2I^-

C	-0.671271	-1.717303	0.000000
F	0.056727	-2.143023	1.048410
F	0.056727	-2.143023	-1.048410
I	0.056727	0.922231	0.000000

CCl_2F^-

C	-0.241877	0.765433	0.000000
F	1.075007	1.195377	0.000000
CL	-0.241877	-0.451500	1.493040
CL	-0.241877	-0.451500	-1.493040

CCl_2Br^-

C	0.889962	0.178394	0.000000
CL	0.889962	-0.979890	1.460300
CL	0.889962	-0.979890	-1.460300
BR	-1.017100	0.921311	0.000000

CCl_2I^-

C	1.196104	-0.073522	0.000000
CL	1.196104	-1.227921	1.460115
CL	1.196104	-1.227921	-1.460115
I	-0.902720	0.796046	0.000000

CBr_2F^-

C	0.137428	1.042015	0.000000
BR	0.137428	-0.280274	1.630337
BR	0.137428	-0.280274	-1.630337
F	-1.160506	1.485229	0.000000

CBr_2Cl^-

C	-0.315468	0.828652	0.000000
BR	-0.315468	-0.438671	1.596810
BR	-0.315468	-0.438671	-1.596810
CL	1.410327	1.513828	0.000000

CBr_2I^-

C	0.852069	0.317615	0.000000
BR	0.852069	-0.936964	1.592086
BR	0.852069	-0.936964	-1.592086
I	-1.221835	1.201543	0.000000

CI_2F^-

C	0.093896	1.281827	0.000000
I	0.093896	-0.219964	1.928869
I	0.093896	-0.219964	-1.928869
F	-1.168483	1.736135	0.000000

CI_2Cl^-

C	-0.223498	1.040543	0.000000
I	-0.223498	-0.344583	1.783395
I	-0.223498	-0.344583	-1.783395
CL	1.472458	1.781326	0.000000

CI_2Br^-

C	0.440909	0.797945	0.000000
I	0.440909	-0.577586	1.779779
I	0.440909	-0.577586	-1.779779
BR	-1.410910	1.612471	0.000000

$CBrClF^-$

C	0.627541	0.584401	0.644670
CL	1.864429	-0.697396	-0.061999
BR	-1.221410	-0.184851	-0.025554
F	0.809868	1.646566	-0.213295

$CIClF^-$

C	1.094969	0.598234	0.645480
CL	2.206014	-0.775996	-0.064467
I	-1.059504	-0.096470	-0.014963
F	1.342409	1.635050	-0.220437

$CIBrF^-$

C	-0.493382	0.908251	0.657771
I	1.466632	-0.178610	-0.015927
BR	-1.986868	-0.384301	-0.028092
F	-0.581204	1.940821	-0.235476

$CIBrCl^-$

C	0.465817	0.509673	0.787781
CL	0.736141	2.109846	-0.108265
BR	1.842872	-0.737541	-0.044307
I	-1.505846	-0.247386	-0.025197

Optimized Coordinates from G3MP2 Anion Species (MP2=Full Step)

Benzoate

C	0.000000	1.202389	0.433130
C	0.000000	1.206175	1.828087
C	0.000000	-0.000000	2.533679
C	-0.000000	-1.206175	1.828087
C	-0.000000	-1.202389	0.433130
C	0.000000	0.000000	-0.281325
H	0.000000	2.121527	-0.147186
H	0.000000	2.150921	2.372219
H	0.000000	-0.000000	3.623260
H	-0.000000	-2.150921	2.372219
H	-0.000000	-2.121527	-0.147186
C	0.000000	0.000000	-1.828408
O	0.000000	-1.144414	-2.359476
O	0.000000	1.144414	-2.359476

CH_3^-

C	0.000000	0.000000	0.166876
H	0.000000	1.002876	-0.333751
H	-0.868516	-0.501438	-0.333751
H	0.868516	-0.501438	-0.333751

CH_2F^-

F	0.082222	-0.736808	0.000000
C	0.082222	0.779675	0.000000
H	-0.616667	0.976609	0.862173
H	-0.616667	0.976609	-0.862173

CHF_2^-

C	0.043984	0.670869	0.000000
H	-1.055625	0.938004	0.000000
F	0.043984	-0.275734	1.134039
F	0.043984	-0.275734	-1.134039

CF_3^-

C	0.000000	0.000000	0.563675
F	0.000000	1.270066	-0.125261
F	-1.099910	-0.635033	-0.125261
F	1.099910	-0.635033	-0.125261

CH_2Cl^-

C	0.055310	1.321382	0.000000
H	-0.636067	1.467787	0.860245

H	-0.636067	1.467787	-0.860245
CL	0.055310	-0.639051	0.000000

$CHCl_2^-$

C	0.026410	0.959226	0.000000
H	-1.056393	1.189252	0.000000
CL	0.026410	-0.204253	1.512964
CL	0.026410	-0.204253	-1.512964

CCl_3^-

C	0.000000	0.000000	0.718396
CL	0.000000	1.709816	-0.084517
CL	-1.480744	-0.854908	-0.084517
CL	1.480744	-0.854908	-0.084517

CH_2Br^-

C	-0.032516	1.744232	0.000000
H	0.666575	1.856326	0.859069
H	0.666575	1.856326	-0.859069
BR	-0.032516	-0.405087	0.000000

$CHBr_2^-$

C	0.014208	1.149508	0.000000
H	-1.079796	1.330047	0.000000
BR	0.014208	-0.117530	1.660902
BR	0.014208	-0.117530	-1.660902

CBr_3^-

C	0.000000	0.000000	0.827527
BR	0.000000	1.885564	-0.047287
BR	-1.632947	-0.942782	-0.047287
BR	1.632947	-0.942782	-0.047287

CH_2I^-

C	0.022989	2.038182	0.000000
H	-0.678164	2.138304	0.860508
H	-0.678164	2.138304	-0.860508
I	0.022989	-0.311428	0.000000

CHI_2^-

C	0.009757	1.285381	0.000000
H	-1.092768	1.445766	0.000000
I	0.009757	-0.086397	1.852407
I	0.009757	-0.086397	-1.852407

CI_3^-

C	0.000070	-0.000001	0.897983
I	-1.049747	1.841089	-0.033820
I	-1.069572	-1.829643	-0.033820
I	2.119310	-0.011446	-0.034018

$CHFCl^{-}$

C	0.720719	0.716389	-0.197287
H	0.708830	1.161475	0.828063
F	1.567195	-0.415389	0.027502
CL	-1.125759	-0.101254	0.006361

$CHFBr^{-}$

C	1.349875	0.723911	-0.193874
H	1.296959	1.134665	0.844777
BR	-0.806124	-0.040628	0.001887
F	2.090901	-0.450684	0.028049

$CHFI^{-}$

C	1.808498	0.719240	-0.191854
H	1.723854	1.111970	0.852758
I	-0.662791	-0.023099	0.000809
F	2.505895	-0.467021	0.028387

$CHClBr^{-}$

C	0.663880	1.016277	-0.187160
H	0.650813	1.391742	0.855150
CL	2.051599	-0.247994	0.009087
BR	-1.128894	-0.093529	0.003238

$CHClI^{-}$

C	1.131660	1.046330	-0.185745
H	1.111155	1.407667	0.863181
CL	2.449532	-0.275546	0.009333
I	-0.934777	-0.056630	0.001748

$CHBrI^{-}$

C	-0.493945	1.195090	-0.183017
H	-0.487860	1.518697	0.879738
BR	-2.076589	-0.136345	0.003472
I	1.436456	-0.073909	0.001827

CF_2Cl^{-}

C	-0.793815	0.000000	0.593369
CL	1.534220	-0.000143	-0.030087
F	-1.184730	-1.052820	-0.169392
F	-1.184031	1.053091	-0.169356

CF_2Br^-

C	-0.688860	1.606654	0.000000
F	0.077984	1.911573	-1.041683
F	0.077984	1.911573	1.041683
BR	0.077984	-1.258521	0.000000

CF_2I^-

C	-0.712535	-2.134468	0.000000
F	0.060214	-2.413494	1.041205
F	0.060214	-2.413494	-1.041205
I	0.060214	1.061315	0.000000

CCl_2F^-

C	-0.242270	0.781567	0.000000
F	1.076755	1.189707	0.000000
CL	-0.242270	-0.452846	1.530584
CL	-0.242270	-0.452846	-1.530584

CCl_2Br^-

C	0.920571	0.160185	0.000000
CL	0.920571	-0.997170	1.472722
CL	0.920571	-0.997170	-1.472722
BR	-1.052082	0.941219	0.000000

CCl_2I^-

C	0.781383	-0.983518	0.000000
CL	-0.053888	-1.773023	1.468895
CL	-0.053888	-1.773023	-1.468895
I	-0.053888	1.248753	0.000000

CBr_2F^-

C	-0.136523	1.082659	0.000000
BR	-0.136523	-0.284686	-1.707862
BR	-0.136523	-0.284686	1.707862
F	1.152858	1.492448	0.000000

CBr_2Cl^-

C	-0.314009	0.853646	0.000000
BR	-0.314009	-0.452074	1.642636
BR	-0.314009	-0.452074	-1.642636
CL	1.403805	1.560196	0.000000

CBr_2I^-

C	0.882470	0.319087	0.000000
BR	0.882470	-0.960860	1.625566

BR	0.882470	-0.960860	-1.625566
I	-1.265428	1.232937	0.000000

CI_2F^-

C	0.093896	1.281827	0.000000
I	0.093896	-0.219964	1.928869
I	0.093896	-0.219964	-1.928869
F	-1.168483	1.736135	0.000000

CI_2Cl^-

C	-0.221432	1.087590	0.000000
I	-0.221432	-0.357407	1.850155
I	-0.221432	-0.357407	-1.850155
CL	1.458848	1.844680	0.000000

CI_2Br^-

C	0.448975	0.828039	0.000000
I	0.448975	-0.594432	1.839129
I	0.448975	-0.594432	-1.839129
BR	-1.436718	1.658330	0.000000

$CBrClF^-$

C	0.707580	0.598451	0.653468
CL	1.928959	-0.710036	-0.063906
BR	-1.282428	-0.177042	-0.023775
F	0.871909	1.630710	-0.222475

$CIClF^-$

C	1.225747	0.605299	0.650869
CL	2.319359	-0.783667	-0.066645
I	-1.128020	-0.091299	-0.013537
F	1.444609	1.614378	-0.228309

$CIBrF^-$

C	-0.538551	0.954931	0.664980
I	1.545640	-0.179565	-0.015111
BR	-2.089016	-0.391481	-0.027451
F	-0.619116	1.943246	-0.247581

$CIBrCl^-$

C	0.500900	0.550741	0.797190
CL	0.777460	2.136007	-0.113663
BR	1.896931	-0.757667	-0.044130
I	-1.558770	-0.247136	-0.024648
